# Structure-Function Correlation Analysis of Connexin50 Missense Mutations Causing Congenital Cataract: Electrostatic Potential Alteration Could Determine Intracellular Trafficking Fate of Mutants

**DOI:** 10.1155/2014/673895

**Published:** 2014-05-06

**Authors:** Devroop Sarkar, Kunal Ray, Mainak Sengupta

**Affiliations:** ^1^Department of Genetics, University of Calcutta, University College of Science, 35 Ballygunge Circular Road, Kolkata 700 019, India; ^2^Academy of Scientific and Innovative Research (AcSIR), Coordination Office, CSIR-CRRI, CRRI P.O., Delhi-Mathura Road, New Delhi 110 025, India

## Abstract

Connexin50 (Cx50) mutations are reported to cause congenital cataract probably through the disruption of intercellular transport in the lens. Cx50 mutants that undergo mistrafficking have generally been associated with failure to form functional gap junction channels; however, sometimes even properly trafficked mutants were found to undergo similar consequences. We hereby wanted to elucidate any structural bases of the varied functional consequences of Cx50 missense mutations through in silico approach. Computational studies have been done based on a Cx50 homology model to assess conservation, solvent accessibility, and 3-dimensional localization of mutated residues as well as mutation-induced changes in surface electrostatic potential, H-bonding, and steric clash. This was supplemented with meta-analysis of published literature on the functional properties of connexin missense mutations. Analyses revealed that the mutation-induced critical alterations of surface electrostatic potential in Cx50 mutants could determine their fate in intracellular trafficking. A similar pattern was observed in case of mutations involving corresponding conserved residues in other connexins also. Based on these results the trafficking fates of 10 uncharacterized Cx50 mutations have been predicted. Further experimental analyses are needed to validate the observed correlation.

## 1. Introduction

The intercellular transport of ions and metabolites via an extensive network of gap junctions is indispensible for the growth, development, and proper maintenance of the mammalian avascular lens. Connexins (Cx) are the only gap junction proteins identified in the lens to date. The three isoforms of the connexins expressed in the lens are Cx43, Cx46, and Cx50 [1]. After being synthesized in the endoplasmic reticulum (ER) membrane, the connexin subunits oligomerize to form a hemichannel (or connexon) that is then delivered to the plasma membrane. The end-to-end docking of two hemichannels forms a functional gap junction channel. Combination of different connexin isomers during channel formation can lead to homotypic, heterotypic, or heteromeric channels [1, 2].

Mutations in the* GJA8* gene, coding for Cx50 (NP_005258.2), have been associated with congenital cataract (OMIM *600897) in humans. To date 28 Cx50 mutations have been identified to be associated with congenital cataract of which 26 are missense. While most of these mutations precipitate the disease following an autosomal dominant pattern, at least one missense mutation (i.e., V196M) is known to be causal only in homozygous condition [3]. So far functional studies of 12 missense mutants (11 human Cx50 mutations and 1 mouse Cx50 mutation) have revealed that gap junction activity is altered in most cases (except G46V and I247M) and that may or may not be associated with altered trafficking of the mutant proteins (Table 1). Six of those (i.e., R23T, D47N, P88S, P88Q, Gja8-R205G, and E201K) have been found to be mislocalized in the cytoplasm, ER, ER-Golgi intermediate compartment (ERGIC), and/or Golgi apparatus leading to a lack of gap junction plaque formation with a consequential lack of hemichannel currents, gap junctional conductance, and/or dye transfer activities. However, in 2 cases (i.e., V44E and W45S) properly localized mutants have been found to fail to form functional gap junctional channels. Further, different mutants have been reported to have variable effects on co-expressed wild type connexins, often acting as dominant negative inhibitors, which probably explain causation of the disease in heterozygous condition. However, the reported functional studies are not all inclusive; that is, in a few studies with the mutant connexins, their intracellular trafficking, gap junction conductance/dye transfer activities, and effects on wild-type (WT) proteins have not been studied together, thus creating a gap in the understanding of the precise molecular mechanism that lead to congenital cataract for each of the mutations. This wide functional heterogeneity of Cx50 missense mutations calls for a structure-function correlation approach.

In spite of several attempts in the past to elucidate the structure-function correlation of gap junction channels not much progress could be achieved in this field before the resolution of the 3-dimensional structure of Cx26 at the atomic level by Maeda et al. [4] in 2009. Still, not much is known about the functional role of distinct residues in the connexin molecules that upon mutation could lead to various intracellular consequences precipitating disease phenotypes. In this context, we hereby attempt to delineate the structural alterations, if any, that could potentially determine the various functional properties of Cx50 missense mutations by meta-analysis of available literature supplemented by in silico studies. Currently in silico approach has emerged as a powerful tool to explore biological systems. Molecular dynamics simulation studies have provided valuable structural insights into the pathomechanism of various diseases and even drug resistance mechanisms of different microorganisms [5–14]. As the crystal structure of human Cx50 is not yet resolved in atomic resolution we pursued our studies on a homology model of human Cx50.

## 2. Materials and Methods

### 2.1. Homology Modeling

A homology model of Cx50 (residues 3–230) was generated by the SWISS-MODEL Workspace (http://swissmodel.expasy.org/) [15], in automated mode. The crystal structure of the human Cx26 gap junction channel (PDB: 2ZW3) was selected as a template by the intrinsic template search algorithm of the SWISS-MODEL platform. Cx50 shares 52% sequence similarity with Cx26 (multiple sequence alignment provided as Supplementary Text in Supplementary Material available online at http://dx.doi.org/10.1155/2014/673895). The quality of the model was evaluated by the ProSA server (https://prosa.services.came.sbg.ac.at/prosa.php) [16]; the *z*-score of the structure (−3.6) is within the range of scores typically found for native proteins of similar size. The structure was subjected to energy minimization (200 steps of steepest descent) with a partial implementation of the GROMOS96 force field energy (http://www.gromos.net/) included in the Swiss-PdbViewer software v4.1.0 (a.k.a. DeepView) (http://spdbv.vital-it.ch/) [17] to improve the van der Waals contacts and to correct the stereochemistry of the model. All analyses were done using this final structure.

### 2.2. Docking Analysis

As already stated above, Cx50 oligomerizes to form connexons. So we wanted to find out whether the residues involved in disease-causing mutations were also involved in protein-protein interactions in the normal hexamer structure. The SymmDock server (http://bioinfo3d.cs.tau.ac.il/SymmDock/) [18] was used for symmetrical protein-protein docking to predict the probable structure of the hexameric complex using the homology model of the connexin. From the ten top-scoring solutions in the SymmDock output, the solution that was most close to the Cx26 hexamer structure (PDB: 2ZW3) was chosen for analysis.

It must be mentioned here that in the present study we restricted our computational analyses to the homomeric homotypic channel formed by Cx50; that is, we have not studied protein-protein interactions between Cx50 and other connexin isoforms (Cx43 and Cx46) in mixed type channels.

### 2.3. Conservation Analysis

A multiple sequence alignment was constructed by the ConSurf Server (http://consurf.tau.ac.il/) using UNIREF90 (http://www.uniprot.org/help/UniRef) and CLUSTALW (http://embnet.vital-it.ch/software/ClustalW.html); 150 nonredundant homologous protein sequences selected by PSI-BLAST (http://www.ncbi.nlm.nih.gov/BLAST/) were aligned to assess conservation status of the residues. PyMOL v1.3 (http://www.pymol.org/) [19] was used to visualize the ConSurf colored structure.

### 2.4. Structural Analysis

All structural analyses were done using the Swiss-PdbViewer software v4.1.0. The mutations were induced in the modeled structure one at a time using the mutate tool of the Swiss-PdbViewer software and analyses were done based on the “best” rotamer of the new amino acid. We examined (a) localization in the structure, (b) solvent accessibility of the residues, (c) change of surface electrostatic potential, (d) gain/loss of H-bonds, and (e) induction of steric clash.

## 3. Results and Discussion

Missense mutants differ from the WT protein by only a single residue. In case of *α*-helix bundle proteins like Cx50, depending upon the location of the mutated residue in the protein chain, a missense mutation can have varied consequences—disruption of monomer folding or of its oligomerization properties; further, mutations involving docking surfaces may prevent the docking between two originally compatible hemichannels. Indeed, previous functional studies of 12 Cx50 missense mutants revealed that those differed with respect to intracellular trafficking, hemichannel currents, gap junctional conductance/dye transfer activities, and dominant negative effects on WT Cx50. In the present study we wanted to elucidate the structural bases, if any, that determine whether or not a mutant would undergo proper trafficking to cell membrane and, if trafficked properly, whether or not it would lead to complete or partial alteration of gap junction channel conductance.

**Figure 1:**
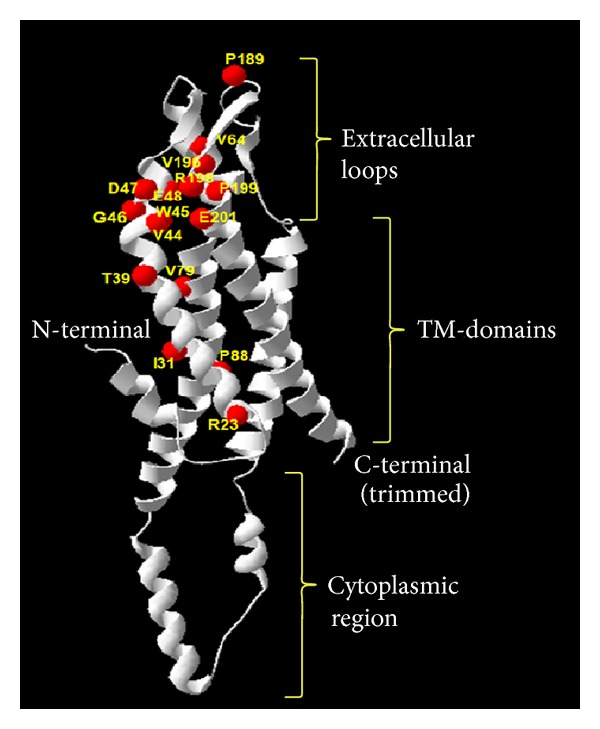
Distribution of the amino acid residues involved in disease-causing mutations in the Cx50 homology model. Ribbon view of the Cx50 homology model is shown (constructed using the SWISS-MODEL server). Different regions of the protein are marked (N-terminal, TM-transmembrane domains, extracellular loops, cytoplasmic region, and C-terminal). Sixteen residues that are point-mutated to cause congenital cataract are indicated in the homology model (red spheres).

Twenty missense mutations involving 15 residues could be localized within our homology model of Cx50 (Figure 1). Most of the plotted mutations mapped to the first transmembrane domain (TM1) and the first extracellular loop (EC1). To date, 11 missense mutations in human Cx50 associated with congenital cataract have been functionally characterized; their molecular consequences are furnished in Table 1. It is to be noted that although R198W/R198Q human Cx50 mutants have not been functionally characterized, R205G involving the corresponding residue in mouse Gja8 has been characterized and in accordance we have included the R198 residue and the R198W/R198Q mutations in our analyses.

**Figure 2:**
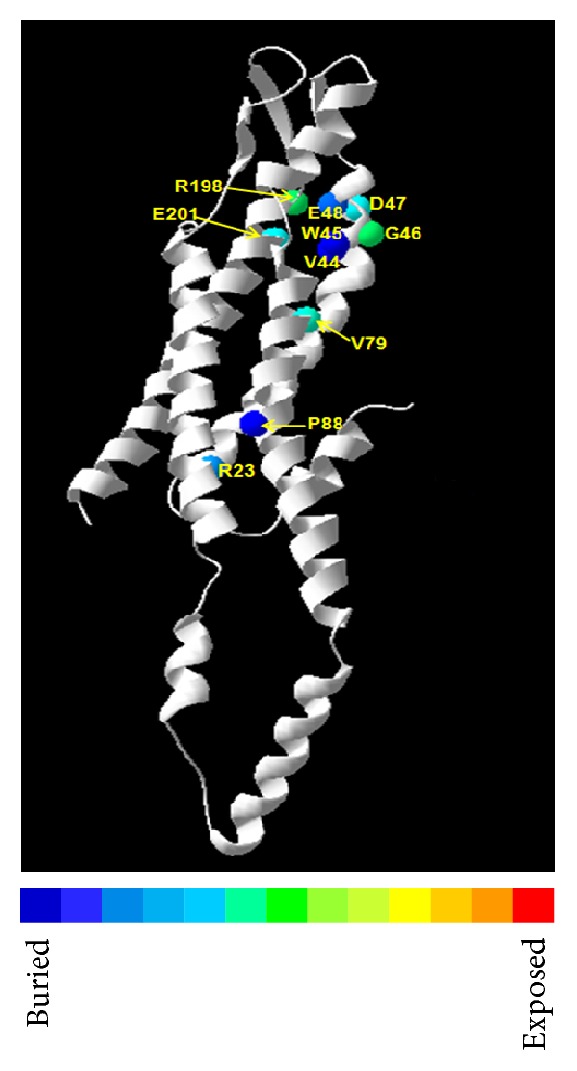
Three-dimensional location of the functionally characterized Cx50 mutations in the homology model of the wild-type protein. Ribbon view of the Cx50 homology model is shown. Ten residues involved with 11 missense changes are represented as spheres colored according to solvent accessibility (using the Swiss-PdbViewer—blue through red corresponding to buried-through-exposed residues; see color bar). Seven mutations (R23T, V44E, W45S, D47N, E48K, and P88S/Q) with loss of gap junction conductance involved 6 structurally buried residues (blue spheres). Other 2 mutation-involving residues are relatively exposed (G46 and R198 [equivalent to mouse R205]) (green spheres). E201 (blue sphere) represents the mistrafficked mutant E201K (gap junctional conductance has not been reported yet).

In silico analyses revealed that all of the functionally characterized human Cx50 missense mutations alter highly conserved residues (Supplemental Figure 1; multiple sequence alignment provided as supplementary text). Among the 11 mutations (10 human and 1 mouse Cx50 mutations) that could be plotted, 7 mutations failing to form functional gap junctional channels involved structurally buried residues (Figure 2). Mutations involving buried residues in the transmembrane domains might disrupt monomer folding (*α*-helix conformation) and thereby destabilize the Cx50 monomer ultimately affecting oligomerization and intracellular trafficking.

Altogether 8 mutations are reported to cause complete lack of gap junction channel conductance (i.e., R23T, V44E, W45S, D47N, E48K, P88S/Q, and Gja8-R205G) (Table 1). Among these, 5 mutations undergo impaired intracellular trafficking (i.e., R23T, D47N, P88S/Q, and R205G[Gja8]) while 2 mutations (i.e., V44E and W45S) traffic properly to cell membrane; trafficking of E48K has not been reported yet. In addition to these, E201K has recently been reported to undergo mistrafficking although gap junctional conductance for this mutation has not been reported to date.

Next, we wanted to assess each mutation individually and correlate the mutation-induced structural alterations to the intracellular molecular consequences.

### 3.1. Individual Assessment of the Missense Mutations Revealed the Following Observations

#### 3.1.1. Improperly Trafficked Mutations Leading to Complete Loss of Gap Junction Channel Conductance

**Figure 3:**
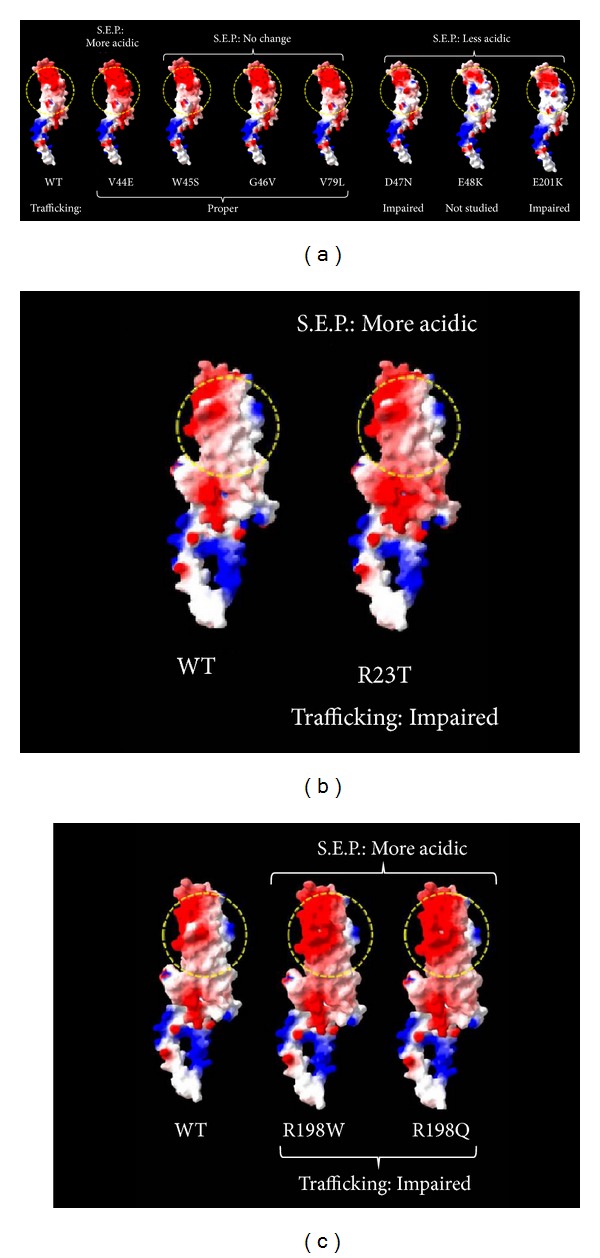
Changes in surface electrostatic potential in the functionally characterized Cx50 mutations. The molecular surface is colored according to electrostatic potential using the Swiss-PdbViewer, with red-white-blue corresponding to acidic-neutral-basic potential. WT: wild type Cx50. S.E.P.: surface electrostatic potential. (a) Mutations in TM1-EC1 junction (V44E, W45S), EC1 (G46V, D47N, and E48K), TM2 (V79L), and EC2-TM4 junction (E201K); (b) mutation in TM1 (R23T); (c) mutations in EC2-TM4 junction (R198W/Q). W45S, G46V, and V79L involve no change in surface electrostatic potential; the surface electrostatic potential of D47N, E48K, and E201K is less acidic than WT; in the case of R23T, V44E, R198W, and R198Q, the surface electrostatic potential is more acidic than WT. All mutants (except V44E) with changes in surface electrostatic potential exhibit impaired trafficking. Yellow dotted circle represents the region of significant alteration of SEP.

*R23T*. Thomas et al. [20] reported that R23T exhibits impaired intracellular trafficking and is localized to the cytoplasm instead of cell membranes. Consequently, R23T does not lead to any significant gap junctional conductance or dye transfer activity. Further, this mutant has an inhibitory effect on coexpressed WT Cx50. Our analysis revealed that R23 (located in TM1) is involved in interhelix H-bonding with side chain of Y151 (TM3) (Supplemental Figure 2). R23T disrupts H-bonding with Y151, thereby potentially destabilizing the interhelix interaction; but no intramolecular steric clash is induced. This mutation also leads to acidification of surface electrostatic potential (Figure 3) of the Cx50 molecule. In the oligomeric complex the side chain R23 (TM1) points towards the subunit (Supplemental Figure 3). However the mutation R23T does not induce any steric clash or alteration of H-bonds with any residue of the adjacent subunit.

*D47N*. It is reported to undergo impaired trafficking at physiological temperature and lead to no detectable intercellular conductance [21, 22]. However, it does not inhibit coexpressed WT Cx50 and also forms functional heteromeric channels with Cx46 [21]. D47N (located in EC1) does not alter the H-bonding pattern of the molecule or induce any steric clash but reduces the acidic potential (Figure 3) of the transmembrane domains of Cx50. In the oligomeric complex, R76 and Q49 of adjacent subunit lie within surrounding 4 Å space (Supplemental Figure 4); no H-bonding is involved. Upon mutation, no induction of steric clash or alteration of H-bonds takes place with any residue of the adjacent subunit. 

*P88S/P88Q*. Both P88S and P88Q lead to defective trafficking and, as expected, functional homotypic gap junction channel is not formed. Further, both these mutants inhibit WT Cx50 when coexpressed [23–25]. P88 (located in TM2) is responsible for the highly conserved kinked conformation of the TM2. Substitution of the proline by any other residue would disrupt the kinked conformation of the TM-domain of the molecule and is thus expected to be misfolded and mislocalized. P88S and P88Q do not involve alteration in H-bonding pattern or surface electrostatic potential.

*R205G[Gja8]*. As mentioned earlier, although R205G in Gja8 has been characterized in mouse model, the mutations in the corresponding residue in human Cx50 (i.e., R198W and R198Q) have not been functionally characterized to date. It has been found that R205G[Gja8] fails to form normal gap junction channels and has an inhibitory effect on coexpressed WT Cx50 and Cx46 proteins [26]. R205G[Gja8] involves substitution of a basic residue (Arg) by an aliphatic residue (Gly), thereby predictably leading to acidification of surface electrostatic potential. This residue is located at the junction of EC2 and TM4. Our studies with the corresponding human Cx50 mutations revealed that R198Q leads to loss of 1 H-bond and gain of another H-bond while R198W leads to a loss of 1 H-bond (Supplemental Figure 5). R198W/R198Q mutations were also found to alter the surface electrostatic potential (more acidic) due to substitution by an aromatic residue (Trp) or a polar residue (Gln) (Figure 3). None of the mutations involve induction of any steric clash. R198 is involved in H-bonding with E48 of adjacent subunit; upon mutation to R198Q this H-bond is replaced with a new H-bond with S73 and upon mutation to R198W the H-bond with E48 is lost; steric clash is induced in cases of both R198Q and R198W (Supplemental Figure 6).

#### 3.1.2. Properly Trafficked Mutations Leading to Complete Loss of Gap Junction Channel Conductance

*V44E*. This mutant (located in TM1-EC1 junction) has been reported to undergo proper intracellular trafficking and form gap junction plaques but does not form functional homotypic gap junction channels and also inhibited coexpressed WT Cx50 and Cx46 in a dominant negative fashion [21]. While V44E causes the surface electrostatic potential to be more acidic (Figure 3), it does not disrupt/induce any H-bonds or induce any steric clash. In the oligomeric complex, R76 of adjacent subunit lies within surrounding 4 Å space (Supplemental Figure 4); no H-bonding is involved. Upon mutation, no induction of steric clash or alteration of H-bonds takes place with any residue of the adjacent subunit.

*W45S*. This mutant (located in TM1-EC1 junction) has been found to have similar intracellular trafficking consequences as V44E; it also inhibited coexpressed WT Cx50 and Cx46 in a dominant negative fashion [27]. However, W45S does not alter either the surface electrostatic potential (Figure 3) or H-bonding pattern or induce any steric clash. In the oligomeric complex, side chain of W45 is directed away from the intersubunit interface (Supplemental Figure 4); thereby upon mutation, no induction of steric clash or alteration of H-bonds takes place with any residue of the adjacent subunit.

#### 3.1.3. Properly Trafficked Mutation Leading to Unaltered Gap Junction Channel Conductance

*G46V*. G46V (located in EC1) traffics properly and shows similar gap junction conductance but higher hemichannel currents than WT Cx50 probably leading to apoptosis of the cells [27, 28]. G46V involves no alteration of electrostatic potential (Figure 3) or H-bonding or induction of steric clash. Interestingly, we found that G46 is a relatively exposed residue, unlike the rest of the neighboring mutation-involving residues (Figure 2); further, G46V faces the channel pore (Supplemental Figure 7) and probably is not involved in oligomerization; consequently it does not involve induction of steric clash or alteration of H-bonds with any residue of the adjacent subunit.

#### 3.1.4. Properly Trafficked Mutation Leading to Altered Gap Junction Channel Conductance

*V79L*. This mutant (located in TM2) has been reported to traffic properly and form functional homotypic and heteromeric intercellular channels (with Cx46 and Cx50); however, it caused alteration in voltage gating and a dramatic reduction in the single-channel open probability, resulting in reduced levels of conductance [21]. The variation involves substitution by an aliphatic residue (similar to the WT residue) thus leading to no alteration of surface electrostatic potential (Figure 3). Also this mutant does not alter H-bonding of the molecule or induce any steric clash. V79 (TM2) lies close to intersubunit interface with T39 of adjacent subunit lying within surrounding 4 Å space; however, there is no intersubunit H-bonding (Supplemental Figure 8). Upon mutation, there is no induction of steric clash or alteration of H-bonds with any residue of the adjacent subunit.

#### 3.1.5. Improperly Trafficked Mutation without Any Report of Gap Junction Channel Conductance

*E201K*. Recently E201K (located in EC2-TM4 junction) has been reported to mislocalize in the cytoplasm [29] although gap junctional conductance for this mutation has not been studied. As the mutant fails to localize in the plasma membrane it is expected that it would be unable to form functional gap junction channels. This mutant alters surface electrostatic potential from acidic to basic (Figure 3) and leads to gain of 1 H-bond (Supplemental Figure 9) but causes no induction of steric clash. In the oligomeric complex, E201 lies in close association with P71, I72, S73, and R76 of adjacent subunit (Supplemental Figure 10); E201 forms intermolecular H-bonding with S73 of adjacent subunit that is not disrupted upon mutation; also no steric clash is induced upon mutation.

**Figure 4:**
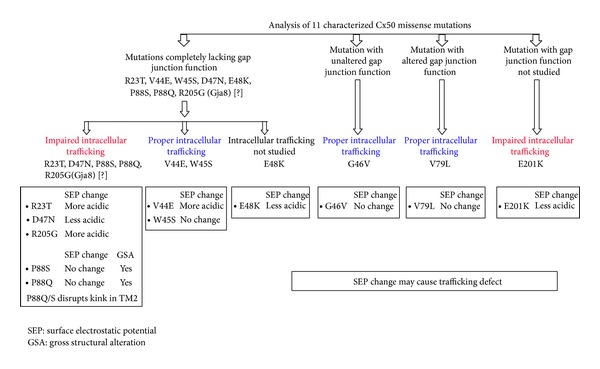
Correlation of alterations in the surface electrostatic potential in different Cx50 mutants with their intracellular trafficking fates. Four out of 6 mutations showing impaired trafficking (R23T, D47N, E201K, and R205G[Gja8]) involve S.E.P. change, while the other 2 mutations (P88S and P88Q) involve gross structural alteration. On the contrary, only 1 out of 4 mutations showing proper trafficking (V44E) involves S.E.P. change.

As mentioned above, R23T, R198Q/W, and E201K involve H-bond alteration; only the mutations R198Q/W involve induction of steric clash while several mutations involved changes in surface electrostatic potential. We tried to correlate the alterations in the surface electrostatic potential in different mutants with their intracellular trafficking fates (Figure 4). It is evident that, while 4/6 mutants showing impaired trafficking (R23T, D47N, E201K, and R205G[Gja8]) involve change in surface electrostatic potential, the other 2 mutants (P88S and P88Q) involve gross structural alteration (loss of kink in TM2). On the contrary, only 1/4 mutants showing proper trafficking (V44E) involves surface electrostatic potential alteration. This observation is suggestive of the fact that surface electrostatic potential alteration could be a potential determinant of the intracellular trafficking fate of Cx50 missense mutants.

Surface electrostatic potential change can hamper intersubunit interactions (i.e., oligomerization), thereby disrupting normal trafficking of the hexamer to the cell membrane. In fact, Thomas et al. [20] through mutating R23 with acidic, neutral, and basic residues had reported that N-terminal positive charge of the connexin protein is critically important for proper trafficking and gap junction channel conductance. The above argument helps us predict that R198W/R198Q mutations would probably lead to impaired trafficking and nonfunctional gap junction, considering that these account for alteration in both intramolecular and intermolecular H-bonding pattern, as well as surface electrostatic potential alteration and induction of steric clash. However, it can explain neither the fate of V44E trafficking nor the cause of loss of channel conductance in the case of properly trafficked W45S.

From the different trafficking fates of 4 mutations involving consecutive residues in the junction of TM1 and EC1 (i.e., V44E, W45S, G46V, and D47N), it can be presumed that the acidification of surface electrostatic potential in this region has no disruptive effect on oligomerization and consequently trafficking (as in case of V44E) as opposed to reduction in acidic potential in the same region (as in case of D47N) (Figure 3). With reference to oligomerization, side chains of V44 and D47 are placed in the same region of space (Supplemental Figure 4); this further supports our argument on the importance of negative potential in this particular region of the protein.

For the E48K mutation, located in EC1, intracellular trafficking effects have not yet been determined. Banks et al. [30] reported that E48K shows no gap junction conductance and has an inhibitory role on the gap junction channel conductance of coexpressed WT Cx50 but not on WT hemichannel conductance. Further, the E48K mutation leads to loss of an H-bond (with R76 of TM2) (Supplemental Figure 11) and grossly alters the surface electrostatic potential of the protein (Figure 3) but does not induce any steric clash. Interestingly, E48K might also lead to loss of intermolecular H-bonding with R198 of adjacent subunit (Supplemental Figure 12). Based on our earlier argument, we predict that the E48K mutant will exhibit impaired trafficking, which could be responsible for the loss of gap junction activity.

We attempted to assess whether our observed correlation between surface electrostatic potential alterations with intracellular trafficking in Cx50 holds true for other connexins also. A few Cx26 and Cx32 mutations have been functionally characterized till date that involve corresponding conserved residues as the Cx50 mutations investigated further in this study. Cx32-R22G and Cx32-R22P (corresponding to Cx50-R23T) have been found to show loss of gap junction channel conductance [31]; although any information about their intracellular trafficking fate is unavailable, these mutations also involve acidification of surface electrostatic potential as Cx50-R23T. Similarly, Cx26-W44S and Cx26-W44C (corresponding to Cx50-W45S) have been functionally characterized; Cx26-W44S shows altered gap junction channel function (reduced dye transfer efficiency and dominant negative effect on coexpressed WT Cx26 and Cx30) [32] and Cx26-W44C shows impaired intercellular coupling in spite of proper trafficking to the plasma membrane [33]. Thus neither of these mutants alters surface electrostatic potential, and hence, both of these traffic properly (as Cx50-W45S). Cx26-G45E (corresponding to Cx50-G46V) has been found to form hemichannels with significantly elevated whole cell currents than WT; however Cx26-G45E gap junction channels have similar efficiency as WT Cx26 [34]. Cx26-G45E leads to alteration (acidification) of surface electrostatic potential; consequently, a similar argument as Cx50-V44E might be applicable here (i.e., acidification of surface electrostatic potential in this particular region of the molecule might have a benign impact on trafficking fate). Again, Cx26-D46E (corresponding to Cx50-D47N) was found to traffic properly although it showed disruption of both ionic and biochemical coupling [35]. Cx26-D46E does not alter the surface electrostatic potential as it involves substitution by similarly charged residue, in contrast to Cx50-D47N that reduces electrostatic potential and undergoes impaired trafficking. In case of Cx32-E186K (corresponding to Cx50-E201K) the mutant was found to undergo mistrafficking and be exclusively localized in the Golgi apparatus [36]. Cx32-E186K alters surface electrostatic potential in an exactly similar way as Cx50-E201K since both mutants involve the same residue substitution.

Thus overall, surface electrostatic potential alteration seems to have a determining role in trafficking fate of connexin mutants; while in majority of the cases alteration of surface electrostatic potential seems to cause impaired intracellular trafficking, in some particular regions of the connexin molecule acidification of surface electrostatic potential might have a benign effect on trafficking. The meager number of characterized mutants makes it imperative to perform further in vitro studies to validate the observed correlation. However, the ultimate effect on the gap junction channel function cannot be explained by our model; properly trafficked mutants are found to form gap junction channels with altered properties depending upon the substitution of residues. It is noteworthy that the phenomenon of impaired membrane trafficking due to alteration of electrostatic potential was previously reported in case of an opsin mutation as well [37].

We have also studied the structural alterations in the 9 functionally uncharacterized pathogenic Cx50 mutations that could be localized in our Cx50 homology model (Table 2). Thereby we have attempted to predict their functional consequences based on our observed correlation discussed above. Five mutants, I31T (TM1), V64G (EC1), P189L (EC2), V196M (EC2), and P199S (EC2), do not alter surface electrostatic potential of the molecule; hence they would probably undergo proper trafficking. Four mutants, T39R (TM1), G46R, D47H, and D47Y (TM1-EC1 junction), involve alteration of surface electrostatic potential and T39R also involves steric clash induction with adjacent subunit; those would probably undergo impaired trafficking.

## 4. Conclusions

We have found an interesting correlation between surface electrostatic potential alterations with intracellular trafficking fate of Cx50 missense mutants. Considering the fact that in silico approach has its inherent limitations of not considering the global changes that could only be studied in solution, our observation needs to be validated by further comprehensive experimental studies. Nevertheless, this approach can help the elucidation of the molecular pathogenesis of congenital cataract and also can pave the way for similar analyses for other diseases involving gap junction proteins.

## Supplementary Material

Supplementary Material includes Supplemental Figures (1-12: additional figures supporting the text) and Text (i. sequence alignment of human Cx50 protein with the template used for homology modeling, and, ii. multiple sequence alignment for conservation analysis of Cx50).

## Figures and Tables

**Table 1:** Summary of the functional parameters previously reported for 12 disease-causing Cx50 missense mutations.

Sl. number	Mutation	Functional study			Ref. number
		Trafficking (localization, if reported)	Gap junction channel	Inhibitory to WT Cx50	
1	R23T	Impaired (cytoplasm)	N.F.	Yes	[20]
2	V44E	Proper	N.F.	Yes	[21]
3	W45S	Proper	N.F.	Yes	[27]
4	G46V	Proper	F. (conductance similar to WT)	No	[27, 28]
5	D47N	Impaired (cytoplasm) [proper trafficking at 27°C.]	N.F.	No	[22]
		Impaired	N.F.	No	[21]
6	E48K	[Not studied] Predicted: impaired	N.F.	Yes	[30]
7	V79L	Proper	F. (lower conductance)	Yes	[21]
8	P88S	[Not studied]	N.F.	Yes	[23]
		Impaired (cytoplasm and appositional membranes)	N.F.	Yes	[24]
9	P88Q	Impaired (cytoplasm) [proper trafficking at 27°C.]	N.F.	Yes	[25]
10	R205G(Gja8)* R198W/Q	Impaired Predicted: Impaired	N.F. Predicted: N.F.	Yes —	[26] —
11	E201K	Impaired (cytoplasm)	[Not studied] Predicted: N.F.	[Not studied]	[29]
12	I247M	Proper	F. (conductance similar to WT)	[Not studied]	[38]

F.: functional; N.F.: nonfunctional; ER: endoplasmic reticulum.

*R205(Gja8) is functionally characterized in mouse model. R205(Gja8) corresponds to R198(GJA8) in human Cx50, bearing mutations R198Q and R198W, neither of which is functionally characterized.

**Table 2:** Structural parameters studied in 9 functionally uncharacterized pathogenic Cx50 mutations.

Sl. number	Mutation	Location in protein	Monomer			Oligomeric complex	
			Change in electrostatic potential	H-bonding alteration	Steric clash induction	Side chain orientation—towards intersubunit interface	Steric clash induction with adjacent subunit
1	I31T	TM1	No	No change	No	Yes	No
2	T39R	TM1	Yes	No change	No	Yes	Yes
3	G46R	TM1-EC1 junc.	Yes	No change	No	Yes (R46)	No
4	D47H	TM1-EC1 junc.	Yes	Loss of 1 H-bond	Yes	Yes	No
5	D47Y	TM1-EC1 junc.	Yes	Loss of 1 H-bond	No	Yes	No
6	V64G	EC1	No	No change	No	No	No
7	P189L	EC2	No	No change	No	No	No
8	V196M	EC2	No	No change	No	No	No
9	P199S	EC2	No	No change	No	No	No

TM: transmembrane domain; EC: extracellular loop.
