# Supplementary material for: Structure-Function Correlation Analysis of Connexin50 Missense Mutations Causing Congenital Cataract: Electrostatic Potential Alteration Could Determine Intracellular Trafficking Fate of Mutants
Source: Biomed Res Int. 2014 May 6;2014:673895. doi: 10.1155/2014/673895 (PMC4066682; doi:10.1155/2014/673895)
Supplement: Supplementary file 1 — Supplementary Material includes Supplemental Figures (1-12: additional figures supporting the text) and Text (i. sequence alignment of human Cx50 protein with the template used for homology modeling, and, ii. multiple sequence alignment for conservation analysis of Cx50). [file 673895.f1.pdf]

SUPPLEMENTARY FIGURES:

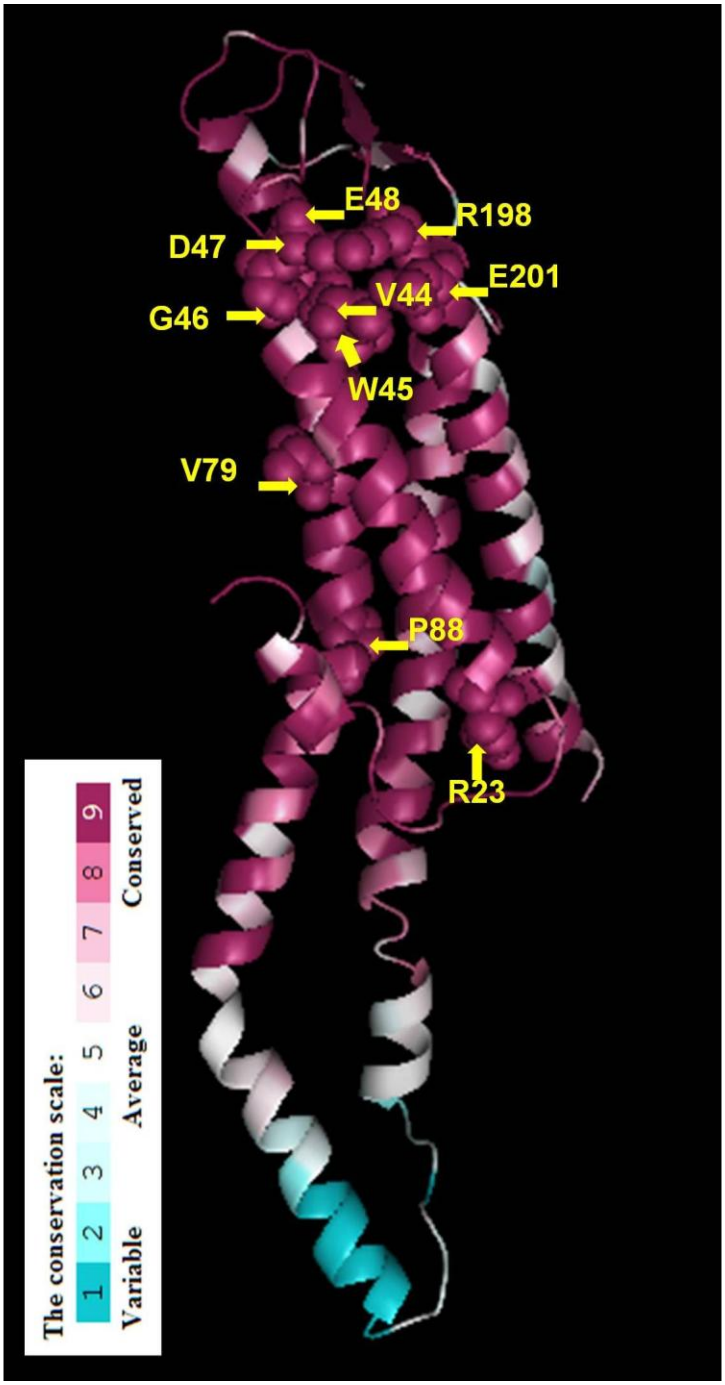

Supplemental Figure 1.

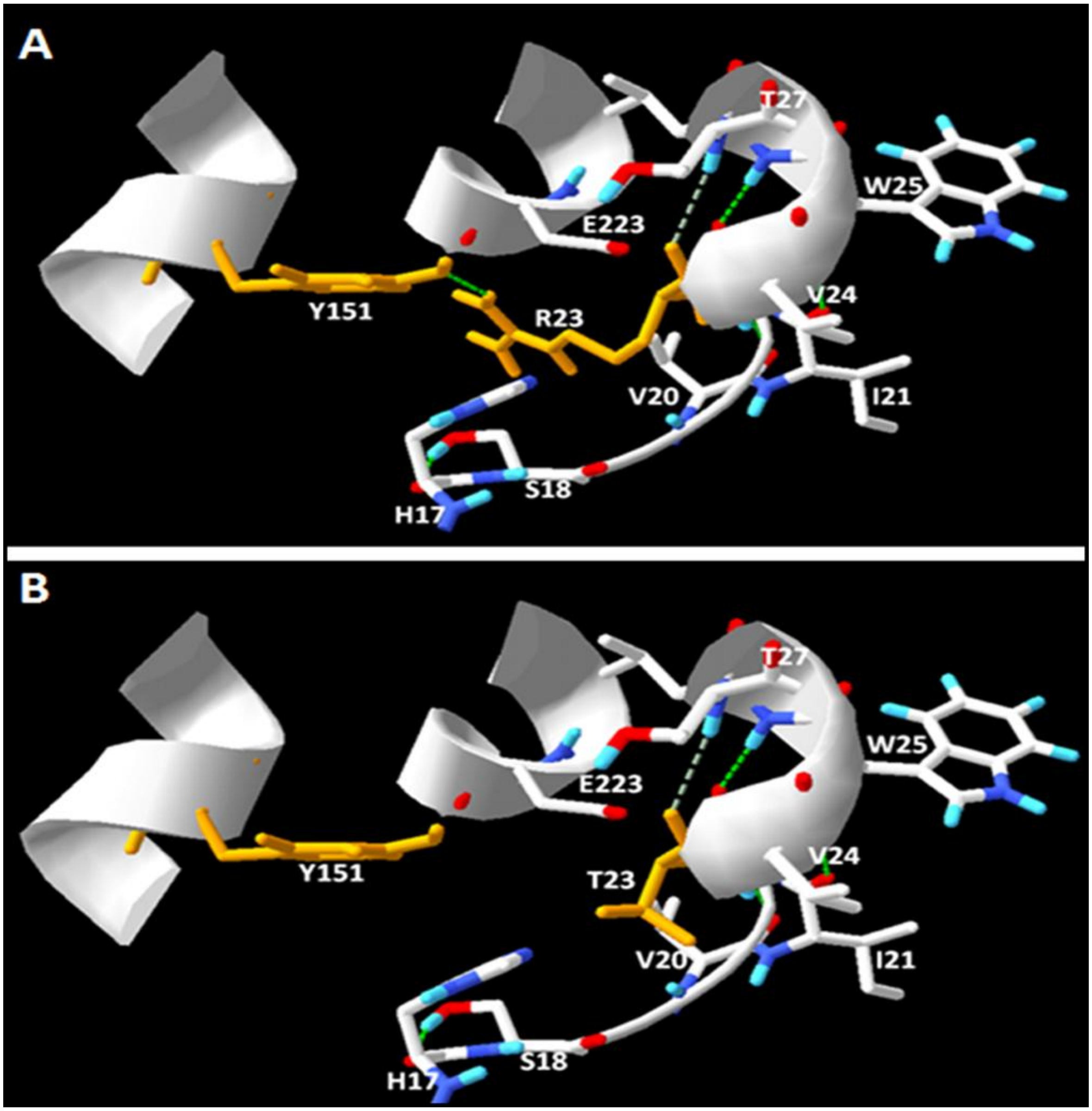

Supplemental Figure 2.

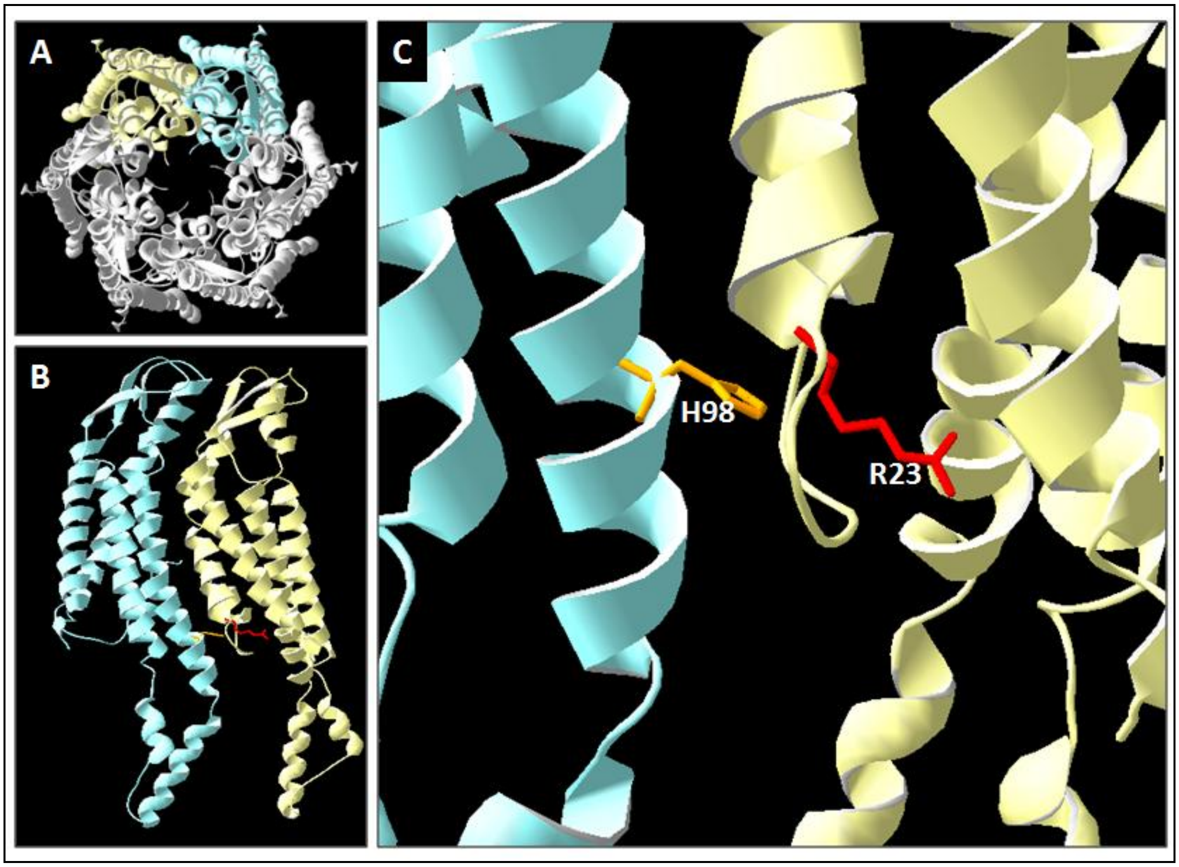

Supplemental Figure 3.

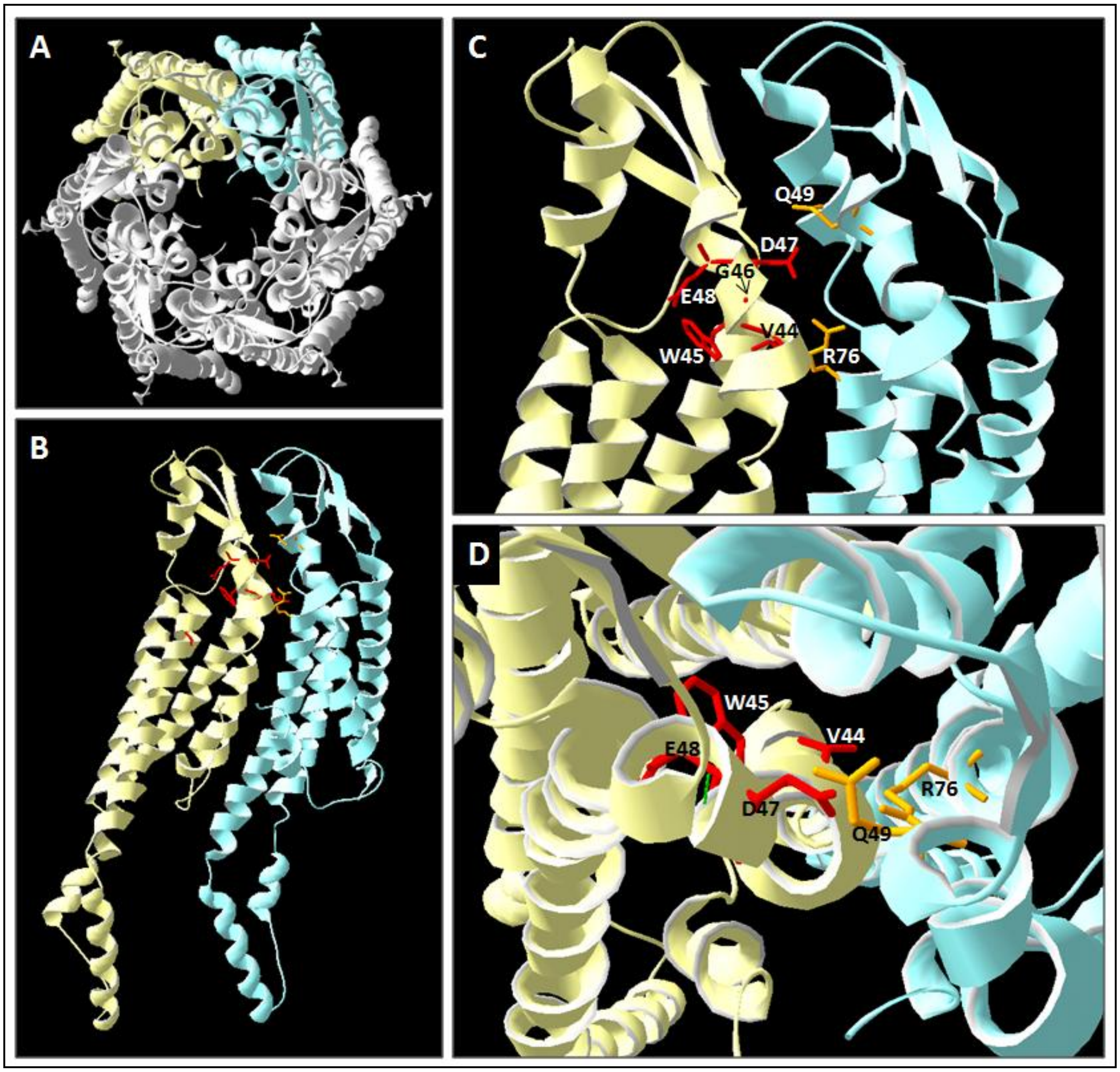

Supplemental Figure 4.

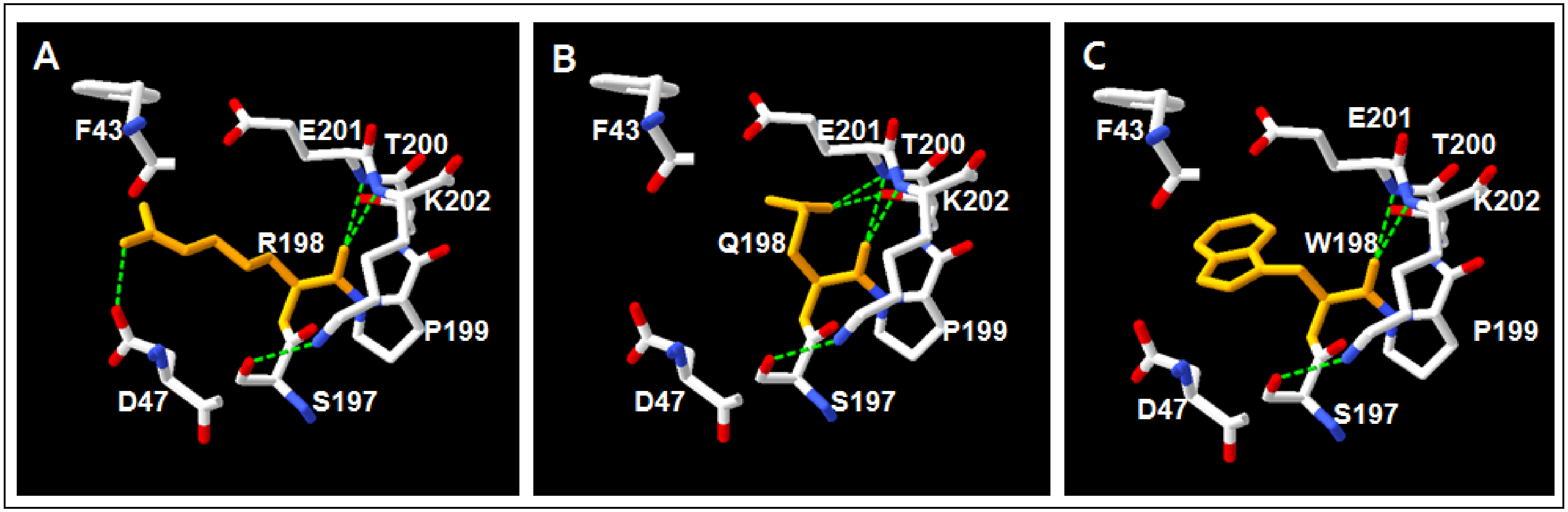

Supplemental Figure 5.

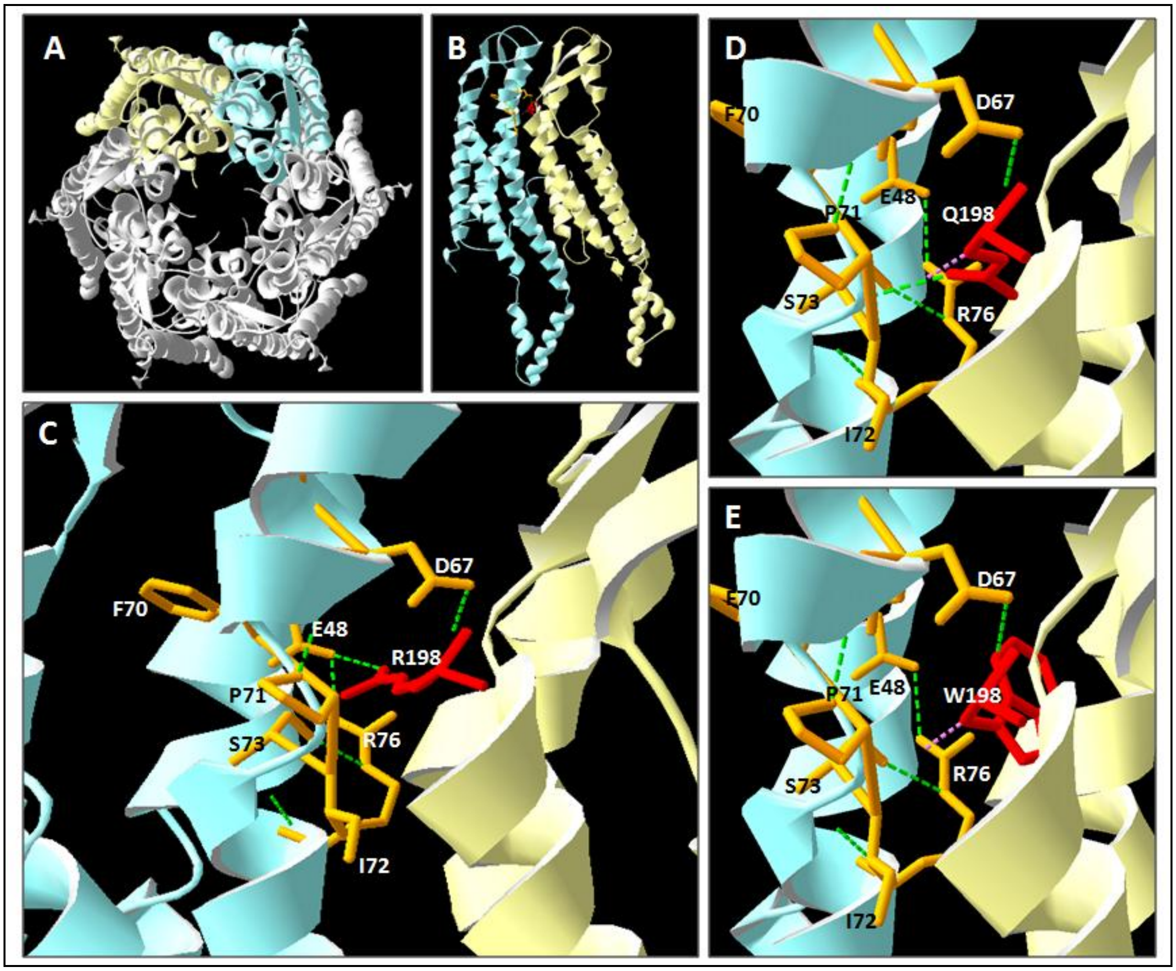

Supplemental Figure 6.

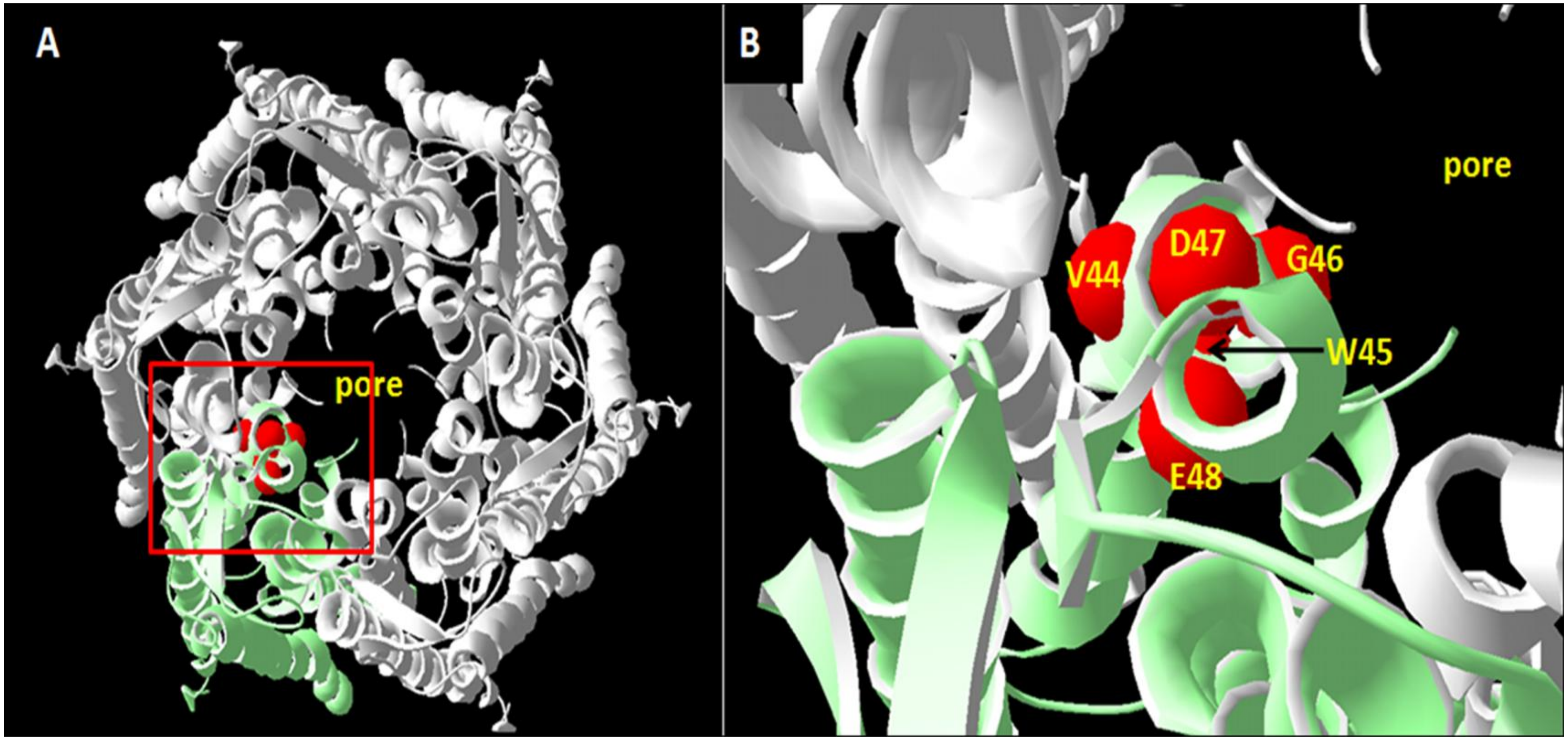

Supplemental Figure 7.

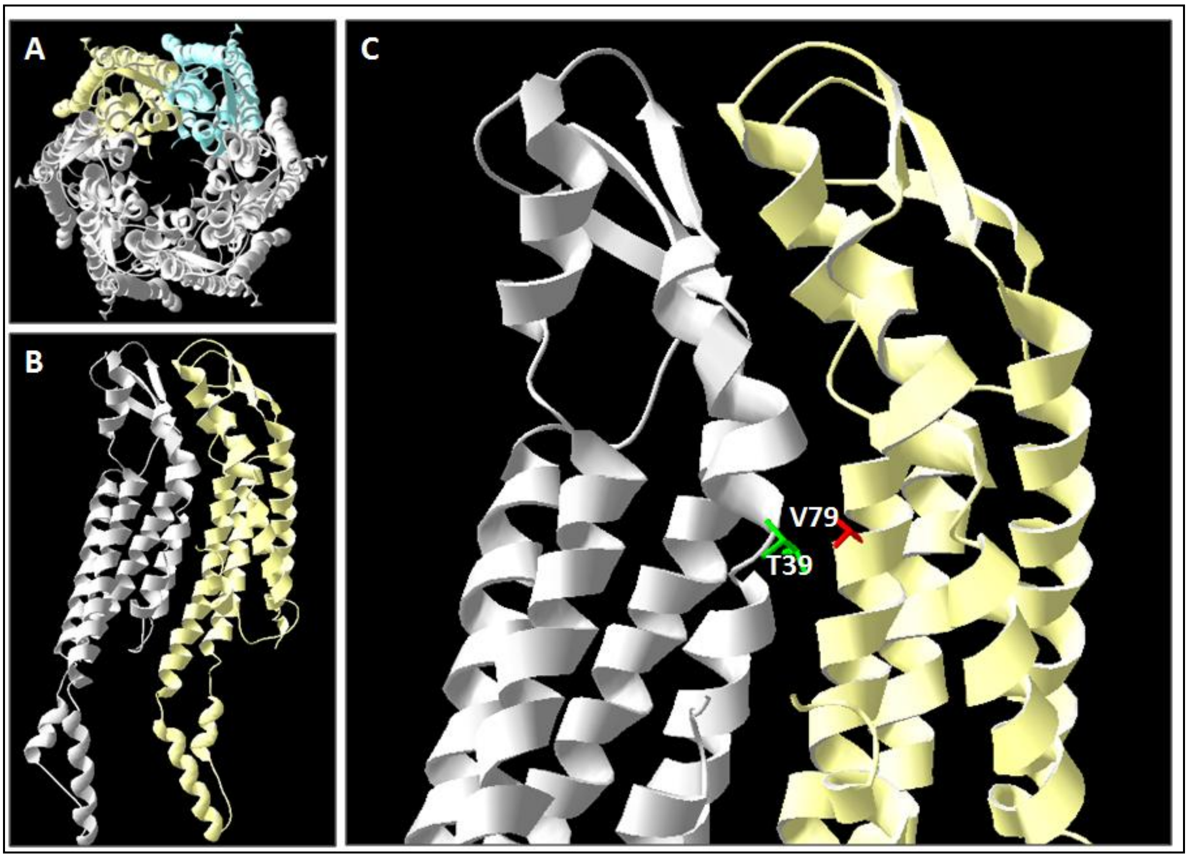

Supplemental Figure 8.

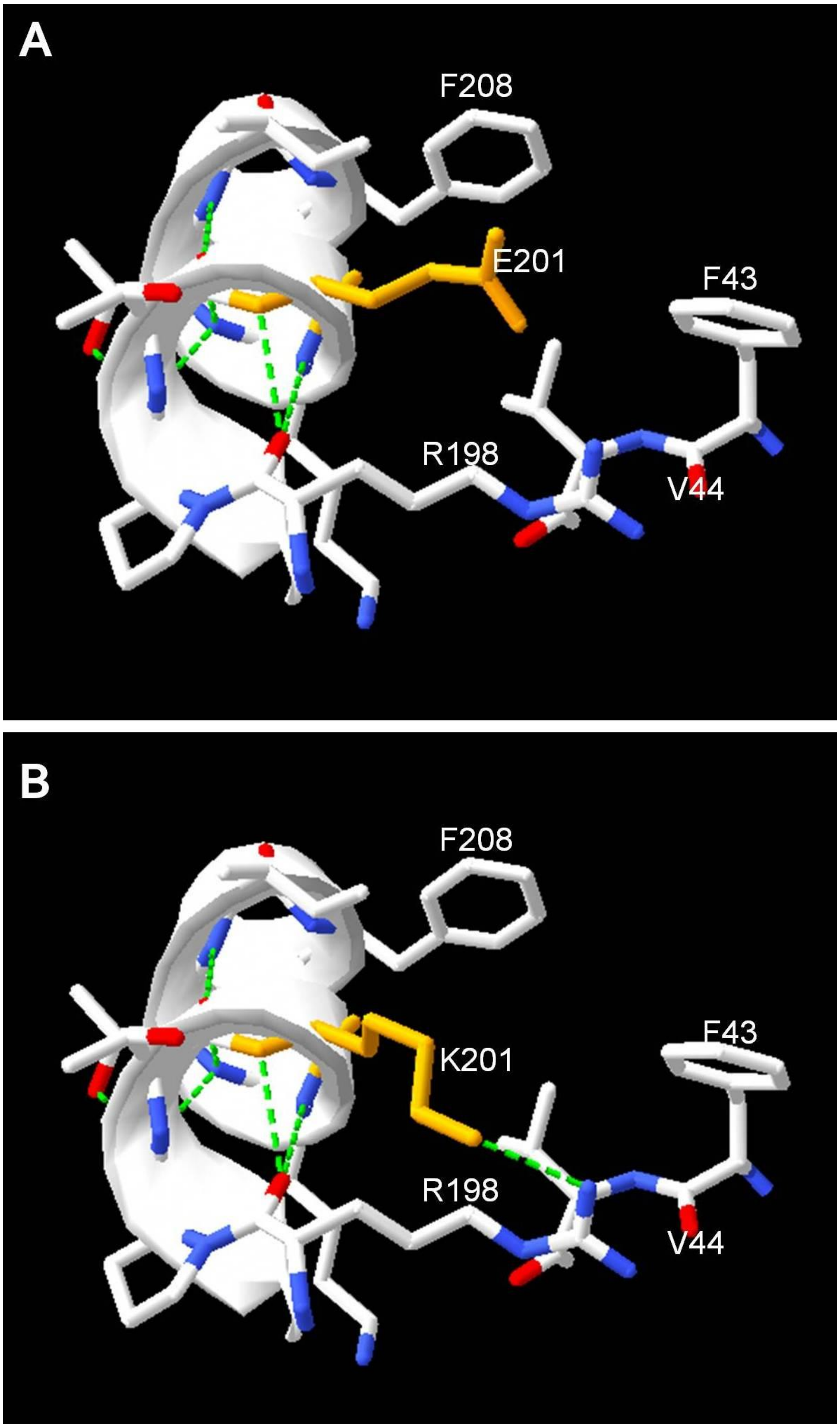

Supplemental Figure 9.

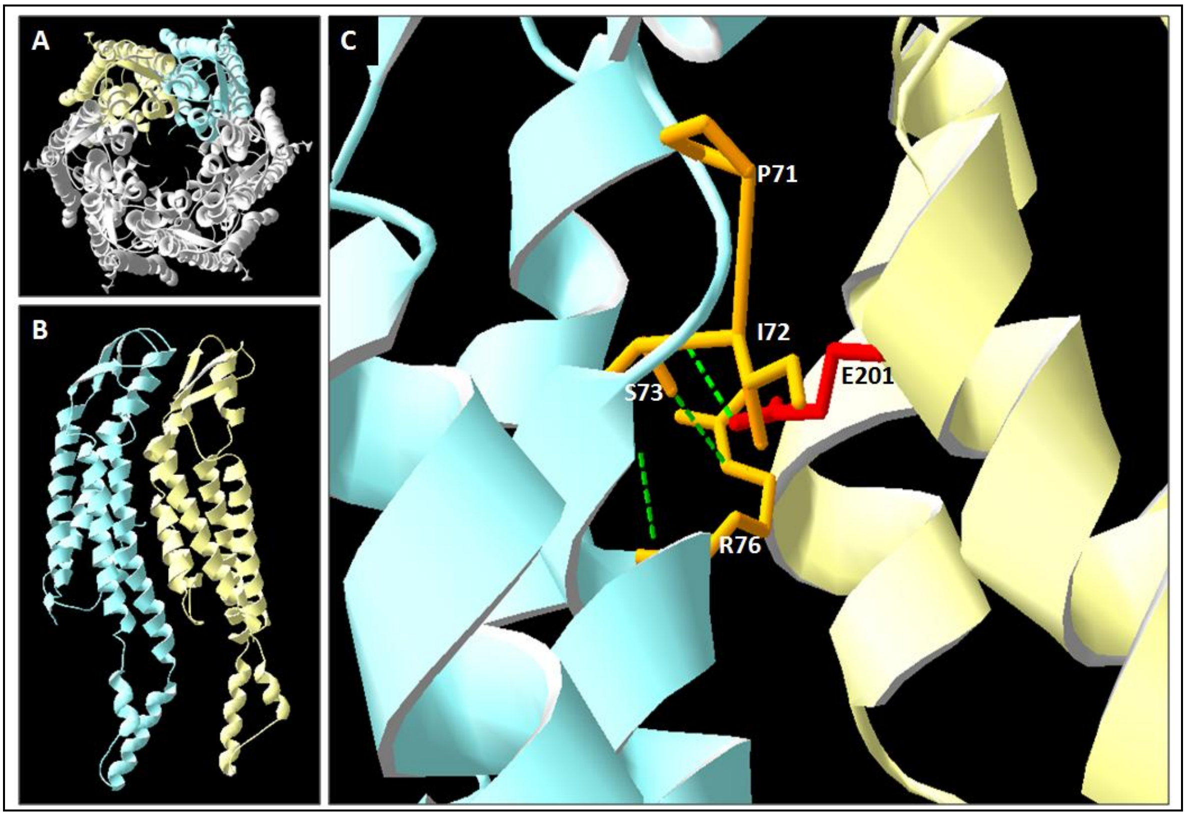

Supplemental Figure 10.

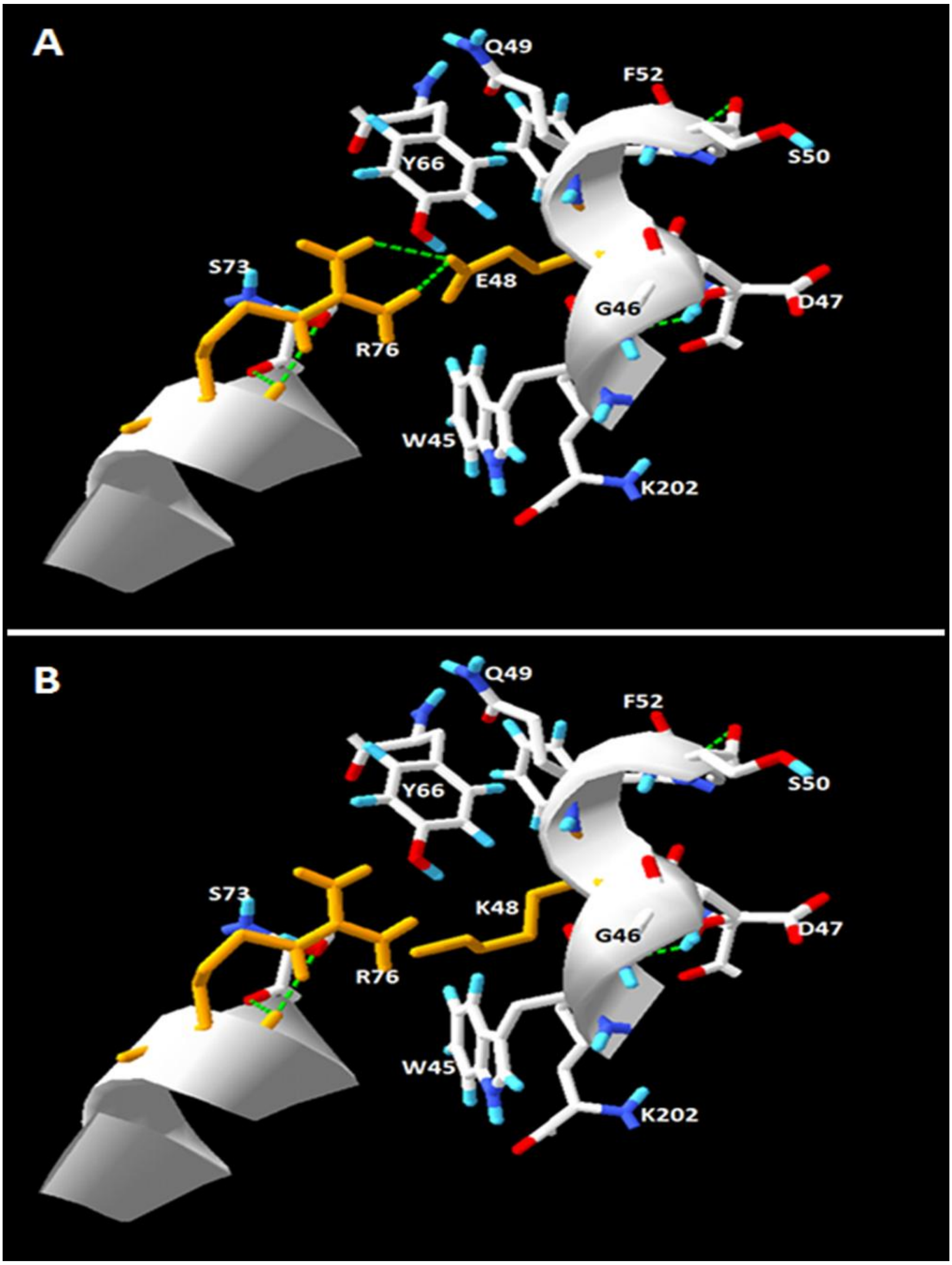

Supplemental Figure 11.

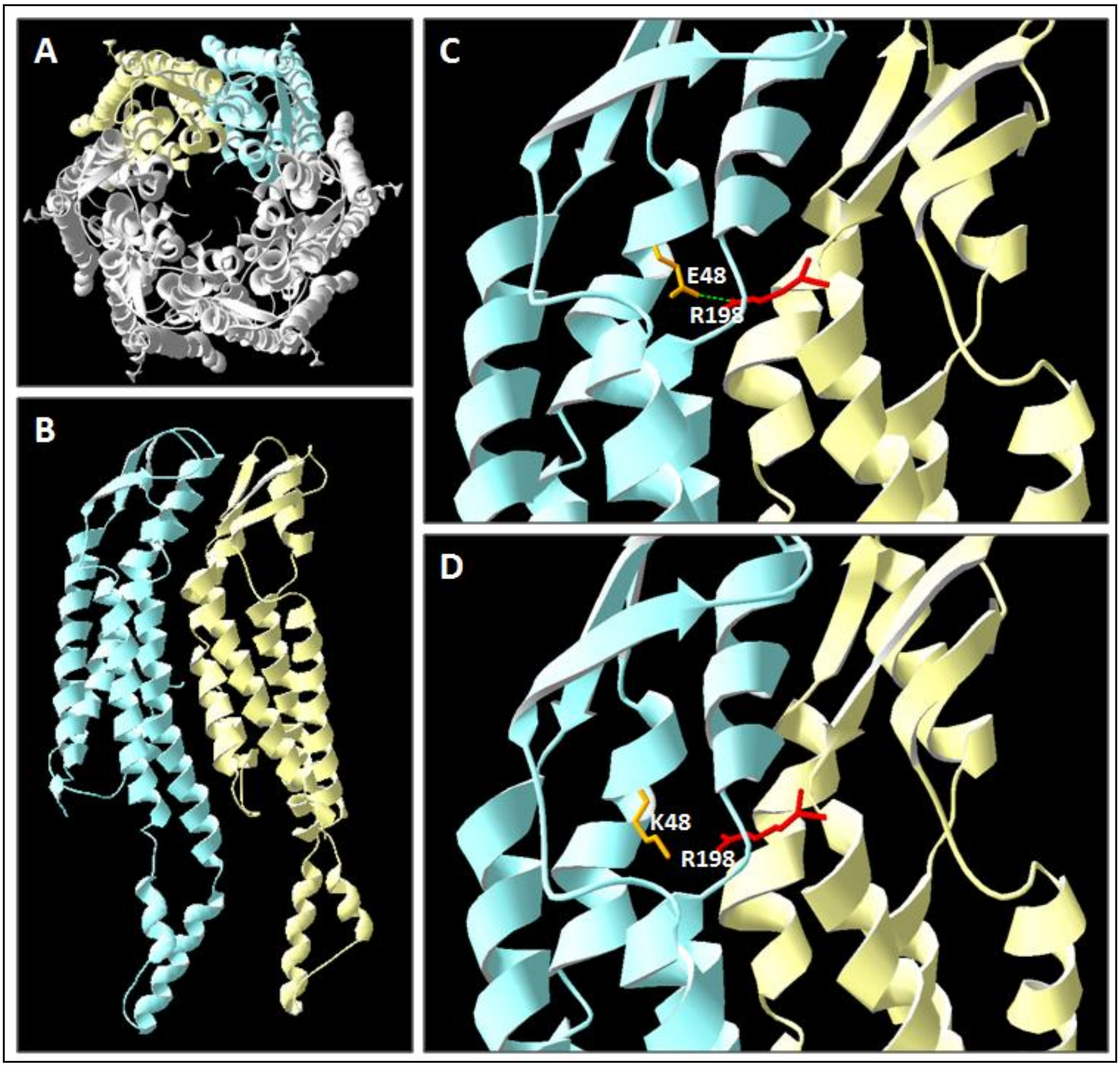

Supplemental Figure 12.

SUPPLEMENTARY FIGURE LEGENDS:

Supplemental Figure 1. The homology model of Cx50 color-coded according to evolutionary conservation.

Ribbon view of the Cx50 homology model is shown with the mutation-involving residues plotted on it. The structure is color-coded according to evolutionary conservation using the ConSurf server, with turquoise-through-maroon corresponding to variable-through-conserved positions (see color bar). PyMOL was used for visualization. All of the functionally characterized Cx50 mutations involve highly conserved residues. *Check supplementary text for multiple sequence alignment*.

Supplemental Figure 2. The 3-dimensional neighborhood (surrounding 4Å space) of R23 in normal Cx50 protein (homology model).

A. Residues lying within surrounding 4Å space of R23 are shown. R23 (TM1) has inter-helix H-bonding with side-chain of Y151 (TM3). B. The H-bond of R23 with Y151 is lost in the mutant R23T. Green and grey dotted lines indicate potential H-bonds (strong and weak, respectively) - computed using the SwissPDBViewer.

Supplemental Figure 3. The 3-dimensional neighborhood of R23 in the oligomeric complex.

A. Docked model of hemichannel – viewed from the top. Two adjacent Cx50 subunits are colored. B. Two adjacent Cx50 subunits are shown to be arranged in similar relation as in the hexameric complex – viewed from outer lipid-facing side. C. Mutation-involving residue R23 (red) in one subunit (yellow) and H98 (orange) of adjacent subunit (blue) lie within 4Å space.

Supplemental Figure 4. The 3-dimensional neighborhood of V44, W45, G46, D47 and E48 in the oligomeric complex.

A. Docked model of hemichannel – viewed from the top. Two adjacent Cx50 subunits are colored. B. Two adjacent Cx50 subunits are shown to be arranged in similar relation as in the hexameric complex – viewed from pore side. C. Mutation-involving residues 44-48 are shown (red) in one subunit (yellow); two residues (R76 & Q49 - orange) in adjacent subunit (blue) within surrounding 4Å space of the mutation-involving residues are shown. D. The intermolecular interface (involving residues 44-48 of one chain and R76 & Q49 of adjacent chain) is shown – viewed from the top. Side-chains of V44 & D47 point outwards, facing adjacent subunit; side-chains of W45 & E48 are directed towards the subunit. There is no inter-subunit H-bonding involving the residues.

Supplemental Figure 5. The 3-dimensional neighborhood (surrounding 4Å space) of R198 in normal Cx50 protein (homology model).

A. Residues lying within surrounding 4Å space of R198 are shown. B. R198Q leads to loss of 1 H-bond with D47 and gain of another H-bond with T200/E201. C. R198W leads to a loss of 1 H-bond with D47. Green lines indicate potential H-bonds - computed using the SwissPDBViewer.

Supplemental Figure 6. The 3-dimensional neighborhood of R198 in the oligomeric complex.

A. Docked model of hemichannel – viewed from the top. Two adjacent Cx50 subunits are colored. B. Two adjacent Cx50 subunits are shown to be arranged in similar relation as in the hexameric complex – viewed from outer lipid-facing side. C. Neighbouring residues in adjacent subunit (orange) that lie within surrounding 4Å space of R198 (red) are shown. R198 might form H-bond with E48 of adjacent subunit. Green dotted lines indicate potential H-bond - computed using the SwissPDBViewer. D. R198Q – the H-bond with E48 is

replaced with a new H-bond with S73; steric clash induced (pink dotted line). E. R198W – the H-bond with E48 is lost; steric clash induced.

Supplemental Figure 7. The 3-dimensional neighborhood of G46 in the oligomeric complex.

A. Docked model of hemichannel (constructed by SymmDock server) formed by symmetric docking of Cx50 subunits (Cx50 homology models). A single Cx50 subunit is colored light-green. Mutation-involving residues (red spheres) in the junction of TM1 and EC1 are localized in the docked model. B. Zoomed view of the residue-positions. G46 is a pore-exposed residue.

Supplemental Figure 8. The 3-dimensional neighborhood of V79 in the oligomeric complex.

A. Docked model of hemichannel – viewed from the top. Two adjacent Cx50 subunits are colored. B. Two adjacent Cx50 subunits are shown to be arranged in similar relation as in the hexameric complex – viewed from pore side. C. Mutation-involving residue V79 (red) in one subunit (yellow) and T39 (green) of adjacent subunit (grey) lie within 4Å space.

Supplemental Figure 9. The 3-dimensional neighborhood (surrounding 4Å space) of E201 in normal Cx50 protein (homology model).

A. Residues lying within surrounding 4Å space of E201 are shown. B. A new H-bond with side-chain of R198 is formed by the mutation E201K. Green dotted line indicates potential H-bond - computed using the SwissPDBViewer.

Supplemental Figure 10. The 3-dimensional neighborhood of E201 in the oligomeric complex.

A. Docked model of hemichannel – viewed from the top. Two adjacent Cx50 subunits are colored. B. Two adjacent Cx50 subunits are shown to be arranged in similar relation as in

the hexameric complex – viewed from outer lipid-facing side. C. P71, I72, S73 and R76 (orange) of adjacent subunit (blue) lie within surrounding 4Å space of E201 (red). E201 forms inter-molecular H-bonding with S73 of adjacent subunit.

Supplemental Figure 11. The 3-dimensional neighborhood (surrounding 4Å space) of E48 in normal Cx50 protein (homology model).

A. Residues lying within surrounding 4Å space of E48 are shown. E48 (TM1) has inter-helix H-bonding with R76 (TM2). B. The H-bond of E48 with R76 is lost in the mutant E48K. Green dotted lines indicate potential H-bonds H-bonds - computed using the SwissPDBViewer.

Supplemental Figure 12. The 3-dimensional neighborhood of E48 in the oligomeric complex.

A. Docked model of hemichannel – viewed from the top. Two adjacent Cx50 subunits are colored. B. Two adjacent Cx50 subunits are shown to be arranged in similar relation as in the hexameric complex – viewed from outer lipid-facing side. C. R198 (red) of adjacent subunit (yellow) lies within surrounding 4Å space of E48 (orange). E48 has inter-molecular H-bonding with R198 of adjacent subunit. Green dotted line indicates potential H-bond - computed using the SwissPDBViewer. D. The H-bond of E48 with R198 is lost in the mutant E48K.

SUPPLEMENTARY TEXT:

CLUSTAL 2.1 multiple sequence alignment constructed by CLUSTALW2 server – 52% sequence similarity between human Cx50 protein sequence and human Cx26 protein sequence, which was used as template *(PDB: 2ZW3)* for homology modeling

```
2ZW3            -MDWGTLQTILGGVNKHSTSIGKIWLTVLFIFRIMILVVAAKEVWGDEQADFVCNTLQPG 59
Cx50            MGDWSFLGNILEEVNEHSTVIGRVWLTVLFIFRILILGTAAEFVWGDEQSDFVCNTQQPG 60
                  **. * .**  **:*** **::**********:** .**: ******:****** ***

2ZW3            CKNVCYDHYFPISHIRLWALQLIFVSTPALLVAMH-VAYRRHEKKRKFIKGEIKSEFK-- 116
Cx50            CENVCYDEAFPISHIRLWVLQIIFVSTPSLMYVGHAVHYVRMEEKRKSREAEELGQQAGT 120
                *:*****. *********.**:******:*: . * * * * *:***  :.*  .:

2ZW3            ----DIEEIK-------TQKVRIEGSLWWTYTSSIFFRVIFEAAFMYVFYVMYDGFSMQR 165
Cx50            NGGPDQGSVKKSSGSKGTKKFRLEGTLLRTYICHIIFKTLFEVGFIVGHYFLYG-FRILP 179
                    *  .:*       *:*.*:**:*  ** . *:*:.:**..*:  .*.:*. * :

2ZW3            LVKCNAWPCPNTVDCFVSRPTEKTVFTVFMIAVSGICILLNVTELCYLLIRYCSGKSKKP 225
Cx50            LYRCSRWPCPNVVDCFVSRPTEKTIFILFMLSVASVSLFLNVMELGHLGLKGIRSALKRP 239
                * :*. *****.************:* :**::*:.:.::*** ** :* ::   .  *:*

2ZW3            V 226
Cx50            V 240
                *
```

CLUSTAL 2.0.10 multiple sequence alignment constructed by UNIREF90 – 150 non-redundant sequences homologous to human Cx50 were selected by PSI-BLAST and aligned

Input_protein_seq denotes human Cx50 protein sequence (NP_005258.2)

```
UniRef90_UPI0002C691BE_1_358      MGDWSVLGEFLEEVHKHSTVIGKVWLTVLFIFRILVLGAAAESSWGDEQE
UniRef90_F7BKC0_1_358             MGDWSYLAEFLEEVHKHSTEIGKVWLTVLFIFRILVLVTAAESSWGDEQA
UniRef90_Q0VCR2_1_359             MGDWSFLGEFLEEVHKHSTVIGKVWLTVLFIFRMLVLGTAAESSWGDEQA
UniRef90_G3TUX5_1_358             MGDWSFLGEFLEEVHKHSTVIGKVWLTVLFIFRMLVLGTAAESSWGDEQA
UniRef90_L9JKN2_1_358             MGDWSFLGEFLEEVHKHSTVIGKVWLTVLFIFRMLVLGTAAESSWGDEQA
UniRef90_P36382_1_358             MGDWSFLGNFLEEVHKHSTVVGKVWLTVLFIFRMLVLGTAAESSWGDEQA
UniRef90_UPI0003315E33_1_350      MGDWSFLGEFLEEVHKHSTVIGKVWLTVLFIFRMLVLGTAAESSWGDEQA
UniRef90_UPI000333FB64_1_358      MGDWSFLGEFLEEVHKQSTVIGKVWLTVLFIFRMLVLGTAAESSWGDEQA
UniRef90_UPI000334540D_1_357      MGDWSFLGEFLEEVHKHSTVIGKVWLTVLFIFRMLVLGTAAESSWGDEQA
UniRef90_L5K3R8_1_358             MGDWSFLGEFLEEVHKHSTVIGKVWLTVLFIFRMLVLGTAAESSWGDEQA
UniRef90_G1TQ37_1_358             MGDWSFLGEFLEEVHKHSTVIGKVWLTVLFIFRMLVLGTAAESSWGDEQS
UniRef90_UPI0001776F40_1_352      MGDWSFLGEFLEEVHKHSTVIGKVWLTVLFIFRMLVLGTAAESSWGDEKA
UniRef90_G3H2Q9_1_358             MGDWSFLGEFLEEVHKHSTVIGKVWLTVLFIFRMLVLGTAAESSWGDEQA
UniRef90_Q01231_1_358             MGDWSFLGEFLEEVHKHSTVIGKVWLTVLFIFRMLVLGTAAESSWGDEQA
UniRef90_G5BBL6_1_357             MSDWSFLGAFLEEVHKHSTIIGKVWLTVLFIFRMLVLGTAAESSWGDEQA
UniRef90_H0WCZ7_1_358             MSDWSFLGAFLEEVHKHSTVIGKVWLTILFIFRMLVLGTAAESSWGDEQA
UniRef90_G3X1A3_1_358             MGDWSFLGEFLEEVHKHSTVIGKVWLTVLFIFRMLVLGTAAESSWGDEQA
UniRef90_H0ZTI1_1_370             MGEWSFLGDFLEKVHKHSTVVGKVWLTVLFIFRMLVLGTAAESSWGDEQS
UniRef90_P18860_1_369             MGDWSFLGEFLEEVHKHSTVVGKVWLTVLFIFRMLVLGTAAGPLWGDEQS
UniRef90_G1KPB5_1_370             MGDWSFLGEFLEEVHKHSTVVGKVWLTVLFIFRMLVLGTAAESSWGDEQS
UniRef90_K7FWT6_1_369             MDNWSFLGEFLEEVHKHSTVVGKVWLTVLFVFRMLVLGTAAESSWGDEQS
UniRef90_F1QL21_1_400             MGDWSLLGNFLEEVQEHSTSVGKVWLTVLFIFRILVLGTAAESSWGDEQS
UniRef90_UPI0002A497DC_1_408      MADWSLLGNFLEEVQEHSTSVGKVWLTILFIFRILVLGTAAESSWGDEQE
Input_protein_seq                 MGDWSFLGNILEEVNEHSTVIGRVWLTVLFIFRILILGTAAEFVWGDEQS
UniRef90_UPI00027FC10C_1_397      MGDWSFLGSILEEVNEHSTVIGRVWLTVLFIFRILILGTAAEFVWGDEQS
UniRef90_L9JGQ0_1_441             MGDWSFLGNILEEVNERSTVIGRVWLTVLFIFRILILGTAAEFVWGDEQS
UniRef90_P28236_1_440             MGDWSFLGNILEEVNEHSTVIGRVWLTVLFIFRILILGTAAEFVWGDEQS
UniRef90_G5BBL7_1_440             MGDWSFLGNILEEVNEHSTVIGRVWLTVLFIFRILILGTAAEFVWGDEQS
UniRef90_M3XED1_1_440             MGDWSFLGNILEEVNEHSTVIGRVWLTVLFIFRILILGTAAEFVWGDEQS
UniRef90_L5K685_1_440             MGDWSFLGNILEEVNEHSTVIGRVWLTVLFIFRILILGTAAEFVWGDEQS
UniRef90_F6VG96_1_441             MGDWSFLGNILEEVNEHSTVIGRVWLTVLFIFRILILGTAAEFVWGDEQS
UniRef90_P55917_1_440             MGDWSFLGNILEEVNEHSTVIGRVWLTVLFIFRILILGTAAEFVWGDEQS
UniRef90_UPI000329268E_1_442      MGDWSFLGNILEEVNEHSTVIGRVWLTVLFIFRILILGTAAEFVWGDEQS
UniRef90_G3UBH4_1_410             MGDWSFLGNILEEVNEHSTVIGRVWLTVLFIFRILILGTAAEYVWGDEQS
UniRef90_I3N7I6_1_431             MGDWSFLGNILEEVNEHSTVIGRVWLTVLFIFRILILGTAAEFVWGDEQS
UniRef90_G3X210_1_429             MGDWSFLGNILEEVNEHSTVIGRVWLTVLFIFRILILGTAAEFVWGDEQS
UniRef90_F7BI06_1_372             MGDWSFLGNILEEVNEHSTVIGRVWLTVLFIFRILILGTAAEFVWGDEQS
UniRef90_UPI000333FA9E_1_424      MGDWSFLGNILEEVNEHSTVIGRVWLTVLFIFRILILGTAAEFVWGDEQS
UniRef90_K7EY71_1_406             MGDWSFLGNILEEVNEHSTVIGRVWLTVLFIFRILILGTAAEFVWGDEQS
UniRef90_M7BCA4_1_394             MGDWSFLGNILEEVNEHSTVIGRVWLTVLFIFRILILGTAAEFVWGDEQS
UniRef90_P36381_1_400             MGDWSFLGNILEQVNEQSTVIGRVWLTVLFIFRILILGTAAELVWGDEQS
UniRef90_H2MUH4_1_428             MGDWSFLGNILEEVNEHSTVIGRVWLTVLFIFRILILGTAAEFVWGDEQS
UniRef90_UPI00022B4A0D_1_396      MGDWSFLGNILEEVNEHSTVIGRVWLTVLFIFRILILGTAAEFVWGDEQS
UniRef90_H3D8V1_1_361             MGDWSFLGNILEEVNEHSTVIGRVWLTVLFIFRILILGTAAEFVWGDEQS
UniRef90_G3NHJ7_1_420             MGDWSFLGNILEEVNEHSTVIGRVWLTVLFIFRILILGTAAEFVWGDEQS
UniRef90_Q503J6_1_391             MGDWSFLGNILEEVNEHSTVIGRVWLTVLFIFRILILGTAAEFVWGDEQS
UniRef90_F6QCF2_1_438             MGDWSFLGNILEEVNEHSTVIGRVWLTVLFIFRILILGTAAEFVWGDEQS
UniRef90_UPI00034FAB61_1_453      MGDWSFLGNILEEVNEHSTVIGRVWLTVLFIFRILILGTAAEFVWGDEQS
UniRef90_UPI0001DE8510_1_439      MGDWSFLGNILEEVNEHSTVIGRVWLTVLFIFRILILGTAAEFVWGDEQS
UniRef90_H3A837_1_370             MGDWSFLGNILEEVNEHSTVIGRVWLTVLFIFRILILGTAAEFVWGDEQS
UniRef90_G1KPB7_1_391             MGDWSFLGNILEEVNEHSTVLGRVWLTVLFIFRILILGTAAEFVWGDEQS
UniRef90_B0S6Z4_1_382             MGDWSFLGNILEEVNEHSTVIGRVWLTVLFIFRILILGTAAEFVWGDEQS
UniRef90_G1PZ72_1_426             MGDWSFLGNILEEVSEHSTAVGRVWLTVLFIFRILILGTAAELVWGDEQA
UniRef90_UPI000155FB82_1_448      MGDWSFLGKLLENAQEHSTVIGKVWLTVLFIFRILVLGAAAEEVWGDEQS
UniRef90_F6U7L2_1_428             MGDWSFLGKLLENAQEHSTVIGKVWLTVLFIFRILVLGAAAEEVWGDEQS
UniRef90_UPI0002C6341D_1_440      MGDWSFLGRLLENAQEHSTIIGKVWLTVLFIFRILVLGAAAEEVWGDEQS
UniRef90_M3Z8M0_1_445             MGDWSFLGRLLENAQEHSTVIGKVWLTVLFIFRILVLGAAAEEVWGDEQS
UniRef90_G1Q1M8_1_411             MGDWSFLGRLLENAQEHSTVIGKVWLTVLFIFRILVLGAAVEEVWGDEQS
UniRef90_G1PYG2_1_394             MGDWSLLGRLLDTAQGHSTVIGKVWLTVLFIFRILLLGAGVESVWGDEQS
UniRef90_L5L5Q1_1_430             MGDWSFLGRLLENAQEHSTVIGKVWLTVLFIFRILVLGAGVESVWGDEQS
UniRef90_UPI0002BCF55A_1_410      MGDWSFLGRLLENAQEHSTVVGKVWLTVLFIFRILVLGAAAEEVWGDEQS
UniRef90_P41987_1_407             MGDWSFLGRLLENAQEHSTVIGKVWLTVLFIFRILVLGAAAEEVWGDEQS
UniRef90_J9JHI9_1_433             MGDWSFLGRLLENAQEHSTVIGKVWLTVLFIFRILVLGAAAEEVWGDEQS
UniRef90_Q9Y6H8_1_435             MGDWSFLGRLLENAQEHSTVIGKVWLTVLFIFRILVLGAAAEDVWGDEQS
UniRef90_G3QHB2_1_399             MGDWSFLGRLLENAQEHSTVIGKVWLTVLFIFRILVLGAAAEDVWGDEQS
```

```
UniRef90_F7B1T8_1_432           MGDWSFLGRLLENAQEHSTVIGKVWLTVLFIFRILVLGAAAEDVWGDEQS
UniRef90_L9L2D3_1_460           MGDWSFLGRLLENAQEHSTVVGKVWLTVLFIFRILVLGAAAEEVWGDEQS
UniRef90_Q64448_1_417           MGDWSFLGRLLENAQEHSTVIGKVWLTVLFIFRILVLGAAAEEVWGDEQS
UniRef90_G3H8J5_1_417           MGDWSFLGRLLENAQEHSTVIGKVWLTVLFIFRILVLGAAAEEVWGDEQS
UniRef90_UPI0003333DA4_1_405    MGDWSFLGRLLENAQEHSTVIGKVWLTVLFIFRILVLGAAAEEVWGDEQS
UniRef90_I3NC37_1_417           MGDWSFLGRLLENAQEHSTVIGKVWLTVLFIFRILVLGAAAEEVWGDEQS
UniRef90_H0XD37_1_430           MGDWSFLGRLLENAQEHSTVIGKVWLTVLFIFRILVLGAAAEEVWGDEQS
UniRef90_G1U3S9_1_407           MGDWSFLGRLLENAQEHSTVIGKVWLTVLFIFRILVLGAAAEEVWGDEQS
UniRef90_UPI00032AEFC1_1_413    MGDWSFLGRLLENAQEHSTVIGKVWLTVLFIFRILVLGAAAEEVWGDEQS
UniRef90_UPI00034F30A8_1_402    MGDWSFLGRLLESAQEHSTVIGKVWLTVLFIFRILVLGAAAEEVWGDEQS
UniRef90_UPI0003340A67_1_370    MGDWSFLGRLLENAQEHSTVIGKVWLTVLFIFRILVLGAAAEEVWGDEQS
UniRef90_UPI0002234D0E_1_449    MGDWSFLGRLLENAQEHSTVIGKVWLTVLFIFRILVLGAAAEEVWGDEQS
UniRef90_G3TTN5_1_429           MGDWSFLGRLLENAQEHSTVIGKVWLTVLFIFRILVLGAAAEEVWGDEQS
UniRef90_UPI0002C457ED_1_447    MGDWSFLGRLLENAQEHSTVIGKVWLTVLFIFRILVLGAAAEEVWGDEQS
UniRef90_UPI000333D50E_1_427    MGDWSFLGRLLENAQEHSTVVGKVWLTVLFIFRILVLGAAAEEVWGDEQS
UniRef90_UPI0003343FBC_1_423    MGDWSFLGRLLENAQEHSTVVGKVWLTVLFIFRILVLGAAAEEVWGDEQS
UniRef90_F7G5V2_1_446           MGDWSFLGRLLENAQEHSTVIGKVWLTVLFIFRILVLGAAAEEVWGDEQS
UniRef90_G3VY19_1_428           MGDWSFLGRLLENAQEHSTVIGKVWLTVLFIFRILVLGAAAEEVWGDEQS
UniRef90_UPI000155BBAC_1_459    MGDWSFLGRLLENAQEHSTVIGKVWLTVLFIFRILVLGAAAEEVWGDEQS
UniRef90_F7ETY8_1_425           MGDWSFLGRLLENAQEHSTVIGKVWLTVLFIFRILVLGAAAEEVWGDEQS
UniRef90_I3KXW1_1_474           MGDWSFLGRLLENAQEHSTVIGKVWLTVLFIFRILVLGAAAEEVWGDEQS
UniRef90_UPI00022B3AE8_1_449    MGDWSFLGRLLENAQEHSTVIGKVWLTVLFIFRILVLGAAAEEVWGDEQS
UniRef90_UPI0002A48057_1_447    MGDWSFLGRLLENAQEHSTVIGKVWLTVLFVFRILVLGAATEEVWGDEQS
UniRef90_H2MCD5_1_421           MGDWSFLGRLLENAQEHSTVIGKVWLTVLFVFRILVLGAATEEVWGDEQS
UniRef90_H2UTF3_1_420           MGDWSFLGRLLENAQEHSTVIGKVWLTVLFIFRILVLGAAAEEVWGDEQS
UniRef90_UPI000298F1F6_1_460    MGDWSFLGRLLENAQEHSTVIGKVWLTVLFIFRILVLGAAAEEVWGDEQS
UniRef90_H3DE08_1_376           MGDWIFLGRLLEN-REHSTVIGKVWLTVLFIFRILVLGAAAEEVWGDEQS
UniRef90_Q8JFD2_1_434           MGDWSFLGRLLENAQEHSTVIGKVWLTVLFIFRILVLGAAAEEVWGDEQS
UniRef90_H2T4Y1_1_438           MGDWSFLGRLLENAQEHSTVIGKVWLTVLFIFRILVLGAAAEEVWGDEQS
UniRef90_H2T4Y2_1_428           MGDWSFLGRLLENAQEHSTVIGKVWLTVLFIFRILVLGAAAEEVWGDEQS
UniRef90_H3BZL0_1_378           MGDWSFLGRLLENAQEHSTVIGKVWLTVLFIFRILVLGAAAEEVWGDEQS
UniRef90_I3K1N0_1_478           MGDWSFLGRLLENAQEHSTVIGKVWLTVLFIFRILVLGAAAEEVWGDEQS
UniRef90_H2LFL5_1_470           MGDWSFLGRLLENAQEHSTVIGKVWLTVLFIFRILVLGAAAEEVWGDEQS
UniRef90_M7BWJ7_1_486           MGDWSFLGRLLENAQEHSTVIGKVWLTVLFIFRILVLGAAAEEVWGDEQS
UniRef90_K7F163_1_492           MGDWSFLGRLLENAQEHSTVIGKVWLTVLFIFRILVLGAAAEEVWGDEQS
UniRef90_H0ZM32_1_512           MGDWSFLGRLLENAQEHSTVIGKVWLTVLFIFRILVLGAAAEEVWGDEQS
UniRef90_H3AH16_1_396           MGDWSFLGRLLENAQEHSTVIGKVWLTVLFIFRILVLGAAAEEVWGDEQS
UniRef90_B0BM18_1_388           MGDWSFLGRLLENAQEHSTVIGKVWLTVLFIFRILVLGAAAEEVWGDEQS
UniRef90_Q4S8R0_1_345           MGDWNLLGKLLESAQEHSTVVGKVWLTVLFIFRILVLGTAAEKVWGDEQS
UniRef90_I3KYH7_1_341           MADWNLLGKLLESAQEHSTVVGKVWLTVLFIFRILVLGAAAEKVWGDEQS
UniRef90_H2M0G7_1_345           MGDWNLLGKLLESAQEHSTVVGKVWLTVLFIFRILVLGTAAEKVWGDEQS
UniRef90_H3A471_1_360           MGDWTSLGKLLENAQEHSTVVGKVWLTVLFIFRILVLGTAAEKVWGDEQS
UniRef90_Q6GLA5_1_353           MGDWNLLGKLLESAQEHSTVVGKVWLTVLFIFRILVLGTAAEKVWGDEQS
UniRef90_UPI0002A4A1D5_1_346    MGDWNLLGKLLEKAQEHSTVVGKVWLTVLFIFRILVLSTATDKVWGDEQS
UniRef90_S4RXA6_1_456           MGDWSFLGRLLENAQTHSTVVGKVWLSVLFIFRILVLGAAAESVWGDEQA
UniRef90_P16863_1_360           MGDWSALGRLLDKVQAYSTAGGKVWLSVLFIFRILLLGTAVESAWGDEQS
UniRef90_H2LU51_1_404           MGDWNFLGGILEEVHIHSTMVGKIWLTILFIFRMLVLGVAAEDVWNDEQS
UniRef90_I3KYL3_1_401           MGDWNFLGGILEEVHIHSTMVGKIWLTILFIFRMLVLGVAAEDVWNDEQS
UniRef90_G3NC44_1_402           MGDWNFLGGILEEVHIHSTMVGKIWLTILFIFRMLVLGVAAEDVWNDEQS
UniRef90_H2SSL2_1_392           MGDWNFLGGILEEVHIHSTMVGKIWLTILFIFRMLVLGVAAEDVWNDEQS
UniRef90_UPI0002C46B7E_1_433    MGDWNFLGGILEEVHIHSTIIGKIWLTILFIFRMLVLGVAAEDVWNDEQS
UniRef90_G3ULF1_1_432           MGDWNFLGGILEEVHIHSTMIGKIWLTILFIFRMLVLGVAAEDVWNDEQS
UniRef90_C9IYK1_1_407           MGDWNLLGDTLEEVHIHSTMIGKIWLTILFIFRMLVLGVAAEDVWNDEQS
UniRef90_H0Y0G7_1_436           MGDWNLLGDTLEEVHIHSTMIGKIWLTILFIFRMLVLGVAAEDVWNDEQS
UniRef90_L9JD22_1_408           MGDWNLLGGILEEVHIHSTMIGKIWLTILFIFRMLVLGVAAEDVWNDEQS
UniRef90_F1N617_1_436           MGDWNFLGGILEEVHIHSTVIGKMWLTILFIFRMLVLGVAAEDVWNDEQS
UniRef90_F1SV25_1_385           MGDWNFLGGILEEVHIHSTMIGKIWLTILFIFRMLVLGVAAEDVWNDEQS
UniRef90_L5JUH5_1_307           MGDWNFLGGILEEVHTHSTMIGKIWLTILFIFRMLVLDVAAENVWNDEQS
UniRef90_UPI00020369CA_1_441    MGDWNFLGGILEEVHIHSTIIGKIWLTILFVFRMLVLGVATEDVWNDEQS
UniRef90_R4GLX1_1_405           MGDWNFLGGILEEVHIHSTIIGKIWLTILFVFRMLVLGVATEDVWNDEQS
UniRef90_UPI00035A19E6_1_466    MGDWNFLGGILEEVHIHSTIIGKIWLTILFVFRMLVLGVATEDVWNDEQS
UniRef90_F6WA84_1_463           MGDWNFLGGILEEVHIHSTIIGKIWLTILFIFRMLVLGVAAEDVWNDEQS
UniRef90_G1SG18_1_430           MGDWNLLGGILEEVHYHSTIVGKIWLTILFIFRMLVLGVAAEDVWDDEQS
UniRef90_UPI00032B0E64_1_413    MGDWNLLGGILEEVHSHSTIVGKIWLTILFIFRMLVLGVAAEDVWDDEQS
UniRef90_UPI0002C65D9D_1_412    MGDWNLLGGILEEVHSHSTIVGKIWLTILFIFRMLVLGVAAEDVWDDEQS
UniRef90_F7CF28_1_383           MGDWNLLGGILEEVHSHSTIVGKIWLTILFIFRMLVLGVAAEDVWDDEQS
UniRef90_Q969M2_1_435           MGDWNLLGGILEEVHSHSTIVGKIWLTILFIFRMLVLRVAAEDVWDDEQS
UniRef90_L9JVZ3_1_406           MGDWNLLGGILEEVHSHSTIVGKIWLTILFIFRMLVLGVAAEDVWDDEQS
UniRef90_I3M407_1_414           MGDWNLLGGILEEVHSHSTIVGKIWLTILFIFRMLVLGVAAEDVWDDEQS
UniRef90_L5JMB7_1_435           MGDWNLLGGILEEVHSHSTIVGKIWLTILFIFRMLVLGAAAEDVWYDEQS
UniRef90_L8J083_1_417           MGDWNLLGGILEDVHSHSTMVGKIWLTILFIFRMLILGVAAEDVWDDEQS
UniRef90_UPI0002BCCFF6_1_421    MGDWNLLGGILEEAHSHSTMVGKIWLTILFVFRMLILGVAAEDVWDDEQS
UniRef90_F1RY20_1_421           MGDWNLLGGVLEEVHSHSTIVGKIWLTILFIFRMLILGVAAEDVWDDEQS
UniRef90_UPI0002C59166_1_412    MGDWNLLGGILEEVHSHSTIVGKIWLTILFIFRMLVLGVAAEDVWDDEQS
UniRef90_G3TBX7_1_436           MGDWNLLGGILEEVHSHSTIVGKIWLTILFIFRMLVLGVAAEGVWDDEQS
UniRef90_G3GSS7_1_405           MGDWNLLGGILEEVHSHSTIVGKIWLTILFIFRMLVLGVAAEDVWDDEQS
```

```
UniRef90_UPI0003316158_1_413      MGDWNLLGGILEEVHSHSTIVGKIWLTILFIFRMLVLGVAAEDVWDDEQS
UniRef90_G3WUN4_1_443             MGDWNLLGSILEEVHFHSTLVGKIWLTILFIFRMLVLGVAAEDVWDDEQS
UniRef90_F6W379_1_264             MGDWNLLGSILEEVHIHSTIVGKIWLTILFVFRMLVLGVAAEDVWDDEQD
UniRef90_M7BL54_1_431             MGDWNLLGSILEEVHIHSTIVGKIWLTILFIFRMLVLGVAAEDVWDDEQS
UniRef90_UPI0002B44362_1_276      MGDWNLLGSILEEVHIHSTIVGKIWLTILFIFRMLVLGVAAEDVWDDEQS
UniRef90_UPI000298933C_1_429      MGDWNLLGSILEEVHIHSTIVGKIWLTILFIFRMLVLGVAAEDVWDDEQS
UniRef90_H3CDZ2_1_426             MGDWNLLGSILEEVHIHSTIVGKIWLTILFIFRMLVLGVAAEDVWDDEQS
UniRef90_I3IZH7_1_440             MGDWNLLGSILEEVHIHSTIVGKIWLTILFIFRMLVLGVAAEDVWDDEQS
UniRef90_E7F3P4_1_389             MGDWNLLGSILEEVHIHSTIVGKIWLTILFIFRMLVLGVAAEDVWDDEQS
                                  * :*  *.  *:      **  *::**::**:**:*:* ...   * **:

UniRef90_UPI0002C691BE_1_358      DFQCDTIQPGCENVCYDQAFPV-AHIRYWVLQIIFVSTPSLLYTLHAMHT
UniRef90_F7BKC0_1_358             DFLCDTLQPGCENVCYDQAFPI-SHIRYWVLQVIFVSTPSLVYLFHTMHR
UniRef90_Q0VCR2_1_359             DFLCDTMQPGCENVCYDQAFPI-SHIRYWVLQVIFVSTPSLVYLGHAVHM
UniRef90_G3TUX5_1_358             DFQCDTLQPGCENVCYDQAFPI-SHIRYWVLQIIFVSTPSLVYMGHAMHM
UniRef90_L9JKN2_1_358             DFQCDTMQPGCENVCYDQAFPI-SHIRYWVLQIIFVSTPSLLYMGHAMHT
UniRef90_P36382_1_358             DFRCDTIQPGCQNVCYDQAFPI-SHIRYWVLQIIFVSTPSLVYMGHAMHT
UniRef90_UPI0003315E33_1_350      DFQCDTMQPGCENVCYDQAFPI-SHIRFWVLQIIFVSTPSLLYMGHAMHT
UniRef90_UPI000333FB64_1_358      DFQCDTMQPGCENVCYDQAFPI-SHIRYWVLQIIFVSTPSLVYMGHAMHT
UniRef90_UPI000334540D_1_357      DFQCDTIQPGCENVCYDQAFPI-SHIRFWVLQIIFVSTPSLLYMGHAMHT
UniRef90_L5K3R8_1_358             DFQCDTIQPGCENVCYDQAFPI-SHIRYWVLQIIFVSTPSLVYMGHAVHH
UniRef90_G1TQ37_1_358             DFQCDTIQPGCENVCYDQAFPI-SHIRYWVLQIIFVSTPSLVYMGHAMHT
UniRef90_UPI0001776F40_1_352      DFQCDTIQPGCENVCYDQAFPI-SHIRFWVLQIIFVSTPSLVYMGHAMHT
UniRef90_G3H2Q9_1_358             DFRCDTIQPGCQNVCYDQAFPI-SHIRYWVLQIIFVSTPSLVYMGHAMHT
UniRef90_Q01231_1_358             DFRCDTIQPGCQNVCYDQAFPI-SHIRYWVLQIIFVSTPSLVYMGHAMHT
UniRef90_G5BBL6_1_357             DFQCDTIQPGCENVCYDQAFPI-SHIRYWVLQIIFVSTPSLVYMGHAMHT
UniRef90_H0WCZ7_1_358             DFQCDTMQPGCENVCYDQAFPI-SHIRYWVLQIIFVSTPSLVYMGHAMHT
UniRef90_G3X1A3_1_358             DFQCDTLQPGCENVCYDQAFPI-SHIRYWVLQIIFVSTPSLLYMGHAMHT
UniRef90_H0ZTI1_1_370             DFMCDTQQPGCENVCYDKAFPI-SHVRFWVLQIIFVSTPSLVYMGHAMHT
UniRef90_P18860_1_369             DFMCDTQQPGCENVCYDKAFPI-SHVRFWVLQIIFVSTPSLVYMGHAMHT
UniRef90_G1KPB5_1_370             DFMCDSRQPGCENVCYDQAFPI-SHVRYWVLQIIFVSTPSLLYMGHAMHT
UniRef90_K7FWT6_1_369             DFMCDTRQPGCENVCYDKAFPI-SHIRYWVLQVIFVSTPSLVYMGHAMHT
UniRef90_F1QL21_1_400             DFMCDTLQPGCTNVCYDRAFPI-AHIRYWVLQIVFVSTPSLIYMGHAMHT
UniRef90_UPI0002A497DC_1_408      DFNCDTLQPGCENVCYDEAFPI-AHIRFWVLQIVFVSTPSLIYVGHAMHI
Input_protein_seq                 DFVCNTQQPGCENVCYDEAFPI-SHIRLWVLQIIFVSTPSLMYVGHAVHY
UniRef90_UPI00027FC10C_1_397      DFVCNTQQPGCENVCYDEAFPI-SHTRLWVLQIILVSTPSLMYVGHAVHH
UniRef90_L9JGQ0_1_441             DFVCNTQQPGCENVCYDEAFPI-SHIRLWVLQIIFVSTPSLMYVGHAVHH
UniRef90_P28236_1_440             DFVCNTQQPGCENVCYDEAFPI-SHIRLWVLQIIFVSTPSLMYVGHAVHH
UniRef90_G5BBL7_1_440             DFVCNTQQPGCENVCYDEAFPI-SHIRLWVLQIIFVSTPSLMYVGHAVHY
UniRef90_M3XED1_1_440             DFVCNTQQPGCENVCYDEAFPI-SHIRLWVLQIIFVSTPSLVYVGHAVHH
UniRef90_L5K685_1_440             DFVCNTQQPGCENVCYDEAFPI-SHIRLWVLQIIFVSTPSLVYVGHAVHH
UniRef90_F6VG96_1_441             DFVCNTQQPGCENVCYDEAFPI-SHIRLWVLQIIFVSTPSLVYVGHAVHH
UniRef90_P55917_1_440             DFVCNTQQPGCENVCYDEAFPI-SHIRLWVLQIIFVSTPSLVYVGHAVHH
UniRef90_UPI000329268E_1_442      DFVCNTQQPGCENVCYDEAFPI-SHIRLWVLQIIFISTPSLVYVGHAMHH
UniRef90_G3UBH4_1_410             DFVCNTQQPGCENVCYDEAFPI-SHIRLWVLQIIFVSTPSLVYVGHAVHY
UniRef90_I3N7I6_1_431             DFVCNTQQPGCENVCYDEAFPI-SHIRLWVLQIIFVSTPSLMYVGHAVHH
UniRef90_G3X210_1_429             DFVCNTQQPGCENVCYDEAFPI-SHIRLWVLQIIFVSTPSLVYVGHAVHH
UniRef90_F7BI06_1_372             DFVCNTQQPGCENVCYDEAFPI-SHIRLWVLQIIFVSTPSLVYVGHAVHH
UniRef90_UPI000333FA9E_1_424      DFVCNTQQPGCENVCYDQAFPI-SHIRLWVLQIIFVSTPSLVYVGHAMHH
UniRef90_K7EY71_1_406             DFVCNTQQPGCENVCYDEAFPI-SHIRLWVLQIIFVSTPSLVYVGHAVHH
UniRef90_M7BCA4_1_394             DFVCNTQQPGCENVCYDEAFPI-SHIRLWVLQIIFVSTPSLVYVGYAVHH
UniRef90_P36381_1_400             DFVCNTQQPGCENVCYDEAFPI-SHIRLWVLQIIFVSTPSLVYFGHAVHH
UniRef90_H2MUH4_1_428             DYVCNTQQPGCENVCYDEAFPI-SHIRLWVLQIIFVSTPSLVYVGHAVHH
UniRef90_UPI00022B4A0D_1_396      DYVCNTQQPGCENVCYDEAFPI-SHIRLWVLQIIFVSTPSLVYVGHAVHH
UniRef90_H3D8V1_1_361             DYVCNTQQPGCENVCYDEAFPI-SHIRLWVLQIIFVSTPSLVYVGHAVHH
UniRef90_G3NHJ7_1_420             DYVCNTQQPGCENVCYDEAFPI-SHIRLWVLQIIFVSTPSLVYVGHAVHH
UniRef90_Q503J6_1_391             DYVCNTQQPGCENVCYDEAFPI-SHIRLWVLQIIFVSTPSLVYVGHAVHH
UniRef90_F6QCF2_1_438             DFVCNTQQPGVK-CLYDEGFS--STLRLWVLQIIFS-TPSLVYVGHAVHH
UniRef90_UPI00034FAB61_1_453      DFVCNTQQPGCENVCYDEAFPL-SHIRLWVLQIIFVSTPSLVYVGHAVHH
UniRef90_UPI0001DE8510_1_439      DFVCNTQQPGVK-CLYDEGFSSLPHXGCGFSRLSFGSTPSLVYVGHAVHH
UniRef90_H3A837_1_370             DFVCNTQQPGCENVCYDEAFPL-SHIRLWVLQIIFVSTPSLVYVGHAVHH
UniRef90_G1KPB7_1_391             DFVCNTQQPGCENVCYDEAFPI-SHIRLWVLQIIFVSTPSLIYLGHTAHH
UniRef90_B0S6Z4_1_382             DYVCNTQQPGCENVCYDEAFPI-SHIRLWVLQIIFVSTPSLVYVGHAVHY
UniRef90_G1PZ72_1_426             DFVCNTQQPGCENVCYDAAFPI-SHIRLWVLQIIFVSAPSLVYVGHAVHR
UniRef90_UPI000155FB82_1_448      DFTCNTQQPGCENVCYDRAFPI-SHVRFWVLQIIFVSTPTLIYLGHVLHL
UniRef90_F6U7L2_1_428             DFTCNTQQPGCENVCYDRAFPI-SHVRFWVLQIIFVSTPTLIYLGHVLHL
UniRef90_UPI0002C6341D_1_440      DFTCNTQQPGCENVCYDRAFPI-SHIRFWVLQIIFVSTPTLIYLGHVLHI
UniRef90_M3Z8M0_1_445             DFMCNTQQPGCENVCYDKAFPI-SHIRFWVLQIIFVSTPTLIYLGHVLHI
UniRef90_G1Q1M8_1_411             DFTCNTQQPGCENVCYDKAFPI-SHTRFWVLQIIFVSTPTLIYLGHVLHI
UniRef90_G1PYG2_1_394             GFTCNTQQPGCENVCYDKAFPI-SHTRFWVLQIIFVSTPTLIYLGHVLHM
UniRef90_L5L5Q1_1_430             DFTCNTQQPGCENVCYDKAFPI-SHTRFWVLQIIFVSTPTLIYLGHVLHI
UniRef90_UPI0002BCF55A_1_410      DFTCNTQQPGCENVCYDRAFPI-SHVRFWVLQIIFVSTPTLIYLGHVLHL
UniRef90_P41987_1_407             DFTCNTQQPGCENVCYDRAFPI-SHVRFWVLQIIFVSTPTLIYLGHVLHL
UniRef90_J9JHI9_1_433             DFTCNTQQPGCENVCYDRAFPI-SHIRFWVLQIIFVSTPTLIYLGHVLHI
UniRef90_Q9Y6H8_1_435             DFTCNTQQPGCENVCYDRAFPI-SHIRFWALQIIFVSTPTLIYLGHVLHI
UniRef90_G3QHB2_1_399             DFTCNTQQPGCENVCYDRAFPI-SHIRFWALQIIFVSTPTLIYLGHVLHI
```

UniRef90_F7B1T8_1_432	DFTCNTQQPGCENVCYDRAFPI-SHIRFWALQIIFVSTPTLIYLGHVLHM
UniRef90_L9L2D3_1_460	DFTCNTQQPGCENVCYDRAFPI-SHTRFWALQIIFVSTPTLIYLGHVLHI
UniRef90_Q64448_1_417	DFTCNTQQPGCENVCYDRAFPI-SHIRFWALQIIFVSTPTLIYLGHVLHI
UniRef90_G3H8J5_1_417	DFTCNTQQPGCENVCYDRAFPI-SHIRFWALQIIFVSTPTLIYLGHVLHI
UniRef90_UPI0003333DA4_1_405	DFTCNTQQPGCENVCYDRAFPI-SHIRFWALQIIFVSTPTLIYLGHVLHI
UniRef90_I3NC37_1_417	DFTCNTQQPGCENVCYDRAFPI-SHIRFWVLQIIFVSTPTLIYLGHVLHI
UniRef90_H0XD37_1_430	DFTCNTQQPGCENVCYDKAFPI-SHIRFWALQIIFVSTPTLIYLGHVLHI
UniRef90_G1U3S9_1_407	DFTCNTQQPGCENVCYDRAFPI-SHIRFWALQIIFVSTPTLIYLGHVLHI
UniRef90_UPI00032AEFC1_1_413	DFTCNTQQPGCENVCYDRAFPI-SHIRFWALQIIFVSTPTLIYLGHVLHI
UniRef90_UPI00034F30A8_1_402	DFTCNTQQPGCRNVCYDSAFPI-SHIRFWALQIIFVSTPTLIYLGHVLHL
UniRef90_UPI0003340A67_1_370	DFTCNTQQPGCENVCYDRAFPI-SHIRFWALQIIFVSTPTLIYLGHVLHL
UniRef90_UPI0002234D0E_1_449	DFTCNTQQPGCENVCYDQAFPI-SHIRFWVLQIIFVSTPTLIYLGHVLHI
UniRef90_G3TTN5_1_429	DFTCNTQQPGCENVCYDQAFPI-SHIRFWVLQIIFVSTPTLIYLGHVLHI
UniRef90_UPI0002C457ED_1_447	DFTCNTQQPGCENVCYDQAFPI-SHIRFWVLQIIFVSTPTLIYLGHVLHL
UniRef90_UPI000333D50E_1_427	DFTCNTQQPGCENVCYDQAFPI-SHIRFWVLQIIFVSTPTLIYLGHVLHI
UniRef90_UPI0003343FBC_1_423	DFTCNTQQPGCENVCYDQAFPI-SHIRFWVLQIIFVSTPTLIYLGHVLHI
UniRef90_F7G5V2_1_446	DFTCNTQQPGCENVCYDKAFPI-SHIRFWVLQIIFVSTPTLIYLGHVLHI
UniRef90_G3VY19_1_428	DFTCNTQQPGCENVCYDKAFPI-SHIRFWVLQIIFVSTPTLIYLGHVLHI
UniRef90_UPI000155BBAC_1_459	DFTCNTQQPGCENVCYDEAFPI-SHIRFWVLQIIFVSTPSLIYLGHVLHI
UniRef90_F7ETY8_1_425	DFTCNTQQPGCENVCYDEAFPI-SHIRFWVLQIIFVSTPSLIYLGHVLHI
UniRef90_I3KXW1_1_474	DFTCNTQQPGCENVCYDEAFPI-SHIRFWVLQIIFVSTPTLIYLGHVLHI
UniRef90_UPI00022B3AE8_1_449	DFTCNTQQPGCENVCYDEAFPI-SHIRFWVLQIIFVSTPTLIYLGHVLHI
UniRef90_UPI0002A48057_1_447	DFTCNTQQPGCENVCYDEAFPI-SHIRFWVLQIIFVSTPTLIYLGHVLHI
UniRef90_H2MCD5_1_421	DFTCNTQQPGCENVCYDEAFPI-SHIRFWVLQIIFVSTPTLIYLGHVLHI
UniRef90_H2UTF3_1_420	DFTCNTQQPGCENVCYDEAFPI-SHIRFWVLQIIFVSTPTLIYLGHVLHI
UniRef90_UPI000298F1F6_1_460	DFTCNTQQPGCENVCYDEAFPI-SHIRFWVLQIIFVSTPTLIYLGHVLHI
UniRef90_H3DE08_1_376	DFTCNTQQPGCENVCYDEAFPI-SHIRFWVLQIIFVSTPTLIYLGHVLHI
UniRef90_Q8JFD2_1_434	DFTCNTQQPGCENVCYDEAFPI-SHIRFWVLQIIFVSTPTLIYLGHVLHI
UniRef90_H2T4Y1_1_438	DFTCNTQQPGCENVCYDEAFPI-SHIRFWVLQIIFVSTPTLIYLGHVLHI
UniRef90_H2T4Y2_1_428	DFTCNTQQPGCENVCYDEAFPI-SHIRFWVLQIIFVSTPTLIYLGHVLHI
UniRef90_H3BZL0_1_378	DFTCNTQQPGCENVCYDEAFPI-SHIRFWVLQIIFVSTPTLIYLGHVLHI
UniRef90_I3K1N0_1_478	DFTCNTQQPGCENVCYDEAFPI-SHIRFWVLQIIFVSTPTLIYLGHVLHI
UniRef90_H2LFL5_1_470	DFTCNTQQPGCENVCYDEAFPI-SHIRFWVLQIIFVSTPTLIYLGHVLHI
UniRef90_M7BWJ7_1_486	DFICNTQQPGCENVCYDKAFPI-SHIRFWVLQIIFVSTPTLIYLGHVLHI
UniRef90_K7F163_1_492	DFTCNTQQPGCENVCYDQAFPI-SHIRFWVLQIIFVSTPTLIYLGHVLHI
UniRef90_H0ZM32_1_512	DFTCNTQQPGCENVCYDKAFPI-SHIRFWVLQIIFVSTPTLIYLGHVLHI
UniRef90_H3AH16_1_396	DFTCNTQQPGCENVCYDKAFPI-SHIRFWVLQIIFVSTPTLIYLGHVLHI
UniRef90_B0BM18_1_388	DFTCNTQQPGCENVCYDKAFPI-SHIRFWVLQIIFVSTPTLIYLGHVLHI
UniRef90_Q4S8R0_1_345	GFTCDTKQPGCQNVCYDKTFPI-SHIRFWVMQIIFVSTPTLIYLGHILHL
UniRef90_I3KYH7_1_341	SFTCDTKQPGCQNVCYDTTFPI-SHIRFWVMQIIFVSTPTLIYLGHILHL
UniRef90_H2M0G7_1_345	GFTCDTKQPGCQNVCYDKTFPI-SHIRFWVMQIIFVSTPTLVYLGHILHL
UniRef90_H3A471_1_360	GFTCDTKQPGCQNVCYDWTFPI-SHIRFWVLQIIFVSTPTLIYLGHILHI
UniRef90_Q6GLA5_1_353	GFTCDTKQPGCQNVCYDHTFPI-SHIRFWVLQIIFVSTPTLIYLGHILHL
UniRef90_UPI0002A4A1D5_1_346	GFVCDTKQPGCENVCYDITFPI-SHVRFWVLQIIFVSTPTLIYLGHIVHL
UniRef90_S4RXA6_1_456	GFRCNTQQPGCENVCYDEAFPI-SHIRFWVLQIIFVSTPALVYLGHVLHI
UniRef90_P16863_1_360	AFVCNTQQPGCENVCYDKSFPI-SHVRFWVLQIIFVSTPTLLYLAHVFYL
UniRef90_H2LU51_1_404	DFICNTEQPGCRNVCYDQAFPI-SLIRYWVLQVIFVSSPSLVYMGHAIYQ
UniRef90_I3KYL3_1_401	DFICNTEQPGCRNVCYDQAFPI-SLIRYWVLQVIFVSSPSLVYMGHAIYQ
UniRef90_G3NC44_1_402	DFVCNTDQPGCRNVCYDQAFPI-SLIRYWVLQVIFVSSPSLVYMGHAIYQ
UniRef90_H2SSL2_1_392	DFICNTDQPGCRNVCYDQAFPI-SLIRYWVLQVIFVSSPSLVYMGHAIYQ
UniRef90_UPI0002C46B7E_1_433	GFICNTEQPGCRNVCYDQAFPI-SLIRYWVLQVIFVSSPSLVYMGHALYR
UniRef90_G3ULF1_1_432	GFICNTEQPGCRNVCYDQAFPI-SLIRYWVLQVIFVSSPSLVYMGHALYR
UniRef90_C9IYK1_1_407	GFICNTEQPGCRNVCYDQAFPI-SLIRYWVLQVIFVSSPSLVYMGHALYR
UniRef90_H0Y0G7_1_436	GFICNTEQPGCRNVCYDQAFPI-SLIRYWVLQVIFVSSPSLVYMGHALYR
UniRef90_L9JD22_1_408	GFICNTEQPGCRNVCYDQAFPI-SLIRYWVLQVIFVSSPSLVYMGHALYR
UniRef90_F1N617_1_436	GFICNTEQPGCRNVCYDRAFPI-SLIRYWVLQVIFVSSPSLVYMGHALYR
UniRef90_F1SV25_1_385	GFICNTEQPGCRNVCYDQAFPI-SLIRYWVLQVIFVSSPSLVYMGHALYR
UniRef90_L5JUH5_1_307	GFICNTEQPGCRNVCYDQAFPI-SLIRYWVLQVIFVSSPSLVYMGHALYQ
UniRef90_UPI00020369CA_1_441	EFICNTEQPGCRNVCYDEAFPI-SLIRYWVLQVIFVSSPSLVYMGHALYR
UniRef90_R4GLX1_1_405	EFICNTEQPGCRNVCYDEAFPI-SLIRYWVLQVIFVSSPSLVYMGHALYR
UniRef90_UPI00035A19E6_1_466	EFICNTEQPGCRNVCYDEAFPI-SLIRYWVLQVIFVSSPSLVYMGHALYR
UniRef90_F6WA84_1_463	GFVCNTEQPGCRNVCYDRAFPI-SLIRYWVLQVIFVSSPSLVYMGHALYR
UniRef90_G1SG18_1_430	AFTCNTQQPGCNNICYDDAFPI-SLIRFWVLQIIFVSSPSLVYMGHALYR
UniRef90_UPI00032B0E64_1_413	AFACNTQQPGCNNICYDDAFPI-SLIRFWVLQIIFVSSPSLVYMGHALYR
UniRef90_UPI0002C65D9D_1_412	AFACNTQQPGCNNICYDDAFPI-SLIRFWVLQIIFVSSPSLVYMGHALYR
UniRef90_F7CF28_1_383	AFACNTQQPGCNNICYDDAFPI-SLIRFWVLQIIFVSSPSLVYMGHALYR
UniRef90_Q969M2_1_435	AFACNTRQPGCNNICYDDAFPI-SLIRFWVLQIIFVSSPSLVYMGHALYR
UniRef90_L9JVZ3_1_406	AFACNTQQPGCKNICYDDAFPI-SLIRFWVLQIIFVSSPSLVYMGHALYR
UniRef90_I3M407_1_414	AFACNTQQPGCNNICYDDAFPI-SLIRFWVLQIIFVSSPSLVYMGHALYR
UniRef90_L5JMB7_1_435	AFACNTQQPGCNNICYDDAFPI-SLIRFWVLQIIFVSSPSLVYMGHALYR
UniRef90_L8J083_1_417	AFACNTQQPGCNTVCYDTAFPI-SLIRFWVLQIIFVSSPSLVYMGHALYR
UniRef90_UPI0002BCCFF6_1_421	AFACNTQQPGCNTVCYDDAFPI-SLVRFWVLQIIFVSSPSLVYMGHALYS
UniRef90_F1RY20_1_421	AFACNTQQPGCNTVCYDDAFPI-SLIRFWVLQIIFVSSPSLVYMGHALYR
UniRef90_UPI0002C59166_1_412	AFACNTQQPGCNNICYDAAFPI-SLIRFWVLQIIFVSSPSLVYMGHVLYR
UniRef90_G3TBX7_1_436	AFACNTQQPGCNNICYDDAFPI-SLIRFWVLQIIFVSSPSLVYMGHALYR
UniRef90_G3GSS7_1_405	SFACNTQQPGCNNICYDDAFPI-SLIRFWVLQIIFVSSPSLVYMGHALYR

```
UniRef90_UPI0003316158_1_413      AFVCNTQQPGCSNVCYDDAFPI-SLIRFWVLQIIFVSSPSLVYMGHALYR
UniRef90_G3WUN4_1_443             AFTCNTRQPGCRNICYDGAFPI-SLIRYWVLQVIFVSSPSLVYMGHALYR
UniRef90_F6W379_1_264             EFICNTEQPGCRNVCYDQAFPI-SLIRYWVLQIIFVSSPSLVYMGHALYR
UniRef90_M7BL54_1_431             EFICNTEQPGCSNVCYDKAFPI-SLIRYWVLQIIFVSSPSLVYMGHALYR
UniRef90_UPI0002B44362_1_276      EFICNTEQPGCNNICYDKAFPI-SLIRYWVLQIIFVSSPSLVYMGHALYR
UniRef90_UPI000298933C_1_429      EFVCNTEQPGCKNVCYDQAFPV-SLIRYWVLQIIFVSSPSLVYMGHALYR
UniRef90_H3CDZ2_1_426             EFVCNTEQPGCKNVCYDQAFPV-SLIRYWVLQIIFVSSPSLVYMGHALYR
UniRef90_I3IZH7_1_440             EFVCNTEQPGCKTVCYDQAFPI-SLIRYWVLQIIFVSSPSLVYMGHALYR
UniRef90_E7F3P4_1_389             EFVCNTEQPGCKNVCYDQAFPI-SLIRYWVLQIIFVSSPSLVYMGHALYR
                                   : *:: ***     **  *.  .       :: :  :*:*:*  :  :

UniRef90_UPI0002C691BE_1_358      VRMQKKRKL-REAER----AKDVRGAGS---------------YEYPVAE
UniRef90_F7BKC0_1_358             VRMQEKQRQ-REAER----AKHVQGAGS---------------YEYPVAE
UniRef90_Q0VCR2_1_359             VRVQEKRKLLREAER----AKEARAAGS---------------YEYPVAE
UniRef90_G3TUX5_1_358             VRMQEKRKL-REAER----AKGVQGSGS---------------YEYPVAE
UniRef90_L9JKN2_1_358             VRMQEKRKL-REAER----AKEVRGAGS---------------YEYPVVE
UniRef90_P36382_1_358             VRMQEKRKL-REAER----AKEVRGSGS---------------YEYPVAE
UniRef90_UPI0003315E33_1_350      VRMQEKRRL-REAER----AKEVQGAG----------------YEYPAAE
UniRef90_UPI000333FB64_1_358      MRMQEKRRL-RAAER----AKEVRGAGS---------------YEYPVAE
UniRef90_UPI000334540D_1_357      VRVQEKRRL-REAER----ARDVRAAG----------------YEYPAAE
UniRef90_L5K3R8_1_358             VRMEEKRKL-REAER----AREARGAGP---------------CEYPVGE
UniRef90_G1TQ37_1_358             VRMQEKRKM-REAET----GKEVQGAGS---------------YEYPAAE
UniRef90_UPI0001776F40_1_352      VRMQEKRKL-RETQG----TKEGRGAGS---------------YEYPAAE
UniRef90_G3H2Q9_1_358             VRMQEKQKL-RDAEK----AKEAGATGS---------------YEYPVAE
UniRef90_Q01231_1_358             VRMQEKQKL-RDAEK----AKEAHRTGA---------------YEYPVAE
UniRef90_G5BBL6_1_357             VRMQEKRRL-REAER----AKEIHRPGS---------------YEYQVAE
UniRef90_H0WCZ7_1_358             VRMQEKRKL-REAER----AKEIHRPGSP--------------YEFQVAE
UniRef90_G3X1A3_1_358             VRMEEKRKL-KEAEK----SKESKGADT---------------YAYQT-E
UniRef90_H0ZTI1_1_370             VRMEEKRKMKEAEIE----ARENKNGGDTY-----------YQQKCSVAE
UniRef90_P18860_1_369             VRMEEKRKMKEAERE----AQEMKNSGDTY-----------YQQKCPVAE
UniRef90_G1KPB5_1_370             VRMEEKRKLKEAKEG----SKAIQSKSDVF-----------HQQEYPPPE
UniRef90_K7FWT6_1_369             VRIEEKRKIKEAEMR----AKGIRSSGDSY-----------HQQEYPAAE
UniRef90_F1QL21_1_400             VRMEEKRKQK---------EQEEKAEAGK--------------GEKEYLE
UniRef90_UPI0002A497DC_1_408      VRREEKRRSRQEDDRDGGGEEEDQGGGGK--------------VERKGEK
Input_protein_seq                 VRMEEKRKSREAEEL----GQQAGTNG--------------G------PD
UniRef90_UPI00027FC10C_1_397      VRKEEKRRGR----------------------------------------
UniRef90_L9JGQ0_1_441             VRMEEKRKDREAEEL----CQQSAANG--------------GDRAPLTPD
UniRef90_P28236_1_440             VRMEEKRKDREAEEL----CQQSRSNG--------------GERVPIAPD
UniRef90_G5BBL7_1_440             VRMEEKRKDREAEEV----SQQSRSNG--------------CERGPLAPD
UniRef90_M3XED1_1_440             VRMQEKRKEREADEL----CQQGAAEG--------------GDRAPLAAD
UniRef90_L5K685_1_440             VRMEEKRKEREAEEL----CQQAAGDS--------------GERGPLPAD
UniRef90_F6VG96_1_441             VRMEEKRKEREAEEL----GQQPPGN---------------GERAPPAAD
UniRef90_P55917_1_440             VRMEEKRKEREAEEL----SQQSPGNG--------------GERAPLAAD
UniRef90_UPI000329268E_1_442      VRMEEKRKEREAEEL----CQQSGGNG--------------GDRAPLPPD
UniRef90_G3UBH4_1_410             LRMEEKRKEREAEEL----CQQPGGNG--------------PEKGPVAPD
UniRef90_I3N7I6_1_431             VRMQEKRREREAEEP----PAEAER-----------------------AD
UniRef90_G3X210_1_429             VRMEEKRKEREAEEV----CQQSGSN---------------GERLPLALD
UniRef90_F7BI06_1_372             VRMEEKRKEREAEEV----CQQSGGN---------------GERLPLALD
UniRef90_UPI000333FA9E_1_424      VRMQEKRKEREVEEL----CPQPGGNG--------------AERGPLGPD
UniRef90_K7EY71_1_406             VRMVEKRREREQAER----QQQAEMD---------------EERLPLTPD
UniRef90_M7BCA4_1_394             VRMAEKKREREQAER----QQQAEMD---------------EERLPLAPD
UniRef90_P36381_1_400             VRMEEKRKEREEAER----RQQAEVD---------------EEKLPLAPN
UniRef90_H2MUH4_1_428             VHMEEKRKEREEAEL----SRQQELS---------------EERLPLAPD
UniRef90_UPI00022B4A0D_1_396      VHMEEKRKEREEAEL----SRQQELS---------------EERLPLAPD
UniRef90_H3D8V1_1_361             VHMEEKRKEREEAEL----SRQQELS---------------EERLPLAPD
UniRef90_G3NHJ7_1_420             VHMEEKRKEREEAEL----SRQQELS---------------EERLPLAPD
UniRef90_Q503J6_1_391             VHMEEKRKEREEAEL----NRQQEN----------------EERLPLAPD
UniRef90_F6QCF2_1_438             VRMKENWKEREEAEM----SRQQEMN---------------EERLPLAPD
UniRef90_UPI00034FAB61_1_453      VRMEEKRKEREEAEM----SRQQEMN---------------EERLPLAPD
UniRef90_UPI0001DE8510_1_439      VRMKENWKEREEAEM----SRQQEMN---------------EERLPLAPD
UniRef90_H3A837_1_370             VRMEEKRKEREEAEI----SRQQELN---------------EERLPLAPD
UniRef90_G1KPB7_1_391             VRMEEKKRAREEAER----ALQQQDERE-------------DEKSSLDPE
UniRef90_B0S6Z4_1_382             IHMEEKRKEREEAEV----SHQQELCE-----------------ERQAMD
UniRef90_G1PZ72_1_426             ARAEEKRASRVAWVW----LGGGIWLS-----------------------
UniRef90_UPI000155FB82_1_448      VRMEEKKKEREEEPL----KGDNLHHEPA------------GPCGLSSDS
UniRef90_F6U7L2_1_428             VRMEEKKKEREEEPL----KGDNLHHEPA------------GPCGLSSDS
UniRef90_UPI0002C6341D_1_440      VRMEEKKKEREEEQL----KGGHLPPKPA------------GPCG-----
UniRef90_M3Z8M0_1_445             VRMEEKKKEREEELL----KSD----GPA------------ARCGSGSHS
UniRef90_G1Q1M8_1_411             VRMEEKKKGREEEKL----KGGSLHLRPD------------GQCGPRNHS
UniRef90_G1PYG2_1_394             VRMEEKKKGREEEQA----KGES------------------------DHG
UniRef90_L5L5Q1_1_430             VRTEQKRKERDAEQP----NGDG------------------------DRG
UniRef90_UPI0002BCF55A_1_410      VRMEEKRKEREEERL----KADGP------------------------PL
UniRef90_P41987_1_407             VRMEEKRKEREEEPP----KAAGP------------------------EG
UniRef90_J9JHI9_1_433             VRMEEKKKEREEELL----RAELPHPGPA------------ARCG---PG
UniRef90_Q9Y6H8_1_435             VRMEEKKKEREEEEQ----LKRE------------------SPSPKEPPQ
UniRef90_G3QHB2_1_399             VR---------------------------------------SPSPKEPPQ
```

```
UniRef90_F7B1T8_1_432          VRMEEKRKARQEEEP----PKTD------------------SPSPPEPPQ
UniRef90_L9L2D3_1_460          VRMEEKKKEREEELL----KRDNPHPGAAGPCVPSGREALGGTSPEKPPV
UniRef90_Q64448_1_417          VRMEEKKKEREEELL----RRDNPQHGR-------------GREPMR-TG
UniRef90_G3H8J5_1_417          VRMEEKKREREEELL----RRDNPQHGR-------------GREPMR-TA
UniRef90_UPI0003333DA4_1_405   VRMEEKKREREE----------NPQHS---------------HEPLR-IG
UniRef90_I3NC37_1_417          VRMEEKKKEREEELL----RRENPHLER-------------GQESLDGAG
UniRef90_H0XD37_1_430          VRMEEKKKEREEELL----KRDSLQQG--------------VASPKD---
UniRef90_G1U3S9_1_407          VRTTPHHDAAELCVP--------AGHQE-------------PLGSPGTQD
UniRef90_UPI00032AEFC1_1_413   VRMEEKKKEREEELL----RRNHGGHGE-------------SLDTTGAQD
UniRef90_UPI00034F30A8_1_402   VRREEKRKEREEELL----RRDSPHPAR-------------ARALRGGPG
UniRef90_UPI0003340A67_1_370   VRMEEKRKARAEVPQ----RADSPHAA---------------WALQAAVD
UniRef90_UPI0002234D0E_1_449   VRREEKKKEQEEELL----SRDSPHPGAG------------DQEPPDRTG
UniRef90_G3TTN5_1_429          VRREEKKKEQEEELL----SRDSPHPGAG------------DQEPPDRTG
UniRef90_UPI0002C457ED_1_447   VRREEKKKEHEEEQL----SRDNPQPGAG------------GPEPPDRTM
UniRef90_UPI000333D50E_1_427   VRMEEKQQRGEE--------------------------------PLARTG
UniRef90_UPI0003343FBC_1_423   ARVEEKKKEREEELQ----KGAGLH-----------------------PS
UniRef90_F7G5V2_1_446          VRMEEKKKEKE-DLL----KKDNLH------------------QGESARM
UniRef90_G3VY19_1_428          VRMEEKKKEKEGEQL----KKNHLQG----------------VGNEGTRM
UniRef90_UPI000155BBAC_1_459   VRMEEKKKEKEEELL----KKTDPQAGPV------------GQGAPGSPR
UniRef90_F7ETY8_1_425          VRM----------LL----KKTDPQAGPV------------GQGAPGSPR
UniRef90_I3KXW1_1_474          VRMEEKRKEKEEEMR----KANRLQEEKELLYR-----------NGGDVG
UniRef90_UPI00022B3AE8_1_449   VRMEEKRKEKEEEMR----KANRLQEEKELLYR-----------NGGDVG
UniRef90_UPI0002A48057_1_447   VRMEDKRKEKEEEMR----KANKFHEEKELLYR-----------NGDDAG
UniRef90_H2MCD5_1_421          VRMEDKRKEKEEEMR----K------------R-----------NGDDAG
UniRef90_H2UTF3_1_420          VRMEEKRKEKEEELR-----------------------------NGGDAG
UniRef90_UPI000298F1F6_1_460   VRMEEKRKEKEEELR----KANRLQEEKELLYR-----------NGGDAG
UniRef90_H3DE08_1_376          VRMEEKRKEKEEEMR----KANRFQEEKELLYR-----------NGGDAG
UniRef90_Q8JFD2_1_434          VRMEEKRKEREEELR----KASRLQEEKELLYR-----------NGGGGE
UniRef90_H2T4Y1_1_438          VRMEEKRREREEDLR----KAGRHQEDHDPLYH-----------NGVSNG
UniRef90_H2T4Y2_1_428          VRMEEKRREREEDLR----KAGRHQEDHDPLYH-----------NGVSNG
UniRef90_H3BZL0_1_378          VRMEEKRREREEELR----K------------------------NGVSNG
UniRef90_I3K1N0_1_478          VRMEEKRREREEELR----KAGRHQDDHDPLYH-----------NGVGD-
UniRef90_H2LFL5_1_470          VRMEEKRREREEELR----KAGRLQGDHDPLYH-----------NGIGDG
UniRef90_M7BWJ7_1_486          VRMEEKKKE--EELK----KKGSSRDSNHQGG--------AAAAGGRGSS
UniRef90_K7F163_1_492          VRMEEKKKE--AELK----KKGSSRXXXXXXXXXXXXXX-XXXXXGRGSS
UniRef90_H0ZM32_1_512          VRMEEKRKEK-EELK----KKGNSKDGNYPVAA-------ASVSGGGGAS
UniRef90_H3AH16_1_396          VRMEEKRKEK-EELA----KCNKSQEDKELLLK-----------KGGG--
UniRef90_B0BM18_1_388          VRMEEKRKEKEEELK----KCIKGASEKEHLIK-----------GGE---
UniRef90_Q4S8R0_1_345          VRMEEKEKQKEKE-------LAAQSEKQ----------------QQLLGN
UniRef90_I3KYH7_1_341          VRMEEKEKQKQKE-------LEDQ--------------------QQVLCH
UniRef90_H2M0G7_1_345          VRMEEKENQKLKE-------LEIQNENQ----------------QQLLSK
UniRef90_H3A471_1_360          IRKEEKEKQREKEGQ----EKSGHHGDK----------------QPLLGE
UniRef90_Q6GLA5_1_353          VRLEEKRKQKEKLRQ----SSRDPQDDK----------------QLLLVP
UniRef90_UPI0002A4A1D5_1_346   VRMEEKHKEREHL------------LDQ----------------QGLIGA
UniRef90_S4RXA6_1_456          MRMESRRKEELEEWR----KLHPNEDEDDCKALLD-------GGKGRKGG
UniRef90_P16863_1_360          MRKEEKLNRKEEELK----MVQNEGGNVDMHLKQI-------EIKKFKYG
UniRef90_H2LU51_1_404          LRALEKERHCKKVALRRELEGVDTELVEVKR--------------RIEKE
UniRef90_I3KYL3_1_401          LRALEKERHCKKVALRRELEAVDAELTEVKK--------------RIEKE
UniRef90_G3NC44_1_402          LRALEKERHCKKVALRRELEAVDAELVEVRR--------------RIERE
UniRef90_H2SSL2_1_392          LRALEKERHCKKVALRREMEAVDVELVEVRK--------------RIEKE
UniRef90_UPI0002C46B7E_1_433   LRVLEKERQEMKTQLRRELEGAESEVPGDRW--------------RLEQE
UniRef90_G3ULF1_1_432          LRVLEKERQEMKTQLRRELEEVEFEVTGHRR--------------RLEQE
UniRef90_C9IYK1_1_407          LRVLEEERQRMKAQLRVELEEVEFEMPRDRR--------------RLEQE
UniRef90_H0Y0G7_1_436          LRVLEEERQRMKAQLRGELEGVEFEMSGDRR--------------RLEQE
UniRef90_L9JD22_1_408          LRVLEKERQRMKAQLRAELEGIEFEMLGDRR--------------RLERE
UniRef90_F1N617_1_436          LRVLEKQRQMKKEQLRGELEEAELETPGDRR--------------RLEEE
UniRef90_F1SV25_1_385          LRVLEKERQVKKAQLRGELEGVELEMPGDRK--------------RLEQE
UniRef90_L5JUH5_1_307          LRVLEKERQREKAQLRGELKGLEFEVSGDRR--------------KLEKE
UniRef90_UPI00020369CA_1_441   LRALEKERQKKKAQVRVELESTELEMTEYRK--------------RLERE
UniRef90_R4GLX1_1_405          LRALEKERQKKKAQVRVELESTELEMTEYRK--------------RLERE
UniRef90_UPI00035A19E6_1_466   LRALEKERQRKKAQVRVELESTELEMTENRK--------------RLERE
UniRef90_F6WA84_1_463          LRALEKDRQRKKAQLKGELEATEFEMVEDRR--------------KLERE
UniRef90_G1SG18_1_430          LRDFEKERQRKKSYLRAQMENPELDLEDQQ---------------RIDRE
UniRef90_UPI00032B0E64_1_413   LRDFEKERQRKKSYLRTQIENPELNIEEQQ---------------RIDRE
UniRef90_UPI0002C65D9D_1_412   LRAFEKERQRKKSHLRAQMENPELDLDEQQ---------------RIERE
UniRef90_F7CF28_1_383          LRAFEKERQRKKSHLRALMENPELEFDEQQ---------------RIDRE
UniRef90_Q969M2_1_435          LRAFEKDRQRKKSHLRAQMENPDLDLEEQQ---------------RIDRE
UniRef90_L9JVZ3_1_406          LRAFEKERQRKKSHLRAQLENPELDLEEQQ---------------RIDRE
UniRef90_I3M407_1_414          LRDFEKERHRRKSHLRVQMENPELDLEEQQ---------------RIDRE
UniRef90_L5JMB7_1_435          LRAFEKERQKKKSHLKDHREKPGLELEEQQ---------------RIDRE
UniRef90_L8J083_1_417          LRAFEKERQRKKSQLRAQMENPELELEDQQ---------------RMDRE
UniRef90_UPI0002BCCFF6_1_421   LRAFEKETRRKKSELTAQMENPELELEEQQ---------------NINRE
UniRef90_F1RY20_1_421          LRAFEKERQRKKSQLRAQMENPELELEEQQ---------------RLDRE
UniRef90_UPI0002C59166_1_412   LRAFEKERQKKKSHLRVQLGNPELELEEQQ---------------RIDRE
UniRef90_G3TBX7_1_436          LRALEKERWRKKSYLRTQMENPEVDLEEQQ---------------RIHRE
UniRef90_G3GSS7_1_405          LRDFEKQRQKKKSHLRAQMEDPRLELEEQQ---------------RVERE
```

```
UniRef90_UPI0003316158_1_413      LRTFEKERQKRKAHLRAHLEDPELEWEEQQ---------------RIERE
UniRef90_G3WUN4_1_443             LRAFEKEKQRKKVQLRAQLEEPELDQEEYQ---------------RIERE
UniRef90_F6W379_1_264             LRALEKERQKKKAQLRAELEELEVVVDEHR---------------KLERE
UniRef90_M7BL54_1_431             LRALEKERQKKKTHLRAQLEELEPVPEEHK---------------RMERE
UniRef90_UPI0002B44362_1_276      LRALEKERQNRKAHLRAQLEGLEPMLEEHK---------------RVERE
UniRef90_UPI000298933C_1_429      LRTLEKERHRRKVCLKAELEGTDPIQEDHK---------------RIERE
UniRef90_H3CDZ2_1_426             LRTLEKERHKRKACLKAELEGTDPVQEDHR---------------RIERE
UniRef90_I3IZH7_1_440             LRTLEKERHRKKACLKAELEGTDPVLEDHK---------------RIEKE
UniRef90_E7F3P4_1_389             LRALEKERHKKKVQLKVELEESE-ALEEHK---------------RIEKE
                                   :

UniRef90_UPI0002C691BE_1_358      K-----------AELSCWEEENGKIVLQGTLLNTYVCSILIRTTMEVAFI
UniRef90_F7BKC0_1_358             K-----------TALSCWEEGNEKIVLQGTLLNTYVCSILIRTTMEVAFI
UniRef90_Q0VCR2_1_359             K-----------TELSCWEEVNGRIALQGSLLNTYVCSILIRTTMEVAFI
UniRef90_G3TUX5_1_358             K-----------TELSCWEEVNGKVVLQGALLNTYVCSILIRTTMEVTFI
UniRef90_L9JKN2_1_358             K-----------TELSCWEEVNGKIVLQGTLLNTYVCSILIRTTMEVAFI
UniRef90_P36382_1_358             K-----------AELSCWEEGNGRIALQGTLLNTYVCSILIRTTMEVGFI
UniRef90_UPI0003315E33_1_350      K-----------TGLSCWEEVNGKIVLQGTLLNTYVCSILIRTTMEVAFI
UniRef90_UPI000333FB64_1_358      K-----------TELTCWEEVNGKVVLQGTLLNTYVCSILIRTTMEVAFI
UniRef90_UPI000334540D_1_357      K-----------PELSCWEEVNGRIVLQGTLLNTYVCSILIRTTMEVAFI
UniRef90_L5K3R8_1_358             K-----------AELSCWEEANGRIVLRGTLLNTYVCSILIRTTMEVAFI
UniRef90_G1TQ37_1_358             K-----------AELSRWEEVNGRILLRGTLLRTYVCSIVIRTTVEVAFI
UniRef90_UPI0001776F40_1_352      K-----------TELSCWEEVNGRIVLQGTLLHTYVCSIIIRTTTEVAFI
UniRef90_G3H2Q9_1_358             K-----------AELSCWKEVDGKIVLQGTLLNTYVCTILIRTTMEVAFI
UniRef90_Q01231_1_358             K-----------AELSCWKEVDGKIVLQGTLLNTYVCTILIRTTMEVAFI
UniRef90_G5BBL6_1_357             K-----------PELSCWEEMNGKVVLQGNLLNTYVCTILIRTTMEVAFI
UniRef90_H0WCZ7_1_358             K-----------PELSCWEEMNGKVVLQGNLLNTYVCTILIRTAMEVAFI
UniRef90_G3X1A3_1_358             K-----------AELSCREELDGKIILQGTLLNTYVCSILIRTAMEVAFI
UniRef90_H0ZTI1_1_370             KE----------TKLSGWDEAGGQIILRGSLLNTYVYSILIRTAMEVAFI
UniRef90_P18860_1_369             K-----------TELSCWDESGGKIILRGSLLNTYVYSILIRTAMEIAFI
UniRef90_G1KPB5_1_370             K-----------AELSCWDDSNGRIILQGTLLNTYVCSILIRTGMEVAFI
UniRef90_K7FWT6_1_369             K-----------AEFSSREESNGKIILQGSLLNTYVYSILIRVAMEVTFI
UniRef90_F1QL21_1_400             H------------KEKFENTKT-KIHLKGALLQTYVLSIVIRLVMEVTFI
UniRef90_UPI0002A497DC_1_408      D------------EEKGERPPVGRIRLRGALLKTYVLSILIRSVMEVVFL
Input_protein_seq                 QGS-------VKKSS-GS-KGTKKFRLEGTLLRTYICHIIFKTLFEVGFI
UniRef90_UPI00027FC10C_1_397      ---------------------------AGTLLRTYICHIIFKTLFEVGFV
UniRef90_L9JGQ0_1_441             QGS-------IRKSS-SSSKGTKKFRLEGTLLRTYICHIIFKTLFEVGFI
UniRef90_P28236_1_440             QAS-------IRKSS-SSSKGTKKFRLEGTLLRTYVCHIIFKTLFEVGFI
UniRef90_G5BBL7_1_440             QAS-------IRKSN-SSSKGTKKFRLEGTLLRTYVCHIIFKTLFEVGFI
UniRef90_M3XED1_1_440             QGS-------VKKGS-NSSKGTKKFRLEGTLLRTYICHIIFKTLFEVGFV
UniRef90_L5K685_1_440             QGS-------IKKSS-SSSKGTKKFRLEGTLLRTYICHIVFKTLFEVGFV
UniRef90_F6VG96_1_441             QCS-------IKKGG-GGSKGTKKFRLEGTLLRTYICHIVFKTLFEVGFI
UniRef90_P55917_1_440             QGS-------VKKSS-SSSKGTKKFRLEGTLLRTYVCHIIFKTLFEVGFI
UniRef90_UPI000329268E_1_442      QGS-------LKKSS-SSSKGTKKFRLEGTLLRTYICHIIFKTLFEVGFV
UniRef90_G3UBH4_1_410             QGS-------IKKSS-SS-RSTKKFRLEGTLLRTYICHIIFKTLFEVGFI
UniRef90_I3N7I6_1_431             QGS-------VRKGS-GSSQGAKKFRLEGTLLRTYLCHIVFKTLFEVGFV
UniRef90_G3X210_1_429             QGS-------TKKSS-NSTKGTKKFRLEGTLLRTYICHIIFKTLFEVGFI
UniRef90_F7BI06_1_372             QGS-------TKKSS-NSTKGTKKFRLEGTLLRTYICHIIFKTLFEVGFI
UniRef90_UPI000333FA9E_1_424      QGS-------LKKGS-SCSRSTKQFRLEGTLLRTYVCHVIFKTLFEVGFI
UniRef90_K7EY71_1_406             QED-------IRNTNEINPKGAKKFRLEGTLLGTYICHIIFKAIFEVGFI
UniRef90_M7BCA4_1_394             QES-------TRNTNEINPKGAKKFRLEGTLLGTYICHIIFKAIFEVGFI
UniRef90_P36381_1_400             QNK-------GN-----NPDGTKKFRLEGTLLRTYILHIIFKTLFEVGFI
UniRef90_H2MUH4_1_428             QGS-------VRTTKETSTKGSKKFRLEGTLLRTYICHIIFKTLFEVGFV
UniRef90_UPI00022B4A0D_1_396      QGS-------VRTTKETSTKGSKKFRLEGTLLRTYICHIIFKTLFEVGFV
UniRef90_H3D8V1_1_361             QGS-------VRTTKETSTKGSKKFRLEGTLLRTYICHIIFKTLFEVGFV
UniRef90_G3NHJ7_1_420             QGS-------VRTTKETSTKGSKKFRLEGTLLRTYICHIIFKTLFEVGFV
UniRef90_Q503J6_1_391             QGS-------VRTAKETSTKGSKKFRLEGTLLRTYICHIIFKTLFEIGFV
UniRef90_F6QCF2_1_438             QGS-------IRTTKETSTKGTKKFRLEGTLLRTYICHIIFKTLFEVGFV
UniRef90_UPI00034FAB61_1_453      QGS-------IRTTKETSTKGTKKFRLEGTLLRTYICHIIFKTLFEVGFV
UniRef90_UPI0001DE8510_1_439      QGS-------IRTTKETSTKGTKKFRLEGTLLRTYICHIIFKTLFEVGFV
UniRef90_H3A837_1_370             QGS-------VRTTKDTSTKATKKFRLEGTLLRTYICHIVFKTLFEVGFV
UniRef90_G1KPB7_1_391             QKS-------P-----VSTKGTKRFRLEGTLLRTYICHIFFKTLFEVGFI
UniRef90_B0S6Z4_1_382             QGS-------VRTAKETSTKGSKKFRLEGTLLCTYICHIIFKALFEIGFV
UniRef90_G1PZ72_1_426             ---------------LSWALPLRAPRLEGPLLRTYVGHIACKSLLEAGFV
UniRef90_UPI000155FB82_1_448      Q------------DTPPVRDDRGKVRIAGALLRTYVFNIIFKTLFEVGFI
UniRef90_F6U7L2_1_428             Q------------DTPPVRDDRGKVRIAGALLRTYVFNIIFKTLFEVGFI
UniRef90_UPI0002C6341D_1_440      ---------------PPVRDDRGKVRITGALLRTYVFNIIFKTLFEVGFI
UniRef90_M3Z8M0_1_445             Q-----------KDKPPVRDDRGKIRIAGALLRTYVFNIIFKTLFEVGFI
UniRef90_G1Q1M8_1_411             Q-----------KDKFILQDNRGKVRIAGALLRTYIFNIIFKTLFEVGFI
UniRef90_G1PYG2_1_394             Q-----------KNRPLVRDDQGKVLIAGALLRTYIFNIIFKMLFEVGFI
UniRef90_L5L5Q1_1_430             Q-----------RDRSPVRDDRGKVRIAGALLRTYVFNIIFKTLFEVGFI
UniRef90_UPI0002BCF55A_1_410      G-----------QDRPPVRDDRGKVRLAGALLRTYVFNIIFKTLFEVGFI
UniRef90_P41987_1_407             H-----------QDPAPVRDDRGKVRIAGALLRTYVFNIIFKTLFEVGFI
UniRef90_J9JHI9_1_433             G-----------QKDRPLRDDRGRVRIAGALLRTYVFNIIFKTLFEVGFI
UniRef90_Q9Y6H8_1_435             -------------DNPSSRDDRGRVRMAGALLRTYVFNIIFKTLFEVGFI
UniRef90_G3QHB2_1_399             -------------DNPSSRDDRGRVRMAGALLRTYVFNIIFKTLFEVGFI
```

```
UniRef90_F7B1T8_1_432             -------------DRPSSREDRGRVRMAGALLRTYVFNIIFKTLLEVGFI
UniRef90_L9L2D3_1_460             R-----------EDPGKVRDDRGKVRIAGALLRTYVFNVIFKTLFEVGFI
UniRef90_Q64448_1_417             S-----------PRDPPLRDDRGKVRIAGALLRTYVFNIIFKTLFEVGFI
UniRef90_G3H8J5_1_417             S-----------PRDPPLRDDRGKVRIAGALLRTYVFNIIFKTLFEVGFI
UniRef90_UPI0003333DA4_1_405      G--------------IPVRDDRGKVRIRGALLRTYVFNIIFKTLFEVGFI
UniRef90_I3NC37_1_417             S-----------QDKTVVRDDRGKVRIAGALLRTYVFNIIFKTLFEVGFI
UniRef90_H0XD37_1_430             --------------KLPVRDDRGKVRIAGALLRTYVFNIIFKTLFEVGFI
UniRef90_G1U3S9_1_407             K-----------PAAP-VRDDRGKVRIAGALLRTYVFNIIFKTLFEVGFI
UniRef90_UPI00032AEFC1_1_413      L-----------PAAP-VRD--GKVRIAGALLRTYVFNIIFKTLFEVGFI
UniRef90_UPI00034F30A8_1_402      A-----------PDQAPGSEERGRVRMAGALLRTYVFNVIFKTLFEVGFI
UniRef90_UPI0003340A67_1_370      T-----------QELA-GREERGRVRMAGALLRTYVFNIIFKTLFEVGFI
UniRef90_UPI0002234D0E_1_449      K-----------REKLPVRDDRGRVRIAGALLRTYVFNIIFKTLFEVGFI
UniRef90_G3TTN5_1_429             K-----------REKLPVRDDRGRVRIAGALLRTYVFNIIFKTLFEVGFI
UniRef90_UPI0002C457ED_1_447      K-----------RKKPPVRDDRGRVQIAGALLRTYVFNIIFKTLFEVGFI
UniRef90_UPI000333D50E_1_427      K-----------RQKLPIRDDRGRVRIAGALLRTYVFNIIFKTLFEVGFI
UniRef90_UPI0003343FBC_1_423      A-----------KDRPPALDELGRVRIAGALLRTYVFNIVFKMLFEVGFI
UniRef90_F7G5V2_1_446             E----------KKERPPIRDDRGKVRIAGALLRTYVFNIIFKTLFEVGFI
UniRef90_G3VY19_1_428             E----------KKEKPPIRDDRGKVRIAGALLRTYVFNIIFKTLFEVGFI
UniRef90_UPI000155BBAC_1_459      EPLLGREVAGGGKERPPVRDEWGKVRIAGALLRTYVFNIVFKTLFEVGFI
UniRef90_F7ETY8_1_425             EPLLGREVAGGGKERPPVRDEWGKVRIAGALLRTYVFNIVFKTLFEVGFI
UniRef90_I3KXW1_1_474             GGGGG---GGGKKEKPPIRDEHGKIRIRGALLRTYVFNIIFKTLFEVGFI
UniRef90_UPI00022B3AE8_1_449      GGGGG---GGGKKEKPPIRDEHGKIRIRGALLRTYVFNIIFKTLFEVGFI
UniRef90_UPI0002A48057_1_447      --------GGGKKEKPPIRDEHGKIRIRGALLRTYVFNIIFKTLFEVGFI
UniRef90_H2MCD5_1_421             --------GGGKKEKPPIRDEHGKIRIRGALLRTYVFNIIFKTLFEVGFI
UniRef90_H2UTF3_1_420             --------GGGKKEKPPIRDEHGKIRIRGALLRTYVFNIIFKTLFEVGFI
UniRef90_UPI000298F1F6_1_460      --------GGGKKEKPPIRDEHGKIRIRGALLRTYVFNIIFKTLFEVGFI
UniRef90_H3DE08_1_376             --------GGGRKEKPPIRDEHGKIRIRGALLRTYVFNIIFKTLFEVGFI
UniRef90_Q8JFD2_1_434             PGGRG---GGGKKEKPPIRDEHGKIRIRGALLRTYVFNIIFKTLFEVGFI
UniRef90_H2T4Y1_1_438             GSR-----GGGKKEKPPIRDEHGKIRIRGALLRTYIFNIIFKTLFEVGFI
UniRef90_H2T4Y2_1_428             GSR-----GGGKKEKPPIRDEHGKIRIRGALLRTYIFNIIFKTLFEVGFI
UniRef90_H3BZL0_1_378             GGR-----GGGKKEKPPIRDEHGKIRIRGALLRTYIFNIIFKTLFEVGFI
UniRef90_I3K1N0_1_478             --------GGGKKEKPPIRDEHGKIRIRGALLRTYIFNIIFKTLFEVGFI
UniRef90_H2LFL5_1_470             --------KGGKKDKRPIRDEHGKIRIRGALLRTYIFNIIFKTLFEVGFI
UniRef90_M7BWJ7_1_486             NNSSTNKEPFPKKEKPPIRDERGKIRISGALLRTYVFNIIFKTLFEIGFI
UniRef90_K7F163_1_492             NNNSTTKEEPPKKEKPPIRDERGKIRIGGALLRTYVFNIIFKTLFEVGFI
UniRef90_H0ZM32_1_512             SNVKDQSIVKRGKEKLPIRDERGRIRMGGALLRTYVFNIIFKTLFEVGFI
UniRef90_H3AH16_1_396             -----------KKQKLPIRDERGKIRISGALLRTYVFNIIFKTLFEVGFI
UniRef90_B0BM18_1_388             -----------KKEKPPIRDERGKIRIGGALLRTYVFNIIFKTLFEIGFI
UniRef90_Q4S8R0_1_345             -----------KPKKAPIKDNQGHVRLQGALLRTYVFNIIFKTLFEVAFI
UniRef90_I3KYH7_1_341             -----------KDKKPSVKDKQGHARLQGALLRTYVFNIIFKTLFEVAFI
UniRef90_H2M0G7_1_345             -----------AAKKTSVKDNQGHVRLQGALLRTYVFNIIFKTLFEVAFI
UniRef90_H3A471_1_360             P----------KNPKVSIRDDQGKIRMRGAILRTYVFNIIFKTLFEVGFI
UniRef90_Q6GLA5_1_353             N----------KPAKPPVKDDLGKVRLRGALLRTYVFNVIFKTMFEVGFI
UniRef90_UPI0002A4A1D5_1_346      A----------KPNKPPIRDEKGRVRLQGELLRTYVFNVIFKTLFEVGFI
UniRef90_S4RXA6_1_456             P----------PKLRKTYVNDQGKVCIKGALLRTYVLSIFSRTIFEVGFI
UniRef90_P16863_1_360             ------------------LEEHGKVKMRGGLLRTYIISILFKSVFEVGFI
UniRef90_H2LU51_1_404             MK------------QLE-QGKLNKAPLRGSLLCTYVAHIVTRSVVEVSFM
UniRef90_I3KYL3_1_401             MR------------QLE-QGKLNKAPLRGSLLCTYVAHIVTRSVVEVSFM
UniRef90_G3NC44_1_402             MK------------LLE-QGKLNKAPLRGSLLCTYLVHIVTRSVVEVSFM
UniRef90_H2SSL2_1_392             MR------------QLE-QGKLNKAPLRGSLLCTYVAHIVTRSLVEVSFM
UniRef90_UPI0002C46B7E_1_433      LC------------QLE-QRKLNKAPLRGTLLCTYVIHIFTRSAVEVGFM
UniRef90_G3ULF1_1_432             LC------------QLE-QRKLNKAPLRGMLLCTYVIHIFTRSAVEVGFM
UniRef90_C9IYK1_1_407             LC------------QLE-KRKLNKAPLRGTLLCTYVIHIFTRSVVEVGFM
UniRef90_H0Y0G7_1_436             LC------------QLE-KRKLNKAPLRGTLLCTYVTHIFTRSVVEVGFM
UniRef90_L9JD22_1_408             LC------------QLE-QRKLNKAPLRGTLLCTYVIHIFTRSVVEVGFM
UniRef90_F1N617_1_436             LC------------QLE-QRKVNKVPLRGTLLCTYVIHIFTRSVVEVGFM
UniRef90_F1SV25_1_385             LY------------QLE-QWKLNKAPLRGTLLCTYVIHIFTRSVVEVGFM
UniRef90_L5JUH5_1_307             LY------------QLE-RRTLNKAPLRGTLLCTYVIHIFARSVVEVGFM
UniRef90_UPI00020369CA_1_441      LR------------QLD-QRKLNKAPLRGSLLCTYVIHIFTRSAVEVGFM
UniRef90_R4GLX1_1_405             LR------------QLD-QRKLNKAPLRGSLLCTYVIHIFTRSAVEVGFM
UniRef90_UPI00035A19E6_1_466      LR------------QLD-QRKLNKAPLRGSLLCTYVIHIFTRSAVEVGFM
UniRef90_F6WA84_1_463             LR------------QLE-QRKLNKAPLRGALLCTYVIHILTRSAVEIGFM
UniRef90_G1SG18_1_430             LR------------RLEEQKRIHKVPLKGCLLRTYVLHILTRSVLEVGFM
UniRef90_UPI00032B0E64_1_413      LR------------RLEEQKRIHKVPLKGCLLRTYVLHILTRSVLEVGFM
UniRef90_UPI0002C65D9D_1_412      LR------------RLEEQKRIHKVPLKGCLLRTYVLHILTRSVLEVGFV
UniRef90_F7CF28_1_383             LR------------RLEEQKRIHKVPLKGCLLRTYVLHILTRSVLEVGFI
UniRef90_Q969M2_1_435             LR------------RLEEQKRIHKVPLKGCLLRTYVLHILTRSVLEVGFM
UniRef90_L9JVZ3_1_406             LR------------RLEEQKRIHKVPLKGCLLRTYVLHILIRSVLEVGFM
UniRef90_I3M407_1_414             LK------------RLEEQKRIYKVPLKGCLLRTYVLHILTRSVLEVGFM
UniRef90_L5JMB7_1_435             LS------------GLEEQKRIHKVPLKGCLLRTYVLHILTRSVLEVGFI
UniRef90_L8J083_1_417             LR------------KLEEQKRIQKVPLKGCLLRTYVLHVLTRSVLEVGFM
UniRef90_UPI0002BCCFF6_1_421      LR------------KLEEQKKIQKVPLKGCLLRTYVLHILTRSVLEVGFM
UniRef90_F1RY20_1_421             LR------------KLEEQKRIQKVPLKGCLLRTYVLHILTRSVLEVGFM
UniRef90_UPI0002C59166_1_412      LK------------RLEEQKRIHKVPLKGCLLRTYILHILTRSVMEVGFM
UniRef90_G3TBX7_1_436             LR------------MLEEQKRIYKVPLKGCLLRTYILHILTRSMLEIGFM
UniRef90_G3GSS7_1_405             LR------------RLEEQKRIHKVPLRGCLLRTYILHILTRSMLEVGFM
```

```
UniRef90_UPI0003316158_1_413      LR------------RLEEHRRVQKVPLRGCLLRTYVLHVLTRSVLEVGFM
UniRef90_G3WUN4_1_443             LR------------SLEEQRKVHKAPLKGCLLRTYILHILIRSVLEVGFM
UniRef90_F6W379_1_264             LR------------KLEEQRKVNKAPLRGSLLRTYVLHILTRSVVEVAFM
UniRef90_M7BL54_1_431             LR------------KLEEQKKVNKAPLRGSLLRTYILHILTRSAVEVGFM
UniRef90_UPI0002B44362_1_276      LR------------KLEEQKKVNKAPLRGSLLRTYVLHILTRSVVEVGFM
UniRef90_UPI000298933C_1_429      LR------------KLDEQKRVRKAPLRGSLLRTYVLHILTRSAVEVGFI
UniRef90_H3CDZ2_1_426             LR------------KLDEQKRVRKAPLRGSLLRTYVLHILTRSVVEVAFI
UniRef90_I3IZH7_1_440             LR------------KLDEQKKVRKAPLRGSLLRTYVFHILTRSVVEVGFI
UniRef90_E7F3P4_1_389             LR------------KLEEQKKVRKAPLRGSLLRTYVFHILTRSVVEVGFI
                                                              * :* **:  :  :   *  *:

UniRef90_UPI0002C691BE_1_358      VGQYLLYGIFLNTLHVCRRSPCPHPVNCYVSRPTEKNVFIVFMLAVAGLS
UniRef90_F7BKC0_1_358             VGQYLLYGIFLDTLHVCRRSPCPHPVNCYVSRPTEKNVFIVFMLAVAGLS
UniRef90_Q0VCR2_1_359             VGQYLLYGVFLDTLHVCRRSPCPHPVNCYVSRPTEKNVFIVFMLAVAGLS
UniRef90_G3TUX5_1_358             VVQYLLYGIFLDTLHVCRRSPCPHPVNCYVSRPTEKNVFIVFMLAVAALS
UniRef90_L9JKN2_1_358             VGQYLLYGIFLSTLHVCRRSPCPHPVNCYVSRPTEKNVFIVFMLAVAALS
UniRef90_P36382_1_358             VGQYFIYGIFLTTLHVCRRSPCPHPVNCYVSRPTEKNVFIVFMLAVAALS
UniRef90_UPI0003315E33_1_350      VGQYLLYGIFLAPLHVCRRSPCPHPVNCYVSRPTEKNVFIVFMLAVAALS
UniRef90_UPI000333FB64_1_358      VGQYLLYGILLDTLHVCHRSPCPHHVNCYVSRPTEKNVFIVFMLAVAGLS
UniRef90_UPI000334540D_1_357      VGQYLLYGIFLDTLHVCRRSPCPHPVNCYVSRPTEKNVFIVFMLAVAGLS
UniRef90_L5K3R8_1_358             VGQYLLYGVFLDTLHVCRRSPCPHPVNCYVSRPTEKNVFIVFMLAVAGLS
UniRef90_G1TQ37_1_358             VGQYLLYGIFLHTLHVCRRSPCPHPVNCYVSRPTEKNVFIVFMLAVAGLS
UniRef90_UPI0001776F40_1_352      VGQYLLYGIFLDTLHVCRRSPCPHPVNCYISRPTEKNVFIVFMLAVAGLS
UniRef90_G3H2Q9_1_358             VGQYLLYGIFLDTLHVCRRSPCPHPVNCYVSRPTEKNVFIVFMLAVAALS
UniRef90_Q01231_1_358             VGQYLLYGIFLDTLHVCRRSPCPHPVNCYVSRPTEKNVFIVFMMAVAGLS
UniRef90_G5BBL6_1_357             VGQYFLYGIFLNTLHVCRRSPCPHPVNCYVSRPTEKNVFIVFMLAVAGLS
UniRef90_H0WCZ7_1_358             VGQYFLYGIFLNTLHVCRRSPCPHPVNCYVSRPTEKNVFIVFMLAVAGLS
UniRef90_G3X1A3_1_358             VGQYFLYGIFLETLHVCRRKPCPHPVNCYVSRPTEKNVFIVFMLAVGGLS
UniRef90_H0ZTI1_1_370             VGQYVLYGIFLETLYICQRAPCPHPVNCYVSRPTEKNVFIVFMLAVAVLS
UniRef90_P18860_1_369             VGQYILYGIFLETLYICQRAPCPHPVNCYVSRPTEKNVFIIFMLAVAVLS
UniRef90_G1KPB5_1_370             VGQYMLYGIFLETLYVCNRQPCPHPVNCYVSRPTEKNIFIVFMLAVAALS
UniRef90_K7FWT6_1_369             VGQYILYGIFLKTLYICHREPCPHPVNCYVSRPTEKNVFIIFMLSVAALS
UniRef90_F1QL21_1_400             VIQYMMYGIFLDALYPCSMLPCPNPVNCYMSRPTEKNVFIVFMLVVAAVS
UniRef90_UPI0002A497DC_1_408      CLQYIMYGIFLNPLYVCKAWPCPHTVNCYVSRPTEKNIFIVFMLAVSAVS
Input_protein_seq                 VGHYFLYGFRILPLYRCSRWPCPNVVDCFVSRPTEKTIFILFMLSVASVS
UniRef90_UPI00027FC10C_1_397      VGHYFLYGFRILPLYRCSRWPCPNVVDCFVSRPTEKTIFILFMLSVASVS
UniRef90_L9JGQ0_1_441             VGHYFLYGFRILPLYRCSRWPCPNVVDCFVSRPTEKTIFILFMLSVAFVS
UniRef90_P28236_1_440             VGHYFLYGFRILPLYRCSRWPCPNVVDCFVSRPTEKTIFILFMLSVAFVS
UniRef90_G5BBL7_1_440             VGHYFLYGFRILPLYRCSRWPCPNVVDCFVSRPTEKTIFILFMLSVAFVS
UniRef90_M3XED1_1_440             VGHYFLYGFRILPLYRCSRWPCPNVVDCFVSRPTEKTIFILFMLSVASVS
UniRef90_L5K685_1_440             VGHYFLYGFRILPLYRCSRWPCPNVVDCFVSRPTEKTIFILFMLSVASVS
UniRef90_F6VG96_1_441             VGHYFLYGFRILPLYRCSRWPCPNVVDCFVSRPTEKTIFILFMLSVASVS
UniRef90_P55917_1_440             VGHYFLYGFRILPLYRCSRWPCPNVVDCFVSRPTEKTIFILFMLSVASVS
UniRef90_UPI000329268E_1_442      VGHYFLYGFQILPLYRCSRWPCPNVVDCFVSRPTEKTIFILFMLSVASVS
UniRef90_G3UBH4_1_410             VGHYFLYGFRILPLYRCSRWPCPNVVDCFVSRPTEKTVFILFMLSVASVS
UniRef90_I3N7I6_1_431             VGHYFLYGFRILPLYRCSRWPCPNVVDCFVSRPTEKTIFILFMLSVASVS
UniRef90_G3X210_1_429             VGHYFLYGFRILPLYQCSRWPCPNVVDCFVSRPTEKTIFILFMLSVACVS
UniRef90_F7BI06_1_372             VGHYFLYGFRILPLYQCSRWPCPNVVDCFVSRPTEKTIFILFMLSVASVS
UniRef90_UPI000333FA9E_1_424      VGHYFLYGYHILPLYRCSRWPCPNVVDCFVSRPTEKTIFIVFMLSVASVS
UniRef90_K7EY71_1_406             VGQYFLYGFRILPLYRCGRWPCPNLVDCFVSRPTEKTVFIMFMLAVASVS
UniRef90_M7BCA4_1_394             VGQYFLYGFRILPLYRCGRWPCPNLVDCFVSRPTEKTVFIMFMLAVASVS
UniRef90_P36381_1_400             VGQYFLYGFRILPLYRCGRWPCPNLVDCFVSRPTEKTIFIMFMLVVAAVS
UniRef90_H2MUH4_1_428             VGQYFLYGFRILPLYKCSRWPCPNTVDCFVSRPTEKTVFIIFMLAVACVS
UniRef90_UPI00022B4A0D_1_396      VGQYFLYGFRILPLYKCSRWPCPNTVDCFVSRPTEKTVFIIFMLAVACVS
UniRef90_H3D8V1_1_361             VGQYFLYGFRILPLYKCSRWPCPNTVDCFVSRPTEKTVFIIFMLAVACVS
UniRef90_G3NHJ7_1_420             VGQYFLYGFRILPLYKCSRWPCPNTVDCFVSRPTEKTVFIIFMLAVACVS
UniRef90_Q503J6_1_391             VGQYYLYGFRILPLYKCSRWPCPNTVDCFVSRPTEKTVFIIFMLAVACVS
UniRef90_F6QCF2_1_438             VGQYFLYGFWIKPLYRCSRWPCPNTVDCFVSRPTEKTIFITFMLAVAAVS
UniRef90_UPI00034FAB61_1_453      VGQYFLYGFWIKPLYRCSRWPCPNTVDCFVSRPTEKTIFITFMLAVAAVS
UniRef90_UPI0001DE8510_1_439      VGQYFLYGFWIKPLYRCSRWPCPNTVDCFVSRPTEKTIFITFMLAVAAVS
UniRef90_H3A837_1_370             VGQYFLYGFHILPLYRCGRWPCPNIVDCFVSRPTEKTVFIMFMLAVACVS
UniRef90_G1KPB7_1_391             VGQYFLYGFRILPLYRCGRWPCPNLVDCFVSRPTEKTIFIMFMLVVASVS
UniRef90_B0S6Z4_1_382             VGQYFLYGFRILPLYKCSRWPCPNTVDCYVSRPTEKTIFIIFMLAVACVS
UniRef90_G1PZ72_1_426             AGHYLLYGFRIPPLYRCSRWPCPNVVDCFVSRPTEKTIFILFMLCVAAVS
UniRef90_UPI000155FB82_1_448      AGQYFLYGFQLKPLYRCDRWPCPNTVDCFISRPTEKTIFIIFMLAVACLS
UniRef90_F6U7L2_1_428             AGQYFLYGFQLKPLYRCDRWPCPNTVDCFISRPTEKTIFIIFMLAVACLS
UniRef90_UPI0002C6341D_1_440      AGQYFLYGFELKPLYRCDRWPCPNTVDCFISRPTEKTIFILFMLAVACVS
UniRef90_M3Z8M0_1_445             AGQYFLYGFELKPLYRCDRWPCPNTVDCFISRPTEKTIFIIFMLAVACVS
UniRef90_G1Q1M8_1_411             AGQYFLYGFELKPLYHCDRWPCPNIVDCFISRPTEKTVFIIFMLVVACLS
UniRef90_G1PYG2_1_394             VGQYYLYGFQLKPLYHCNRWPCPNMVDCFISRPTEKTVFIIFMLVVACLS
UniRef90_L5L5Q1_1_430             AGQYFLYGFQLNPLYRCDRWPCPNTVDCFISRPTEKTIFIIFMLAVACLS
UniRef90_UPI0002BCF55A_1_410      AGQYFLYGFQLKPLYRCDRWPCPNTVDCFISRPTEKTIFILFMLAVACVS
UniRef90_P41987_1_407             AGQYFLYGFQLKPLYRCDRWPCPNTVDCFISRPTEKTIFILFMLAVACVS
UniRef90_J9JHI9_1_433             AGQYFLYGFELKPLYRCDRWPCPNTVDCFISRPTEKTIFIIFMLAVACVS
UniRef90_Q9Y6H8_1_435             AGQYFLYGFELKPLYRCDRWPCPNTVDCFISRPTEKTIFIIFMLAVACAS
UniRef90_G3QHB2_1_399             AGQYFLYGFELKPLYRCDRWPCPNTVDCFISRPTEKTIFIIFMLAVACAS
```

```
UniRef90_F7B1T8_1_432            AGQYFLYGFELQPLYRCDRWPCPNTVDCFISRPTEKTIFIIFMLAVACAS
UniRef90_L9L2D3_1_460            AGQYFLYGFELKPLYRCDRWPCPNTVDCFISRPTEKTIFIIFMLAVACAS
UniRef90_Q64448_1_417            AGQYFLYGFQLQPLYRCDRWPCPNTVDCFISRPTEKTIFVIFMLAVACAS
UniRef90_G3H8J5_1_417            AGQYFLYGFQLQPLYRCDRWPCPNTVDCFISRPTEKTIFVIFMLAVACAS
UniRef90_UPI0003333DA4_1_405     AGQYFLYGFQLQPLYRCDRWPCPNTVDCFISRPTEKTIFVIFMLAVACAS
UniRef90_I3NC37_1_417            AGQYFLYGFQLQPLYRCDRWPCPNMVDCFISRPTEKTIFIIFMLAVACAS
UniRef90_H0XD37_1_430            AGQYFLYGFELKPLYRCDRWPCPNTVDCFISRPTEKTIFIIFMLVVACAS
UniRef90_G1U3S9_1_407            AGQYFLYGFKLQPLYRCDRWPCPNTVDCFISRPTEKTIFIIFMLAVACAS
UniRef90_UPI00032AEFC1_1_413     AGQYFLYGFKLQPLYRCDRWPCPNTVDCFISRPTEKTIFIIFMLAVACAS
UniRef90_UPI00034F30A8_1_402     AGQSFLYGFQLQPLYRCDRWPCPNTVDCFISRPTEKTIFILFMLAVACAS
UniRef90_UPI0003340A67_1_370     AGQYFLYGFQLQPLYRCDRWPCPNTVDCFISRPTEKTIFILFMLAVACAS
UniRef90_UPI0002234D0E_1_449     IGQYLLYGFKLKPLYRCDQWPCPNTVDCFISRPTEKTIFIIFMLVVACVS
UniRef90_G3TTN5_1_429            IGQYLLYGFKLKPLYRCDQWPCPNTVDCFISRPTEKTIFIIFMLVVACVS
UniRef90_UPI0002C457ED_1_447     VGQYLLYGFKLKPLYRCDQWPCPNTVDCFISRPTEKTIFIMFMLVVACVS
UniRef90_UPI000333D50E_1_427     VGQYFLYGFELKPLYRCDRWPCPNTVDCFISRPTEKTIFIIFMLVVACVS
UniRef90_UPI0003343FBC_1_423     AGQYFLYGFKLKPLYRCAQWPCPNTVDCFISRPTEKTIFIIFMLAVACLS
UniRef90_F7G5V2_1_446            VGQYFLYGFELKPLYRCDRWPCPNTVDCFISRPTEKTIFIIFMLVVACVS
UniRef90_G3VY19_1_428            VGQYFLYGFELKPLYRCDRWPCPNTVDCFISRPTEKTIFIIFMLVVACVS
UniRef90_UPI000155BBAC_1_459     VGQYFLYGFELKPLYRCSRWPCPNTVDCFISRPTEKTIFIVFMLAVACVS
UniRef90_F7ETY8_1_425            VGQYFLYGFELKPLYRCSRWPCPNTVDCFISRPTEKTIFIVFMLAVACVS
UniRef90_I3KXW1_1_474            LGQYFLYGFQLRPLYKCARWPCPNTVDCFISRPTEKTIFIIFMLVVACVS
UniRef90_UPI00022B3AE8_1_449     LGQYFLYGFQLRPLYKCARWPCPNTVDCFISRPTEKTIFIIFMLVVACVS
UniRef90_UPI0002A48057_1_447     LGQYFLYGFHLKPLYKCARWPCPNTVDCFISRPTEKTIFILFMLVVACVS
UniRef90_H2MCD5_1_421            LGQYFLYGFHLKPLYKCARWPCPNTVDCFISRPTEKTIFILFMLVVACVS
UniRef90_H2UTF3_1_420            LGQYFLYGFQLRPLYKCGRWPCPNTVDCFISRPTEKTIFIIFMLVVACVS
UniRef90_UPI000298F1F6_1_460     LGQYFLYGFQLRPLYKCGRWPCPNTVDCFISRPTEKTIFIIFMLVVACVS
UniRef90_H3DE08_1_376            LGQYFLYGFQLRPLYKCARWPCPNTVDCFISRPTEKTIFILFMLVVACVS
UniRef90_Q8JFD2_1_434            LGQYFLYGFQLRPLYKCARWPCPNTVDCFISRPTEKTIFIIFMLVVACVS
UniRef90_H2T4Y1_1_438            LGQYFLYGFHLRPLYKCGRWPCPNTVDCFISRPTEKTIFIIFMLVVACIS
UniRef90_H2T4Y2_1_428            LGQYFLYGFHLRPLYKCGRWPCPNTVDCFISRPTEKTIFIIFMLVVACIS
UniRef90_H3BZL0_1_378            LGQYFLYGFHLRPLYKCGRWPCPNTVDCFISRPTEKTIFIIFMLVVACIS
UniRef90_I3K1N0_1_478            LGQYFLYGFQLRPLYKCGRWPCPNTVDCFISRPTEKTIFIIFMLVVACVS
UniRef90_H2LFL5_1_470            LGQYFLYGFRLRPLYKCARWPCPNTVDCFISRPTEKTIFIIFMLVVACVS
UniRef90_M7BWJ7_1_486            VGQYFLYGFELKPLYRCSRWPCPNIVDCFISRPTEKTIFIIFMLVVACVS
UniRef90_K7F163_1_492            VGQYFLYGFELKPLYRCSRWPCPNTVDCFISRPTEKTIFIIFMLVVACVS
UniRef90_H0ZM32_1_512            VGQYFLYGFELKPVYQCSRSPCPHTVDCFISRPTEKTIFIIFMLVVASVS
UniRef90_H3AH16_1_396            VGQYFLYGFELKPLYRCNRWPCPNSVDCFISRPTEKTIFIIFMLVVACVS
UniRef90_B0BM18_1_388            VGQYFLYGFQLKPLYRCSRWPCPNTVDCFISRPTEKTIFIIFMLVVACVS
UniRef90_Q4S8R0_1_345            VAQYFLYGFELKPMYTCDRWPCPNMVNCYISRPTEKTVFILFMLAVACIS
UniRef90_I3KYH7_1_341            VAQYFLYGFELKAMYTCDRWPCPNMVNCYISRPTEKTIFILFMLAVACVS
UniRef90_H2M0G7_1_345            VAQYFLYGFELKPMYTCDRWPCPNMVNCYISRPTEKTVFIIFMLGVACVS
UniRef90_H3A471_1_360            VAQYFLYGFELKPLYTCSRWPCPNTVNCYISRPTEKTIFILFMLAVACLS
UniRef90_Q6GLA5_1_353            VAQYFLYGFQLQPLYTCSRWPCPNTVNCYISRPTEKTIFIIFMLAVACLS
UniRef90_UPI0002A4A1D5_1_346     VAQYLLYGFELKPMYKCDRPPCTNVVNCYISRPTEKTIFIIFMLGVASLS
UniRef90_S4RXA6_1_456            VGQYFLYGFSLGPLYRCSRSPCPNVVDCFISRPTEKTIFILFMLVVACLS
UniRef90_P16863_1_360            IIQWYMYGFSLSAIYTCKRDPCPHQVDCFLSRPTEKTIFIWFMLIVSIVS
UniRef90_H2LU51_1_404            TGQYILYGHRLNTLYKCEREPCPNMVDCFVSRPTEKTVFMMFMQAIACIS
UniRef90_I3KYL3_1_401            TGQYILYGNRLSTLYKCEREPCPNVVDCFVSRPTEKTVFMMFMQAIACIS
UniRef90_G3NC44_1_402            VCQYFLYGHRLNPLYKCEREPCPNVVDCFVSRPTEKTVFMVFMQGIACIS
UniRef90_H2SSL2_1_392            MGQYILYGHHLKPLYKCEREPCPNVVDCFVSRPTEKTVFMMFMQAIACLS
UniRef90_UPI0002C46B7E_1_433     IGQYLLYGFHLQPLFKCHSHPCPNTIDCFISRPTEKTIFLLFMQSIATVS
UniRef90_G3ULF1_1_432            IGQYFLYGFYLEPLFKCHSHPCPNTIDCFISRPTEKTIFLLFMQSIATVS
UniRef90_C9IYK1_1_407            IGQYLLYGFHLEPLFKCHGHPCPNIIDCFVSRPTEKTIFLLFMQSIATIS
UniRef90_H0Y0G7_1_436            IGQYLLYGFHLDPLFKCHGHPCPNIIDCFVSRPTEKTIFLLFMQSIATIS
UniRef90_L9JD22_1_408            IGQYLLYGFHLEPLFKCHGYPCPNIIDCFVSRPTEKTIFLLFMQFIATIS
UniRef90_F1N617_1_436            IGQYLLYGFHLEPLFKCHGHPCPNTIDCFVSRPTEKTIFLLFMQSIATVS
UniRef90_F1SV25_1_385            IGQYLLYGFHLEPLYKCQGHPCPNIIDCFVSRPTEKTIFLLFMQSIATVS
UniRef90_L5JUH5_1_307            TGQYLLYGFHLEPLFKCHGHPCPNIIDCFVSRPTEKTIFLLFMQSIATVS
UniRef90_UPI00020369CA_1_441     IGQYLLYGFRLDPLYKCQRDPCPNVVDCFVSRPTEKTVFILFMQSIATVS
UniRef90_R4GLX1_1_405            IGQYLLYGFRLDPLYKCQRDPCPNVVDCFVSRPTEKTVFILFMQSIATVS
UniRef90_UPI00035A19E6_1_466     IGQYLLYGFHLDPLYKCQRDPCPNTVDCFVSRPTEKTVFILFMQSIATVS
UniRef90_F6WA84_1_463            IGQYLLYGFRLDPLFKCHRDPCPNTVDCFVSRPTEKTVFLLFMQSIAAAS
UniRef90_G1SG18_1_430            IGQYVLYGFQMYPLYKCTQPPCPNAVDCFVSRPTEKTIFMLFMQSIAAIS
UniRef90_UPI00032B0E64_1_413     IGQYILYGFQVYPLYKCTQPPCPNAVDCFVSRPTEKTIFMLFMHSIAAIS
UniRef90_UPI0002C65D9D_1_412     IGQYILYGFQMHPLYKCTQPPCPNAVDCFVSRPTEKTIFMLFMHSIAAIS
UniRef90_F7CF28_1_383            IGQYILYGFQMHPLYKCTQPPCPNAVDCFVSRPTEKTIFMLFMHSIAAIS
UniRef90_Q969M2_1_435            IGQYILYGFQMHPLYKCTQPPCPNAVDCFVSRPTEKTIFMLFMHSIAAIS
UniRef90_L9JVZ3_1_406            IGQYILYGFQMHPLYKCTQPPCPNAVDCFVSRPTEKTIFMLFMHSIAAIS
UniRef90_I3M407_1_414            IGQYILYGFQMRPLYKCTKPPCPNAVDCFVSRPTEKTIFMLFMHSIAAIS
UniRef90_L5JMB7_1_435            IGQYILYGFRMHPFYKCTQPPCPNAVDCFVSRPTEKTIFMLFMHSIAAVS
UniRef90_L8J083_1_417            VGQYILYGFQMHPLYKCTQPPCPNAVDCFVSRPTEKTIFMLFMHSIAAIS
UniRef90_UPI0002BCCFF6_1_421     IGQYMLYGFQMHPLYKCTQPPCPNAVDCFVSRPTEKTIFMLFMHSIAAIS
UniRef90_F1RY20_1_421            IGQYLLYGFQMHPLYKCIQPPCPNTVDCFVSRPTEKTIFMLFMHSIAAIS
UniRef90_UPI0002C59166_1_412     IGQYILYGFQMHPLYKCTQPPCPNAVDCFVSRPTEKTIFMLFMHSIAAIS
UniRef90_G3TBX7_1_436            IGQYILYGFQMHPLYKCTQSPCPNTVDCFVSRPTEKTIFMIFMHSIAAIS
UniRef90_G3GSS7_1_405            IGQYILYGFQMQPLYKCTRSPCPNAVDCFVSRPTEKTIFMLFMHSIAAIS
```

```
UniRef90_UPI0003316158_1_413      TGQYILYGFQMNPLYKCTQPPCPNVVDCFVSRPTEKTIFMLFMQSIAAVS
UniRef90_G3WUN4_1_443             IGQYILYGFQMHPLYKCNQFPCPNLVDCFVSRPREKTIFMLFMYSIAAIS
UniRef90_F6W379_1_264             IGQYLLYGFDLDPLYKCSRSPCPNIVDCFVSRPTEKTIFMVFMHSIAAVS
UniRef90_M7BL54_1_431             IGQYLLYGLQMYPLYKCTRPPCPNTVDCFVSRPTEKTIFMIFMHSIAAVS
UniRef90_UPI0002B44362_1_276      IGQYLLYGLHMSPLYKCTRPPCPNTVDCFVSRPTEKTIFMVFMNSIAAVS
UniRef90_UPI000298933C_1_429      VGQCALYGLGLSPLYKCARLPCPNSVDCFVSRPTEKNIFMVFMLVIAGVS
UniRef90_H3CDZ2_1_426             IGQCALYGLGLSPLYRCTRPPCPNTVDCFVSRPTEKNVFMVFMLVIAGVS
UniRef90_I3IZH7_1_440             IGQFALYGIGLSALYKCERLPCPNSIDCFVSRPTEKNIFMVFMLVIAGVS
UniRef90_E7F3P4_1_389             VGQYMLYGIGLTPLYKCERDPCPNSVDCFVSRPTEKNIFMIFMLVISGVS
                                    :  :**  : ... *   **.: ::*::*** **.:*: **  :.  *

UniRef90_UPI0002C691BE_1_358      LFLSLAELYHLGWKKIRQRFA---KSQQGMAECQLPGPSA--GIVQS---
UniRef90_F7BKC0_1_358             LFLSLVELYHLGWKRIRQRFA---RSRQGMAECQLPGPSA--GIVQK---
UniRef90_Q0VCR2_1_359             LFLSLAELYHLGWKKIRQRYV---KSQPGVGECQLPGPSA--GRVQS---
UniRef90_G3TUX5_1_358             LFLSLAELYHLGWKKIRHRFV---KSQHGMTESQLPVPST--GMAQS---
UniRef90_L9JKN2_1_358             LFLSLAELYHLAWKKIRQRFV---KSRQSMAECRLPGPSA--GIVQN---
UniRef90_P36382_1_358             LLLSLAELYHLGWKKIRQRFV---KPRQHMAKCQLSGPSV--GIVQS---
UniRef90_UPI0003315E33_1_350      LFLSLAELYHLGWKKLRQRRG---KSPQGS-------SSA--GIVQN---
UniRef90_UPI000333FB64_1_358      LFLSLAELYHLGWKKMRRRLL---KSGRALTESPLPAPSA--GLAQR---
UniRef90_UPI000334540D_1_357      LFLSLAELYHLGWKRVRQRFV---KRPQSLAECPLPGPAA--RLGQG---
UniRef90_L5K3R8_1_358             LVLSLAELYHLGWKKFRQRFA---KSRPGVAECQLPGPAA--GLAQS---
UniRef90_G1TQ37_1_358             LFLSLAELYHLGWKKIGQRLA---RSPQSSAEPRLPGPSA--GTAQS---
UniRef90_UPI0001776F40_1_352      LLLSLAELYHLGWKKIRQLFA---KSQQSTSQHHLPGCSA--GTTQN---
UniRef90_G3H2Q9_1_358             LFLSLAELYHLGWKKIRQRFV---KSRQGMDKQQLLRPSP--NLVQS---
UniRef90_Q01231_1_358             LFLSLAELYHLGWKKIRQRFG---KSRQGVDKHQLPGPPT--SLVQS---
UniRef90_G5BBL6_1_357             LFLSLVELFHLGWK-IKQRFI---QSRQSMAKCQLPGPSG--CAVQS---
UniRef90_H0WCZ7_1_358             LFLSLAELFHLGWR-IRQRFV---KSRQGTAERQLLGPST--GIVQN---
UniRef90_G3X1A3_1_358             LCLSLVELYHLGWKKAKRHFA---QPCPARVEASLPPPPA--GGVQN---
UniRef90_H0ZTI1_1_370             LFLSLAELYHLGWKKAKERCSRSYKASPSTAPGRLESAPQ-ADRPQM---
UniRef90_P18860_1_369             LFLSLAELYHLGWKKAKERCSRAYKPSPSTAPRRLESAPQ-VERAQM---
UniRef90_G1KPB5_1_370             LFLSLAELYHLAWKKVKQRFSRSCKNSPSSNNGILEAESQGKKMAQS---
UniRef90_K7FWT6_1_369             LFLSLAELYHLGWKKAKEKCAKSYKPSPTMVTSKLAPATQ-VEMAQS---
UniRef90_F1QL21_1_400             LLLSVIELYHLGWKQCKKCLRKHADKHANDKIQNVKAVSAIEPIRTSIPM
UniRef90_UPI0002A497DC_1_408      LVLSVMELHHLAWKHC--CRYRNLRRQSLSALKYLLCKRAASSANTSSPR
Input_protein_seq                 LFLNVMELGHLGLKGIRSALKRPVEQP---LGEIP------EKSLHSIAV
UniRef90_UPI00027FC10C_1_397      LFLNVMELGHLGLKGIRAAFKRPAEQP---LGEIP------EKSLHSIAV
UniRef90_L9JGQ0_1_441             LFLNIMEMSHLGLKEIRSAFKRPVEQP---LGEIP------KKSLHSIAV
UniRef90_P28236_1_440             LFLNIMEMSHLGMKGIRSAFKRPVEQP---LGEIA------EKSLHSIAV
UniRef90_G5BBL7_1_440             LFLNILEMSHLGLKGIRSAFKRPVEQP---VGEIP------EKSLHSIAA
UniRef90_M3XED1_1_440             LFLNILEMSHLGLKRIRSAFKRPVEQP---LGEIP------EKSLHSIAV
UniRef90_L5K685_1_440             LFLNILEMSHLGLKRIRSAFKRQVEQP---LGEIP------EKSLHSIAV
UniRef90_F6VG96_1_441             LFLNILEISHLGLKKIRSALKEPAEQP---LGEVP------EKSLHSIAV
UniRef90_P55917_1_440             LFLNILEMSHLGLKKIRSAFKRPVEQP---LGEIP------EKSLHSIAV
UniRef90_UPI000329268E_1_442      LFLNIMEMSHLGLKRIQSAFKKPVEPSR-SLGETP------EKPLHSIAV
UniRef90_G3UBH4_1_410             LFLNIMEISYLGLKRIRAAFKKPVEQP---LGEIS------ENPLHSIAV
UniRef90_I3N7I6_1_431             LFLNILEMSHLGLKGIRSAFRRPADQP---PAEMP------EKSLHSIAV
UniRef90_G3X210_1_429             LFLNIMEISHLGLKRIRSAFNRPTEQP---LGEIP------EKSLHSIAV
UniRef90_F7BI06_1_372             LFLNIMEISHLGLKRIRSAFNRPAEQP---LGEIP------EKSLHSIAV
UniRef90_UPI000333FA9E_1_424      LFLNVMEMSHLGLKRVRSAFTRPEEQP---LSDIP------EKSLHSIAV
UniRef90_K7EY71_1_406             LVLNLVEISYLSLKKIRLFFRRPTE---QQQGEIP------EKPLHSIAV
UniRef90_M7BCA4_1_394             LILNLVEISYLILKRIRLAFRRPTE---EQQREIP------EKPLHSIAV
UniRef90_P36381_1_400             LFLNLVEISHLILKRIRRALRRPAE---EQMGEVP------EKPLHAIAV
UniRef90_H2MUH4_1_428             LFLNFVEISHLGLKKIRFVFRKPAPAPSAAQGEGMAPLPPQGKSLPPLGV
UniRef90_UPI00022B4A0D_1_396      LFLNFVEISHLGLKKIRFVFRKPAPAPGPSQGEGSAPLPPQGKSLPPLAV
UniRef90_H3D8V1_1_361             LFLNFVEISHLGLKKIRFVFRKPE-------------------SLPSLAV
UniRef90_G3NHJ7_1_420             LFLNFVEISHLGLKKIRFVFRKPAPAP--AQGEGTAPLPPPGKNLPPLAM
UniRef90_Q503J6_1_391             LFLNFVEISHLGLKKIHFVFRKPVRPQVEGPG-------AAEKALPSIAA
UniRef90_F6QCF2_1_438             LFLNVVEISHLGWKKIRLAFRRPDQHPQQLPGEVS------QKSLHSIAI
UniRef90_UPI00034FAB61_1_453      LFLNVVEISHLGWKKIRLAFRRPDQHPQQLPGEVS------QKSLHSIAI
UniRef90_UPI0001DE8510_1_439      LFLNVVEISHLGWKKIRLAFRRPDQHPQQLPGEVS------QKSLHSIAI
UniRef90_H3A837_1_370             LFLNFVEISHLGWKKIRFAFKTP---MQQQQGELST-----EKPLHSIAV
UniRef90_G1KPB7_1_391             LFLNIVEISHLGLKKILNAFRKTRQQR---SVETP------EKPLHSIAV
UniRef90_B0S6Z4_1_382             LFLNFVEISHLGLKKIRFVFHRPAPAQLESLGPPE-------RSLPFLLT
UniRef90_G1PZ72_1_426             LLLNLLEMSHLGLKRVRSAFRGRVEPP-PPPGEGP------ETPLRSIAV
UniRef90_UPI000155FB82_1_448      LLLNMLEIYHLGWKKFKQGMTNHYS-PDTPGTGMGATKPEGVTPLHVPSN
UniRef90_F6U7L2_1_428             LLLNMLEIYHLGWKKFKQGMTNHYS-PDTPGTGMGATKPEGVTPLHVPSN
UniRef90_UPI0002C6341D_1_440      LFLNMLEIYHLGWKKFKQGMTNQYS-PDTPESRMGATKPGGVSPLLLPSN
UniRef90_M3Z8M0_1_445             LLLNMLEIYHLGWKKLKQGMTN-YR-SDTPESRVGATKPGGVSPLLLPSH
UniRef90_G1Q1M8_1_411             LLLNVLEIYHLGWKKFKQGMTNHYG-PDTVESRVGAAISG----------
UniRef90_G1PYG2_1_394             LLLNVLEIYHLGWKKFKQGMTNHYG-PDTVESRVGAAISG----------
UniRef90_L5L5Q1_1_430             LFLNVLEIYHLGWKKLKQGMTNYYS-VETPQSRLGAAKPGGVSPLPP-SL
UniRef90_UPI0002BCF55A_1_410      LLLNMLEIYHLGWKKLKQGMTDPYR-PDTPGSSVRAAKPACERPLLLPSH
UniRef90_P41987_1_407             LLLNVLEIYHLGWKKLKQGMTSPFR-PDTPGSRAGSVKPVGGSPLLLPPN
UniRef90_J9JHI9_1_433             LLLNMLEIYHLGWKKLKQGMTNHPR-ADSP----EATKPGGGRALGPPSL
UniRef90_Q9Y6H8_1_435             LLLNMLEIYHLGWKKLKQGVTSRLG-PDASEAPLGTADP-----PPLPPS
UniRef90_G3QHB2_1_399             LLLNMLEIYHLGWKKLKQGVTSRLG-PDASEAPLGTADP-----PPLPPS
```

UniRef90_F7B1T8_1_432	LLLNMLEIYHLGWKKLKQGMTS-LG-PDASEAPPGTADP-----PPLPRS
UniRef90_L9L2D3_1_460	LLLNMLEIYHLGWKKLKQGMTNHLG-PDPSEAREGAVDP-----PPLPSC
UniRef90_Q64448_1_417	LVLNMLEIYHLGWKKLKQGVTNHFN-PDASEARHKPLDP-----LPTATS
UniRef90_G3H8J5_1_417	LVLNMLEIYHLGWKKLKQGVTNHFS-PDASEARHKPLDP-----LPQASS
UniRef90_UPI0003333DA4_1_405	LVLNMLEIYHLGWKKLKQGVTNHFS-PEASEVRSMALDP-----LPRVSH
UniRef90_I3NC37_1_417	LVLNMLEIYHLGWKKFKQGVTDHFG-PDTSESKLVAAEP-----LPLASR
UniRef90_H0XD37_1_430	LLLNMLEIYHLGWKKLKQGMTNHLG-ADPAEAGVVATET-----LALPAG
UniRef90_G1U3S9_1_407	LVLNVIEIYHLGWKKLKQGVTNHLG-PDPCEVRLGAAEPP----LALAER
UniRef90_UPI00032AEFC1_1_413	LLLNMLEIYHLGWKKLKQGVTNHYG-ADPCEA--------------LAAR
UniRef90_UPI00034F30A8_1_402	LVLNVLEIYHLGWKKLKQGVTNHLG-PDAPDARLGLAEP-----LPLAAR
UniRef90_UPI0003340A67_1_370	LVLNMLEIYHLGWKKLKQGVTSHLG-PNGP--------------------
UniRef90_UPI0002234D0E_1_449	LLLNMLEIYHLGWKKFKQGVANHYS-PHAAETSMAATKPSGVNPLLPHSQ
UniRef90_G3TTN5_1_429	LLLNMLEIYHLGWKKFKQGVANHYS-PHAAETSMAATKPSGVNPLLPHSQ
UniRef90_UPI0002C457ED_1_447	LLLNMLEIYHLGWKKLKQGMTNHYS-PHAAETSSAATKPSDINPFLLPSQ
UniRef90_UPI000333D50E_1_427	LLLNMLEIYHLGWKKLKQGMTNCYS-P-------VAPKPGGINPLHLPSQ
UniRef90_UPI0003343FBC_1_423	LFLNLLEIYHLGWKKLKQGITNHYC-PEAS--VLVQKKPGGVGPLILRPH
UniRef90_F7G5V2_1_446	LLLNMLEIYHLGWKKLKQGMTNHYNSPDSPEAKVLPSKCSSIGPLLLSPH
UniRef90_G3VY19_1_428	LLLNMLEIYHLGWKKLKQGMTNHYN-PDSPEAKIVPSKCSSLGPLLLSSH
UniRef90_UPI000155BBAC_1_459	LLLNMLEIYHLGWKKLKQGMTNRYS-PDSPEAQRGLARPDQLTPFLLPSH
UniRef90_F7ETY8_1_425	LLLNMLEIYHLGWKKLKQGMTNRYS-PDSPEAQRGLARPDQLTPFLLPSH
UniRef90_I3KXW1_1_474	LLLNLLEIYHLGWKKVKQGMTNEYA-PDRESLRRIDIAEPECLASASRTA
UniRef90_UPI00022B3AE8_1_449	LLLNLLEIYHLGWKKVKQGMTNEYA-PDRESLRRIDIAEPECLASASRTA
UniRef90_UPI0002A48057_1_447	LLLNMLEIYHLGWKKVKKGMRNKFV-PYHESLRHVDIAEPECLASASRTA
UniRef90_H2MCD5_1_421	LLLNMLEIYHLGWKKVKKGMRNKFV-PYHESLRHVDIAEPECLASASRTA
UniRef90_H2UTF3_1_420	LLLNLLEIYHLGWKKVKQGVTNQFV-PDRESMRRVNIAEPECLALASRTA
UniRef90_UPI000298F1F6_1_460	LLLNLLEIYHLGWKKVKQGVTNQFV-PDRESMRRVNIAEPECLALASRTA
UniRef90_H3DE08_1_376	LLLNLLEIYHLGWKKLPPRLHGRDG-RQRG-------------LSAARGA
UniRef90_Q8JFD2_1_434	LLLNLLEIYHLGWKKVKQGMTNEFA-PDRESLPEADEAEPE----SPRTA
UniRef90_H2T4Y1_1_438	LALNLLEIYHLGWKKVKQGVTNEFV-PDGELLPQSADEHRDMEKIHEQTS
UniRef90_H2T4Y2_1_428	LALNLLEIYHLGWKKVKQGVTNEFV-PDGELLPQSADEHRDMEKIHEQTS
UniRef90_H3BZL0_1_378	LALNLLEIYHLGWKKVKQGVTNEFV-PDG---------------------
UniRef90_I3K1N0_1_478	LVLNLLEIYHLGWKKVKRGVTNEFA-PDSESLIMAEDEPTDAETSPEQTS
UniRef90_H2LFL5_1_470	LVLNLLEIYHLGWKKVKQGVTNEFI-PESES-ILGQHGPGDAETIPEQVS
UniRef90_M7BWJ7_1_486	LLLNMLEMYHLGWKKLKQGVTNQYR-PDPLPTAIAPATIARDSKPITLPL
UniRef90_K7F163_1_492	LLLNMLEMYHLGWKKLKQGMTNQYR-PDPHPAAMTPATTVRDSKPLTLPL
UniRef90_H0ZM32_1_512	LLLNMLEIYHLGWKKLKQGMTSQYI-LEMPVATMTPVMVTGESKPVSLPP
UniRef90_H3AH16_1_396	LLLNMLEIYHLGWKKLKQGVTNEFT-PDPVLACNKMESQSSTPAPRTAPP
UniRef90_B0BM18_1_388	LLLNMLEIYHLGWKKLKQGMTNRYI-PDPSCNKSEPPSPQTAPSSMSFPP
UniRef90_Q4S8R0_1_345	LLLNLVEMYHLGFTKCHQGL--RYRRSKTRKQSPKALHEP-----VMPFV
UniRef90_I3KYH7_1_341	LLLNLVEMYHLGFTKCHQGL--RYRSSKAAREGSKALSEP-----VMPFV
UniRef90_H2M0G7_1_345	LLLNLVEMYHLGFTKCHQGL--RYRRSRATNAAPKSPNEA-----VPPFA
UniRef90_H3A471_1_360	LLLNLIEMYYLGFTRCCQGLTNRYGSETGSKPCSEAPSEN-----AVPFV
UniRef90_Q6GLA5_1_353	LLLNLIEVYYLGFTKCRQGLS-PSHNETVTKLPTKTSSTT-----LTHII
UniRef90_UPI0002A4A1D5_1_346	LLLNLVEIYHLGFTKCRQGITFRRRQE-VAKSVSKQTNEA-----VVPIV
UniRef90_S4RXA6_1_456	LLLNLLEMYHLGWKKIRKGLGKRYEKMSQQQQQQQQQSAMKHHPPSLDQ
UniRef90_P16863_1_360	LALNIIELFYVTYKSIKDGIKGKKDPFSATNDAVISGKECGSPKYAYFNG
UniRef90_H2LU51_1_404	LFLSLLEIIHLAFKKIKKGILN--YYPHLKDDLDDYYVSKSKKNSVVHQV
UniRef90_I3KYL3_1_401	LFLSLLEIMHLSFKRIKKGILD--YYPHLKDDLDDYYVSKSKKDSVVHQV
UniRef90_G3NC44_1_402	LFLSLLEIMHLGFKKLKRGILD--YYPHLKADLDEYYVDKSKKDSVVHQV
UniRef90_H2SSL2_1_392	LFLSLLEIIHLGFKKLKKCILN--FFPHLKDDPDEFYISKSKKNSVVHQV
UniRef90_UPI0002C46B7E_1_433	LFLNVLEIAHLGFKKIKRGLWG---QYKLKDEHNELFENKSKQNSAKYKS
UniRef90_G3ULF1_1_432	LFLNVLEIAHLGFKKIKGGLWG---QYKLKDEHNEFFANKSKQNPTKYKS
UniRef90_C9IYK1_1_407	LFLNILEIFHLGFKKIKRGLWG---KYKLKKEHNEFHANKAKQNVAKYQS
UniRef90_H0Y0G7_1_436	LLLNILEIFHLGFKKIKRGLWG---KYKLKDDHNKFYANKSEQNLAKYQN
UniRef90_L9JD22_1_408	LFLNMLEIFHLGFKKIKRGLWG---QNKLKVEHNEFYANKSKQNLAKYEG
UniRef90_F1N617_1_436	LFLNVLEIFHLGFKKIKRGLWG---QYKLKDEHNEFCT-KSKQNLAKYQN
UniRef90_F1SV25_1_385	VFLNVLEICHLGFKKIKRGIWG---QYKLKDKQNEFCTNKSKQNLAKYQN
UniRef90_L5JUH5_1_307	LFLNVLEIFHLGFKKVKRGLWG---RYKLKDEHNEFYK--SKQNLAKYQS
UniRef90_UPI00020369CA_1_441	LLLNVLEIIHLGFRKIKIGLCG---QEENKDYRGNFYTNKSKKYSVIPRS
UniRef90_R4GLX1_1_405	LLLNVLEIIHLGFRKIKIGLCG---QEDNKDDRGNFYTNKSKKYSVIPRS
UniRef90_UPI00035A19E6_1_466	LLLNILEIIHLGFRKIKMGLCE---QNKNKDDYDNFYINKSKKYSVIPHS
UniRef90_F6WA84_1_463	LLLNVLEIVHLGLKRIKKGFERPKYKYKMNDECEDSDLSEAKIISAAQPP
UniRef90_G1SG18_1_430	LLLNVLEIFHLGVRKIMKALYEK--SSNEGFENERGPPFQLRKYSAAQQC
UniRef90_UPI00032B0E64_1_413	LLLNILEIFHLGVRKIMKALYEK--SSSEGVENERGPPFQLRKYSVAQQC
UniRef90_UPI0002C65D9D_1_412	LFLNVLEIFHLGIRKIMKALYEE--SSNEGLEDERGPPFHLKKYSVAQQS
UniRef90_F7CF28_1_383	LFLNVLEIFHLGIRKIMRALYER--SSNECIEEERGPPVRLKKYSVAQQS
UniRef90_Q969M2_1_435	LLLNILEIFHLGIRKIMRTLYKK--SSSEGIEDETGPPFHLKKYSVAQQC
UniRef90_L9JVZ3_1_406	LLLNILEIFHLGIKTITRALYEK--SSSEGIEYESGPPFHSKKNAVPQQC
UniRef90_I3M407_1_414	LLLNVLEIFHLGIRKIMRALYEK--SSSGSIENERGPPFHLKKYSGIQPC
UniRef90_L5JMB7_1_435	LFLNVLEIFHLGIRKIMRALYEN--PSKEGIEDERGPPFHFKKYSVVQQC
UniRef90_L8J083_1_417	LFLNILEIFHLGIKKIMRALYDK--SSSEGIEDERRLPFHLKKYSVTQEC
UniRef90_UPI0002BCCFF6_1_421	LFLNVLEIFHLGVRKIMRALYDK--SGGEGIEDERGPPFHLKKYSAAQQC
UniRef90_F1RY20_1_421	LFLSVLEIFHLGIRKIMRALYDK--SSSKGIEDERGPPFHLKKYSVAQQC
UniRef90_UPI0002C59166_1_412	LFLSALEIFHLGIRKIMRAFYEK--SSNEGTEDESSIPFHLKKYSVAQEC
UniRef90_G3TBX7_1_436	LFLNVLEIFHLGIRKIGRALYEK--PNSEVTEDERGPPFHLKKCSVAQQS
UniRef90_G3GSS7_1_405	LLLNILEIFHLGIRKIMRALDGK--SSSGNTENETGPPFHSTNYSGAQSC

```
UniRef90_UPI0003316158_1_413      LFLNGLEIFHLGIRKIIRAVHEK--PGGDSTESEKGPQLALEKYSVVQPC
UniRef90_G3WUN4_1_443             LFFNILEIFHLGIRKIMKALYGG--SSRD-TERE---LCHLNRNSVMQPC
UniRef90_F6W379_1_264             LFLNVLEIFHLGIRKIKQGLSGK--SN-----------------------
UniRef90_M7BL54_1_431             LFLNILEIIHLGIRKIKKTLHGR--QRREAVMAEETSICTLKKNSVVQQV
UniRef90_UPI0002B44362_1_276      LFLNILEIAHLGIKKIRKSLCG----------------------------
UniRef90_UPI000298933C_1_429      LFLNILEIFHLGVKRIKQSLYGY--KYRD----DESVYRSKKNSTVQQVC
UniRef90_H3CDZ2_1_426             LALNILEILHLGVKRIKQSLYGY--KYRD----DESVCRSKKNSTVQQVC
UniRef90_I3IZH7_1_440             LFLNLLEISHLGVKKIKQILYGY--KYGD----DESLCRSKKNSMVQQVC
UniRef90_E7F3P4_1_389             LFLNLLEIFHLGVKKIKQTIYGS--MYSD----DDSICRSKKNSMVQQVC
                                  : :.  *: ::

UniRef90_UPI0002C691BE_1_358      ----------------------------CTPPPDFYQCVENGPGGKFFN-
UniRef90_F7BKC0_1_358             ----------------------------CTPPLDFNQCLENGPGGKFFN-
UniRef90_Q0VCR2_1_359             ----------------------------CTPPPDFNQCLENGPGGKFFS-
UniRef90_G3TUX5_1_358             ----------------------------CTPPPDFNQCLENGPEGKFFN-
UniRef90_L9JKN2_1_358             ----------------------------CTPPPDFSQCLDNGPGGKFFN-
UniRef90_P36382_1_358             ----------------------------CTPPPDFNQCLENGPGGKFFN-
UniRef90_UPI0003315E33_1_350      ----------------------------CTPPPDFNHCLKNGSGGKLFN-
UniRef90_UPI000333FB64_1_358      ----------------------------CTPPPDFNQCLENGSGGKFFN-
UniRef90_UPI000334540D_1_357      ----------------------------CTPPPDFSQCLDHGPAGKFFN-
UniRef90_L5K3R8_1_358             ----------------------------CTPPPAFSQCLENGPGGKFFN-
UniRef90_G1TQ37_1_358             ----------------------------CTPPPDFTQCLENGPGGRFFS-
UniRef90_UPI0001776F40_1_352      ----------------------------CTPPPDFSQCLANGPGGKFFG-
UniRef90_G3H2Q9_1_358             ----------------------------LTPPPDFNQCLRSRPGEKFLS-
UniRef90_Q01231_1_358             ----------------------------LTPPPDFNQCLKNSSGEKFFS-
UniRef90_G5BBL6_1_357             ----------------------------CTLPPDFTQCLENGPRGKFFS-
UniRef90_H0WCZ7_1_358             ----------------------------CTPPPDFTQCLGTGPGGKFFS-
UniRef90_G3X1A3_1_358             ----------------------------CTPPPDFNECVEGSPNQKFYGS
UniRef90_H0ZTI1_1_370             ----------------------------YTPPPDFNQCLSS-PNGKFIS-
UniRef90_P18860_1_369             ----------------------------YTPPPDFNQCLAS-PNGKFIS-
UniRef90_G1KPB5_1_370             ----------------------------CTPPPDFNQCLAN-PGGKYIS-
UniRef90_K7FWT6_1_369             ----------------------------CTPPPDFNQCLVN-PNGKFIS-
UniRef90_F1QL21_1_400             DLAENIQPR---------------LSQTCTPPPDFNQCLRSNQGPTSPPH
UniRef90_UPI0002A497DC_1_408      HLSMSPLP-------------------PSSPPPDFNQCVTGSSPFLSLP-
Input_protein_seq                 SSIQKAKG-------------------YQLLEEEKIV--SHYFPLTEVGM
UniRef90_UPI00027FC10C_1_397      SSIQKAKG-------------------YQLLEEEKIV--SHYFPLTEVGM
UniRef90_L9JGQ0_1_441             SSIQKAKKD------------------YQLLEEEKIV--SHYFPLTEVGM
UniRef90_P28236_1_440             SSIQKAKG-------------------YQLLEEEKIV--SHYFPLTEVGM
UniRef90_G5BBL7_1_440             SSIQKAKG-------------------YQLLEEEKIV--SHYFPLTEVGM
UniRef90_M3XED1_1_440             SSIQKAKG-------------------YQLLEEEKIV--SHYFPLTEVGM
UniRef90_L5K685_1_440             SSIQKAKG-------------------YQLLEEEKIV--SHYFPLTEVGM
UniRef90_F6VG96_1_441             SSIQKAKG-------------------YQLLEEEKIV--SHYFPLAEVGM
UniRef90_P55917_1_440             SSIQKAKG-------------------YQLLEEEKIV--SHYFPLTEVGM
UniRef90_UPI000329268E_1_442      SSIQKAKG-------------------YQLLEEEKIV--SHYFPLTEVGM
UniRef90_G3UBH4_1_410             SSIQKAKG-------------------YQLLEEEKIV--SHYFPLTEVGM
UniRef90_I3N7I6_1_431             SSIQKAKG-------------------YQLLEEEKIV--SHYFPLTEVGL
UniRef90_G3X210_1_429             SSIQKAKG-------------------YQLLEEEKIV--SHYFPLTEVGV
UniRef90_F7BI06_1_372             ------------------------------------------------GV
UniRef90_UPI000333FA9E_1_424      SSIQKAKG-------------------YQLLEEEKIV--SRYFPLTEVGM
UniRef90_K7EY71_1_406             SSIQKVKG-------------------YKLLEEEKPV--SRYLPLTKVG-
UniRef90_M7BCA4_1_394             SSIQKVKG-------------------YRLLEEEKPV--SHYLPLTEVG-
UniRef90_P36381_1_400             SSIPKAKG-------------------YKLLEEEKPV--SHYFPLTEVG-
UniRef90_H2MUH4_1_428             SSLQRAKG-------------------YKLLEEEKAPPMCHLYPLAEVG-
UniRef90_UPI00022B4A0D_1_396      PSLQRAKG-------------------YRLLEEEKAPPMTHLYPLAEVG-
UniRef90_H3D8V1_1_361             PSMQRVKG-------------------YRLLEEEKAPPITHLYPLAEVG-
UniRef90_G3NHJ7_1_420             PALQRAKG-------------------YRLLEEEKAP-MTQLYPLTEVG-
UniRef90_Q503J6_1_391             SSIQKAKG-------------------YKLLEEDKST--SHFFPLTEVGG
UniRef90_F6QCF2_1_438             SSIPKSKG-------------------YKLLDEEKVV--SRFYPMTEVG-
UniRef90_UPI00034FAB61_1_453      SSIPKSKG-------------------YKLLDEEKVV--SRFYPMTEVG-
UniRef90_UPI0001DE8510_1_439      SSIPKSKG-------------------YKLLDEEKVV--SRFYPMTEVG-
UniRef90_H3A837_1_370             SSIQKAKG-------------------YKLLEEDKPV--SHFYPLTEVG-
UniRef90_G1KPB7_1_391             SSIQKVKG-------------------YKLLEEEKPA--FHYFPLTEVG-
UniRef90_B0S6Z4_1_382             TPVQKAKG-------------------YRRLEEEKKDEVAHIYPLAEVG-
UniRef90_G1PZ72_1_426             ASVQKAQG-------------------YQLLEEEKIV--SHYFPLAEAGL
UniRef90_UPI000155FB82_1_448      SAPA---------------------TITIGFPPYYTHSASSLGQAMATGY
UniRef90_F6U7L2_1_428             SAPA---------------------TITIGFPPYYTHSASSLGQAMATGY
UniRef90_UPI0002C6341D_1_440      SAPP---------------------TITIGFPPSYTHSASSLGQAMATGY
UniRef90_M3Z8M0_1_445             SAPP---------------------TVTIGFPPYYAHSASSLGQATGAGY
UniRef90_G1Q1M8_1_411             --PQ---------------------TLVLGVPFYYTLSVSSLGWAT----
UniRef90_G1PYG2_1_394             --PQ---------------------TLVLGVPFYYTLSVSSLGWAT----
UniRef90_L5L5Q1_1_430             SAPA---------------------TGTGACPPYYTHSASSLGQAMSRAY
UniRef90_UPI0002BCF55A_1_410      SNPP---------------------ALTIGFPPSYAPSASSLGQASAPGY
UniRef90_P41987_1_407             SAPP---------------------AVTIGFPPYYAPSASSLGQASAPGY
UniRef90_J9JHI9_1_433             SEAP---------------------AVTIGFPPFSAPSASSPGQATAAGY
UniRef90_Q9Y6H8_1_435             SRPP---------------------AVAIGFPPYYAHTAAPLGQARAVGY
UniRef90_G3QHB2_1_399             SRPP---------------------AVAIGFPPYYAHTAAPLGQARAVGY
```

UniRef90_F7B1T8_1_432	SRP----------------------AAALGFPPYYAHT-APAGQARALGY
UniRef90_L9L2D3_1_460	SGPP---------------------TVTIGFPPYYTHSASNLGQVRAPGY
UniRef90_Q64448_1_417	SGPP---------------------SVSIGFPPYYTHPACPTVQAKAIGF
UniRef90_G3H8J5_1_417	SGLP---------------------SASMGFPPFYTHPACPPVQARATGF
UniRef90_UPI0003333DA4_1_405	PGPP---------------------SVSTGFLPYCTHPGCSPGQSRATKY
UniRef90_I3NC37_1_417	--PP---------------------TVTIGFPPLYTHYASPLGQAGEAGY
UniRef90_H0XD37_1_430	SGPA---------------------AVTIGFPPYYTQAPSSLGQARPAGY
UniRef90_G1U3S9_1_407	PGPP---------------------SVAIGFPPSYAH-AAALPQ--ERGK
UniRef90_UPI00032AEFC1_1_413	PGPP---------------------AVTIGFPPSYAHAAAALPQGPARAS
UniRef90_UPI00034F30A8_1_402	PGPA---------------------TVAIGFPPYYAHSASAPGQAAPKGY
UniRef90_UPI0003340A67_1_370	-------------------------AVPLGVSPCCVHTS--------QGH
UniRef90_UPI0002234D0E_1_449	SVPP---------------------TISIGFPPYYTHSASSLGQTMATGY
UniRef90_G3TTN5_1_429	SVPP---------------------TISIGFPPYYTHSASSLGQTMATGY
UniRef90_UPI0002C457ED_1_447	SAPP---------------------TIPIGFP-YYTHSASSPGQTMATGY
UniRef90_UPI000333D50E_1_427	STPT---------------------PAPLGFPPYYTHSASSLGQTVATGY
UniRef90_UPI0003343FBC_1_423	SALP---------------------AVTVGFPPCSTHSASSLAQT---AY
UniRef90_F7G5V2_1_446	SAPP-----------------------AVGFPPYYTQSASSLGQATTTGY
UniRef90_G3VY19_1_428	SAPP-----------------------TVGFPPYYTQSASSLGQATTAGY
UniRef90_UPI000155BBAC_1_459	APPPP--------------------TVTIGFPPFYSQSSSSLRQATPSGY
UniRef90_F7ETY8_1_425	APPPP--------------------TVTIGFPPFYSQSSSSLRQATPSG-
UniRef90_I3KXW1_1_474	PSSPSYP----------------PNYTD------VTAGSGAFLPPMGPAA
UniRef90_UPI00022B3AE8_1_449	PSSPSYP----------------PNYTD------VTAGSGAFLPPMGPAA
UniRef90_UPI0002A48057_1_447	PSSLSYP----------------PNYTD------VTAGSGAFLPPMDPAA
UniRef90_H2MCD5_1_421	PSSLSYP----------------PNYTD------VTAGSGAFLPPMDPAA
UniRef90_H2UTF3_1_420	PSS--YP----------------PNYTD------VTAGSGAFLQPVAPPA
UniRef90_UPI000298F1F6_1_460	PSS--YP----------------PNYTD------VTAGSGAFLQPVAPPA
UniRef90_H3DE08_1_376	AGR--------------------AFGRR------VQDGR----PPAEPAS
UniRef90_Q8JFD2_1_434	PPTLSYP----------------PDYTE------VAVAGGAFLQPVS---
UniRef90_H2T4Y1_1_438	PSALECL----------------STYSSMNMAGRVAEEGGTYSPPEASLA
UniRef90_H2T4Y2_1_428	PS-----------------------------ARRVAEEGGTYSPPEASLA
UniRef90_H3BZL0_1_378	---------------------------------SGGNEGRSYSPPEASLA
UniRef90_I3K1N0_1_478	PLVLDCL----------------PAFGSVSVMGDGAAEGGAYSPPESFPT
UniRef90_H2LFL5_1_470	PSALNRL----------------P-------EYANGNANASFMGDRAAEE
UniRef90_M7BWJ7_1_486	PSPAAFP---TVSPALPDSHTITPLLSSQTVPPYYAEATARAQP-AATTF
UniRef90_K7F163_1_492	PSPAVLP---AVPPALSDSRAITPLLSSQTVPPYYAEATARAQP-ATAVS
UniRef90_H0ZM32_1_512	PAPPVVVTTATPPPVLPDTRAVTPLLAPVTMASYYATGAPRPRPPSNTTS
UniRef90_H3AH16_1_396	---------------------------SLHYPPFYAEATSGGHHGYPGAP
UniRef90_B0BM18_1_388	------------------------------YYTDVAAALPVLHHAYARFS
UniRef90_Q4S8R0_1_345	PSYN----------------------------YYTGHPAVPEPFPTDSKY
UniRef90_I3KYH7_1_341	PNYN----------------------------YFGGHSAVPEPFPTDSKY
UniRef90_H2M0G7_1_345	PNYT----------------------------YLTSHTLSHEPFSADSKF
UniRef90_H3A471_1_360	QHYP----------------------------FFPSHGRPQPYNPSLAAY
UniRef90_Q6GLA5_1_353	PHYP----------------------------HYSPTEK-GSTFSPTLAF
UniRef90_UPI0002A4A1D5_1_346	PSYDD---------------------------YFSGHCQVQPAFPPIPSY
UniRef90_S4RXA6_1_456	HSVKTSS-------------------QPISYTYYQPPTDERNYDPPSFRT
UniRef90_P16863_1_360	CSSPT---------------------APMSPPGYKLVTGERN--PSSCRN
UniRef90_H2LU51_1_404	CVGTSVGR-------------------KTTIPTAPSG----YTLLLEKQG
UniRef90_I3KYL3_1_401	CMGTTVGR-------------------KSTIPTAPSG----YTLLLEKQG
UniRef90_G3NC44_1_402	CVGTSVGR-------------------KTTIPTAPCG----YTLLLEKQG
UniRef90_H2SSL2_1_392	CAGTSVAG-------------------KTTIPTAPCG----YTLLMEKQG
UniRef90_UPI0002C46B7E_1_433	---TSANS-------------------LKQLPSASD-----YHLLVEKKT
UniRef90_G3ULF1_1_432	---VSANS-------------------LKGLPSAPD-----YNLLVEKKT
UniRef90_C9IYK1_1_407	---TSANS-------------------LKRLPSAPD-----YNLLVEKQT
UniRef90_H0Y0G7_1_436	---TAANS-------------------LKRFPSAPN-----YNLLVEKQT
UniRef90_L9JD22_1_408	---TSANS-------------------LKRLPSAPD-----YNLVVEKQT
UniRef90_F1N617_1_436	---TSANS-------------------LKRLSSVPD-----YSLLVDKQT
UniRef90_F1SV25_1_385	---TSANS-------------------LKRLSSAPD-----YSLLVEKQT
UniRef90_L5JUH5_1_307	---TSANS-------------------LKQLSSAPD-----YNLLVEKQT
UniRef90_UPI00020369CA_1_441	SLGISATP-------------------QKTLPSVLSG----YAFLMEKQT
UniRef90_R4GLX1_1_405	SLGISATP-------------------QKTLPSVLSG----YAFLMEKQT
UniRef90_UPI00035A19E6_1_466	SLEIPTTP-------------------QKTLPCALSS----HTFLMEKQT
UniRef90_F6WA84_1_463	CLGTATNP-------------------PKTLPSAPIG----YTVLREKQM
UniRef90_G1SG18_1_430	MICSSLPD-------------------RISLLQANN-----QQQGIRINV
UniRef90_UPI00032B0E64_1_413	MICSSLPE-------------------RISLFQANN-----QKQGILVNV
UniRef90_UPI0002C65D9D_1_412	MICSPLPE-------------------KISLLQANN-----QQQVIQLNV
UniRef90_F7CF28_1_383	MICSSLPE-------------------KISLLQANN-----QQQVIQVNV
UniRef90_Q969M2_1_435	MICSSLPE-------------------RISPLQANN-----QQQVIRVNV
UniRef90_L9JVZ3_1_406	MICSSLPE-------------------RMSLLQANN-----QQQVIQVNV
UniRef90_I3M407_1_414	TICSSLPE-------------------RISPLQTNN-----QQKVIQVNV
UniRef90_L5JMB7_1_435	MICSPLLE-------------------RTPVLQANS-----QQQIILVNV
UniRef90_L8J083_1_417	MTCSPFPE-------------------KISLLQANN-----QQQVIRVNV
UniRef90_UPI0002BCCFF6_1_421	MNCSPFPE-------------------GVSLLQASN-----QQQVFRVNV
UniRef90_F1RY20_1_421	MISPPFPE-------------------RISLLQANN-----QQQVIQVNV
UniRef90_UPI0002C59166_1_412	MICSPLPK-------------------RIPLLQANN-----QQQVIRVNV
UniRef90_G3TBX7_1_436	MICSSLPD-------------------RPSLLQANN-----RQKIIRVNM
UniRef90_G3GSS7_1_405	MACSSLPE-------------------KFSLLQANN-----KQQVIRVNI

```
UniRef90_UPI0003316158_1_413      MICSSLHE-------------------RSSLLQANN-----QQHPVQASA
UniRef90_G3WUN4_1_443             VTHSLLHE-------------------NIALLQPVQPIWPEQQQVIGIDN
UniRef90_F6W379_1_264             --------------------------------------------------
UniRef90_M7BL54_1_431             CIMSNSSP-------------------QSSYKLLPN-----QQGGLPVYL
UniRef90_UPI0002B44362_1_276      ---------------------------------------------QPLWP
UniRef90_UPI000298933C_1_429      VLTNSSPQ-------------------RLVQLTQMTCSALPDTHGETLAI
UniRef90_H3CDZ2_1_426             LLASSSPQ-------------------RLVQLTQVTCSALPNTN----AT
UniRef90_I3IZH7_1_440             VLSNSSPQ-------------------RLIQLTQMTCSAMPETQVESQPL
UniRef90_E7F3P4_1_389             FLTNSSPQ-------------------KQLHLTHTSLAMAPDGQMVPLPI


UniRef90_UPI0002C691BE_1_358      PFSNK-------------------MASQQNTDNLAT--------------
UniRef90_F7BKC0_1_358             PFSNK-------------------MASQQNIDNLAT--------------
UniRef90_Q0VCR2_1_359             PFSNK-------------------MASQQNTDNLST--------------
UniRef90_G3TUX5_1_358             PFSNK-------------------MASQQNTDNLAT--------------
UniRef90_L9JKN2_1_358             PFSNN-------------------TASQQNTDNLAT--------------
UniRef90_P36382_1_358             PFSNN-------------------MASQQNTDNLVT--------------
UniRef90_UPI0003315E33_1_350      PFSNK-------------------MASQQNTDNLAT--------------
UniRef90_UPI000333FB64_1_358      PFSNK-------------------LASQQNTDNLAT--------------
UniRef90_UPI000334540D_1_357      PFSNK-------------------MASQQNTHNLAT--------------
UniRef90_L5K3R8_1_358             PFSNQ-------------------VASQQNTDNLAT--------------
UniRef90_G1TQ37_1_358             TFSTN-------------------TASQQNTDNLAT--------------
UniRef90_UPI0001776F40_1_352      RFSNN-------------------MASQQNTDNLAT--------------
UniRef90_G3H2Q9_1_358             DFSNN-------------------MGSRKNPDTLAT--------------
UniRef90_Q01231_1_358             DFSNN-------------------MGSRKNPDALAT--------------
UniRef90_G5BBL6_1_357             PLKNN-------------------MASQQNTDNMAT--------------
UniRef90_H0WCZ7_1_358             PFSNN-------------------TVSQQNTDNLAT--------------
UniRef90_G3X1A3_1_358             HFSNN-------------------LASRQNTDNLAT--------------
UniRef90_H0ZTI1_1_370             PFSNK-------------------MASQQNTANFAT--------------
UniRef90_P18860_1_369             PFSNK-------------------MASQQNTANFAT--------------
UniRef90_G1KPB5_1_370             PFSNK-------------------MASQQNTANFAT--------------
UniRef90_K7FWT6_1_369             PFSNK-------------------MASQQNTANFAT--------------
UniRef90_F1QL21_1_400             LHSHH-------------------LHHIHQTCQPFT--NH----------
UniRef90_UPI0002A497DC_1_408      FPSHL-------------------LVHQQNSENTVTEKNK----------
Input_protein_seq                 VETSP------------------LPAKPFNQFEEKI--------------
UniRef90_UPI00027FC10C_1_397      VETTP------------------VSAKPFNQFEEKI--------------
UniRef90_L9JGQ0_1_441             VETSP------------------LSAKPFSQFAEKM--------------
UniRef90_P28236_1_440             VETSP------------------LSAKPFSQFEEKI--------------
UniRef90_G5BBL7_1_440             VQTSP------------------LSAKPFSQFEEKT--------------
UniRef90_M3XED1_1_440             VETSP------------------LSAKPFAQFEEKM--------------
UniRef90_L5K685_1_440             VETSP------------------LSAKPFGQFEEKM--------------
UniRef90_F6VG96_1_441             VETSP------------------LSAKPFSQFEEKM--------------
UniRef90_P55917_1_440             VEASP------------------LSAKPFSQFEEKV--------------
UniRef90_UPI000329268E_1_442      VETTP------------------LSAKPFHQFEEKI--------------
UniRef90_G3UBH4_1_410             VETSP------------------LSAKPFSQFEEKI--------------
UniRef90_I3N7I6_1_431             VETSP------------------LAAQPFGQFEEKV--------------
UniRef90_G3X210_1_429             VETRP------------------LAATPFSRFEEKI--------------
UniRef90_F7BI06_1_372             VETRP------------------LAATPFSRFEEKI--------------
UniRef90_UPI000333FA9E_1_424      VESSP------------------LADKPFSQFEEKM--------------
UniRef90_K7EY71_1_406             VETAP------------------PPS-SFNDLDEKL--------------
UniRef90_M7BCA4_1_394             VEAGP------------------PPS-SFKDLDEKL--------------
UniRef90_P36381_1_400             VEPSP------------------LPS-AFNEFEEKI--------------
UniRef90_H2MUH4_1_428             MEAGR------------------GAS-PFHGLEEKG--------------
UniRef90_UPI00022B4A0D_1_396      MEAGR------------------GAP-PFQGLEEKV--------------
UniRef90_H3D8V1_1_361             MEAGR------------------GSP-PFQGLEEKP--------------
UniRef90_G3NHJ7_1_420             MEAGR------------------GPP-PFLGLEEKA--------------
UniRef90_Q503J6_1_391             MEAGR------------------LPA-SYEPFEEKSD-------------
UniRef90_F6QCF2_1_438             LEASP------------------LPA-HFNGYDK----------------
UniRef90_UPI00034FAB61_1_453      LEASP------------------LPA-HFNGYDK----------------
UniRef90_UPI0001DE8510_1_439      LEASP------------------LPA-HFNGYDK----------------
UniRef90_H3A837_1_370             VETTQ------------------LPA-PFAGFEEKE--------------
UniRef90_G1KPB7_1_391             AETTP------------------VPP-SLNEFEEKM--------------
UniRef90_B0S6Z4_1_382             MEEGQ------------------FFLPQLEKEQKSSQEAI----------
UniRef90_G1PZ72_1_426             GEASP------------------RSAQPFRRLEERR--------------
UniRef90_UPI000155FB82_1_448      PGAPP-------------------PPTDFKMVALS-----------EAHR
UniRef90_F6U7L2_1_428             PGAPP-------------------PPTDFKMVALS-----------EAHR
UniRef90_UPI0002C6341D_1_440      PGAPP-------------------PATDFKMVALS-----------EAHG
UniRef90_M3Z8M0_1_445             PGAPP-------------------PATDFDMAALS-----------EAPG
UniRef90_G1Q1M8_1_411             --APP-------------------PATDFNMAGLS-----------QTQG
UniRef90_G1PYG2_1_394             --APP-------------------PATDFNMAALS-----------QTQG
UniRef90_L5L5Q1_1_430             PGAPL-------------------PATDVTKEALS-----------EAQG
UniRef90_UPI0002BCF55A_1_410      PEPPP-------------------P------AALP-----------GTPG
UniRef90_P41987_1_407             PEPPP-------------------P------AALP-----------GTPG
UniRef90_J9JHI9_1_433             PGAPP-------------------L------AADF-----------HPAA
UniRef90_Q9Y6H8_1_435             PGAPP-------------------PAADFKLLALT-----------EARG
UniRef90_G3QHB2_1_399             PGAPP-------------------PAADFRMLALT-----------EARG
```

UniRef90_F7B1T8_1_432	PGAPP-------------------PAADFKLLALT-----------EARG
UniRef90_L9L2D3_1_460	PGAPPG------------------PAADFKVGALT-----------EAQG
UniRef90_Q64448_1_417	PGAPL-------------------SPADFTVVTLN-----------DAQG
UniRef90_G3H8J5_1_417	PEAPP-------------------PPADFTVVTLN-----------DAQD
UniRef90_UPI0003333DA4_1_405	PGTPP-------------------PAADFTVVSLK-----------EAQG
UniRef90_I3NC37_1_417	PGAPP-------------------PAADFKMVALN-----------ESQG
UniRef90_H0XD37_1_430	PGAPPP------------------PAADFKVVALS-----------EVPE
UniRef90_G1U3S9_1_407	G-QAL-------------------PTTEQNWAPTP-------------VA
UniRef90_UPI00032AEFC1_1_413	P-PAG-------------------DSTEVALVAEPEKGRL------LPTS
UniRef90_UPI00034F30A8_1_402	PGAPL-------------------PTADFRTVAQG-----------EAHG
UniRef90_UPI0003340A67_1_370	SGTPL-------------------PATDLGLEAPG-----------EACV
UniRef90_UPI0002234D0E_1_449	PGAPP-------------------PPADFKMVPLS-----------EEQG
UniRef90_G3TTN5_1_429	PGAPP-------------------PPADFKMVPLS-----------EEQG
UniRef90_UPI0002C457ED_1_447	LGAPP-------------------PPTDFKMVALS-----------EEQG
UniRef90_UPI000333D50E_1_427	PGAPP-------------------PPTDFKMVALA-----------EERG
UniRef90_UPI0003343FBC_1_423	PGTTL-------------------PATNFKTVALA-----------EVHG
UniRef90_F7G5V2_1_446	PGAPP-------------------QPTEFRMVTLP-----------EERS
UniRef90_G3VY19_1_428	PGAPP-------------------QPTEFRMVTLP-----------EEHS
UniRef90_UPI000155BBAC_1_459	PGAPG-------------------PPTEFKMAGLP-----------KERS
UniRef90_F7ETY8_1_425	----------------------------FKMAGLP-----------KERS
UniRef90_I3KXW1_1_474	VPSA-----------------AEFKMDDLQQEESLQSSP-----------
UniRef90_UPI00022B3AE8_1_449	VPSA-----------------AEFKMDDLQQEESLQSSP-----------
UniRef90_UPI0002A48057_1_447	LSSA-----------------EE--MEALHQEESIHQAPP----------
UniRef90_H2MCD5_1_421	LSSA-----------------EE--MEALHQEESIHQAPP----------
UniRef90_H2UTF3_1_420	VPST-----------------TEFKTDDLQREPPRHQPS-----------
UniRef90_UPI000298F1F6_1_460	VPST-----------------TEFKTDDLQREPPRHQPS-----------
UniRef90_H3DE08_1_376	PP--
UniRef90_Q8JFD2_1_434	APST-----------------AEFKMDPLREELEE---------------
UniRef90_H2T4Y1_1_438	VMSS-------PTGLKMD--GTVFHPDDFLLEALPPSFCS----------
UniRef90_H2T4Y2_1_428	VMSS-------PTGLKMD--GTVFHPDDFLLEALPPSFCS----------
UniRef90_H3BZL0_1_378	VMSS-------PASLKMD--GSAFHPDDLLLEALPASFCG----------
UniRef90_I3K1N0_1_478	VTSLNAATTPVPAGMKMD--STMFHPDDFLLDAA--SFYS----------
UniRef90_H2LFL5_1_470	GASS-------PAVIFNPNMATTPTPSELNLEVRSPSFST----------
UniRef90_M7BWJ7_1_486	LADYP---------------GAPPFSEEQHNTATPTPTSIPIPTPTSIPT
UniRef90_K7F163_1_492	LAGYS---------------GALPFAEELHKTATPIPASTPIPTPTLVPT
UniRef90_H0ZM32_1_512	MASYP---------------AAPQVPEERHHAVTPTPISTPVTIPTPIPT
UniRef90_H3AH16_1_396	AADFK---------------AMPPPAEEPHKPS-----------------
UniRef90_B0BM18_1_388	APDFK---------------ITSVPEEALESPS-----------------
UniRef90_Q4S8R0_1_345	SVT----------------------EPGSAYSPYS---------------
UniRef90_I3KYH7_1_341	GMP----------------------EPNSAYSPYN---------------
UniRef90_H2M0G7_1_345	NVA----------------------EPNSAYNPYN---------------
UniRef90_H3A471_1_360	PLPSM-------------------NENNSSFHPYN---------------
UniRef90_Q6GLA5_1_353	PLSP---------------------SKTTSNVMDS---------------
UniRef90_UPI0002A4A1D5_1_346	DLSPLS------------------KSTDSSFHPYH---------------
UniRef90_S4RXA6_1_456	ATRDLVAGGR----------LVAQGVDGQNWANWE------------EEE
UniRef90_P16863_1_360	YNKQASE---------------------QNWANYS------------AEQ
UniRef90_H2LU51_1_404	NGPN-------------------YPLLSASSAFVPIQ-------------
UniRef90_I3KYL3_1_401	NGPN-------------------YPLLNASSAFVPIQ-------------
UniRef90_G3NC44_1_402	NGPA-------------------YPLLNASSAFVPIK-------------
UniRef90_H2SSL2_1_392	NGPN-------------------YSLLNASSAFIPIQ-------------
UniRef90_UPI0002C46B7E_1_433	HTTV-------------------YPSLNSSSALQVDP-------------
UniRef90_G3ULF1_1_432	HKTV-------------------YPSFNSSSALQVDP-------------
UniRef90_C9IYK1_1_407	HTAV-------------------YPSLNSSSVFQPNP-------------
UniRef90_H0Y0G7_1_436	HTAE-------------------YPSLNSPSVFQANL-------------
UniRef90_L9JD22_1_408	HPAV-------------------YPSLNSSSAFQADP-------------
UniRef90_F1N617_1_436	HTAG-------------------YPSLN-PSAFQTDP-------------
UniRef90_F1SV25_1_385	HRAV-------------------YPSLN-SSVYQTDP-------------
UniRef90_L5JUH5_1_307	HTAV-------------------YPSLN-LSAFQADP-------------
UniRef90_UPI00020369CA_1_441	DTAI-------------------YPVLNSAPMFQSIQ-------------
UniRef90_R4GLX1_1_405	DTAI-------------------YPVLNSAPVFQPVQ-------------
UniRef90_UPI00035A19E6_1_466	DTML-------------------YPVLNSPSVFQSVQ-------------
UniRef90_F6WA84_1_463	NPTV-------------------YPGFSSPSVLQALQ-------------
UniRef90_G1SG18_1_430	PKSK--------------------TMWQIPQPRQPEA-------------
UniRef90_UPI00032B0E64_1_413	PKSK--------------------AMWQIPQPRQSEE-------------
UniRef90_UPI0002C65D9D_1_412	PKSK--------------------TMWQIQQPRQLEV-------------
UniRef90_F7CF28_1_383	PKSK--------------------TMWQIQQPRQLEV-------------
UniRef90_Q969M2_1_435	PKSK--------------------TMWQIPQPRQLEV-------------
UniRef90_L9JVZ3_1_406	PKSK--------------------SMWQIPQPRRLGI-------------
UniRef90_I3M407_1_414	PKSK--------------------SIWQIPQPPQLDV-------------
UniRef90_L5JMB7_1_435	PKSK--------------------TSWQIPQPRQLEV-------------
UniRef90_L8J083_1_417	PNSK--------------------TTWHIPQARQLDV-------------
UniRef90_UPI0002BCCFF6_1_421	PNSK--------------------TTWQIPQPRQLEV-------------
UniRef90_F1RY20_1_421	PKSK--------------------TTWQPPQPRQFEV-------------
UniRef90_UPI0002C59166_1_412	PKSK--------------------TMWQSSQPMQLGV-------------
UniRef90_G3TBX7_1_436	PKSE--------------------TIWQIPQAKRLEV-------------
UniRef90_G3GSS7_1_405	PRSK--------------------SMWQIPHPRQHEV-------------

```
UniRef90_UPI0003316158_1_413      PKFN--------------------TMRQIPQPRQLEV-------------
UniRef90_G3WUN4_1_443             TEHK--------------------PAWQPNQHNQSEG-------------
UniRef90_F6W379_1_264             --------------------------------------------------
UniRef90_M7BL54_1_431             PPVQGYK-------------VLQAPADHCQQAVGMGA-------------
UniRef90_UPI0002B44362_1_276      P-------------------------------------------------
UniRef90_UPI000298933C_1_429      NVSHQ---------------NQEGSGA-TNQHPSHIG-------------
UniRef90_H3CDZ2_1_426             SLSHQ---------------NQEGSGN-ANQHPSHAC-------------
UniRef90_I3IZH7_1_440             NLALV---------------APQNLEG-HNISCRPSQ-------------
UniRef90_E7F3P4_1_389             YMQTAGHVVS--------NINPNGSVQPLRQDRLPSQ-------------


UniRef90_UPI0002C691BE_1_358      -------------EQVQDQEQIPGEG-FIHIRYAQ---------------
UniRef90_F7BKC0_1_358             -------------EQVRDQEQIPGEG-FIHIRYAQ---------------
UniRef90_Q0VCR2_1_359             -------------EQVRSQEQIQREG-FIHIRYAQ---------------
UniRef90_G3TUX5_1_358             -------------EQVRGQEEIPGEG-FINIRYAQ---------------
UniRef90_L9JKN2_1_358             -------------EQVQGQEQIPMEG-FIHVRYGQ---------------
UniRef90_P36382_1_358             -------------EQVRGQEQTPGEG-FIQVRYGQ---------------
UniRef90_UPI0003315E33_1_350      -------------EQVRGQDPLSRDG-FIHIHYDQ---------------
UniRef90_UPI000333FB64_1_358      -------------EQVRGHEEMPGEG-FTHLRYAQ---------------
UniRef90_UPI000334540D_1_357      -------------EQVRGQEQAPGDG-FIHVRYAQ---------------
UniRef90_L5K3R8_1_358             -------------EQVRGQEQVPGEG-FIHVHYSQ---------------
UniRef90_G1TQ37_1_358             -------------ERVRGREQVPGEG-FIHIRYDQ---------------
UniRef90_UPI0001776F40_1_352      -------------EQVQGQE--PG-----HIHYDQ---------------
UniRef90_G3H2Q9_1_358             -------------EEMPDQELISRDG-FIHMHYGQ---------------
UniRef90_Q01231_1_358             -------------GEVPNQEQIPGEG-FIHMHYSQ---------------
UniRef90_G5BBL6_1_357             -------------EQVQGHERIPGEG-FIHIHYGE---------------
UniRef90_H0WCZ7_1_358             -------------EQVRGQERIPGED-FIHIHYGE---------------
UniRef90_G3X1A3_1_358             -------------EQVQGREEAAGRA-FLHMQYAE---------------
UniRef90_H0ZTI1_1_370             -------------ERVHGQEDAAGEGPFIKSSYVE---------------
UniRef90_P18860_1_369             -------------ERVHSQEDAAGEGPFMKSSYME---------------
UniRef90_G1KPB5_1_370             -------------ERVQSQDDIIGEGPFIQVNYTQ---------------
UniRef90_K7FWT6_1_369             -------------ERVHSQEDAAGEGQFIQINYAQ---------------
UniRef90_F1QL21_1_400             LAHQQNSVNMAAERHHHSHDGLEPAVDFLQMHYGSPEARVR---------
UniRef90_UPI0002A497DC_1_408      IVAAVEEANFLQMSYYSPEWQSAQNGQIQDGVYLKSDSKCY---------
Input_protein_seq                 --------------STGPLGDLSRGYQETLPSYAQVGAQEVEGE------
UniRef90_UPI00027FC10C_1_397      --------------STGPLGDLSRGYEETLPSYAQVGVQEVEGE------
UniRef90_L9JGQ0_1_441             --------------STGPLADLSRGYQETLPSYAQVGAQEVEAE------
UniRef90_P28236_1_440             --------------GTGPLADMSRSYQETLPSYAQVGVQEVERE------
UniRef90_G5BBL7_1_440             --------------GTGPLADISRSYQETLPSYAQVGAQEVEGE------
UniRef90_M3XED1_1_440             --------------GPGPLGDLSRAYQETLPSYAQVGAQEGESG------
UniRef90_L5K685_1_440             --------------GTGPLGDLSRAYQETLPSYAQVGAQEVEAE------
UniRef90_F6VG96_1_441             --------------GTGPLGDLSRAYQETLPSYAQVGAQEVEGEG-----
UniRef90_P55917_1_440             --------------GPGPLGDLSRAYQETLPSYAQVGAQEGVEE------
UniRef90_UPI000329268E_1_442      --------------GTGPLGDLSRAYQETLPSYAQVGAQEVEGE------
UniRef90_G3UBH4_1_410             --------------GTGPLGDLSQVYQETLPSYAQVGALTTDGQ------
UniRef90_I3N7I6_1_431             --------------GAGPLADLARGYPETLPSYAQVGAQEGAGE------
UniRef90_G3X210_1_429             --------------GTGPLGDLSRAYTETLPSYAQVGEQEVEAER-----
UniRef90_F7BI06_1_372             --------------STGPLGDLSRAYTETLPSYAQVGEQEVEAER-----
UniRef90_UPI000333FA9E_1_424      --------------TAAPLGDLSQVCRAKLPSYAPVEVEDVEQP------
UniRef90_K7EY71_1_406             --------------GMGPVEDLSRTFDERLPSYAQAKEQKE---------
UniRef90_M7BCA4_1_394             --------------GMGPLEDLSRAFDERLPSYAQAEEQKE---------
UniRef90_P36381_1_400             --------------GMGPLEDLSRAFDERLPSYAQAKEPEE---------
UniRef90_H2MUH4_1_428             -------------EEVLPMEDITKVYDETLPSYAQTTATV----------
UniRef90_UPI00022B4A0D_1_396      -------------EEVLPMEDISKVYDETLPSYAQTTETG----------
UniRef90_H3D8V1_1_361             -------------EEVLPMEDISKVYDETLPSYTQTTETG----------
UniRef90_G3NHJ7_1_420             -------------EEVLPMGGISKAYDETLPSYAQTTETA----------
UniRef90_Q503J6_1_391             -------------EAMAPKKDMSKMYDETLPSYAQTTVIGPSAS------
UniRef90_F6QCF2_1_438             -------------GSTGLLEDISKGYDETLPSYRQAEELDG---------
UniRef90_UPI00034FAB61_1_453      -------------GSTGLLEDISKGYDETLPSYRQAEELDG---------
UniRef90_UPI0001DE8510_1_439      -------------GSTGLLEDISKGYDETLPSYRQAEELDG---------
UniRef90_H3A837_1_370             -------------NATGPLEDISKVYDETLPSYGQMTEQE----------
UniRef90_G1KPB7_1_391             --------------SMEHLEDIPSAFEEQLPSYVQRDEQEE---------
UniRef90_B0S6Z4_1_382             -------------LPTAPPVEETIICDETQPSFLQVTETLP---------
UniRef90_G1PZ72_1_426             --------------GAGLPGGLGGADPEALPSYAQVGAPGCELG------
UniRef90_UPI000155FB82_1_448      NGHPANFCNGNH-HLLMTEQNWANQEAEQQRSVRKASLPASTSASLTPPG
UniRef90_F6U7L2_1_428             NGHPANFCNGNH-HLLMTEQNWANQEAEQQ--------------------
UniRef90_UPI0002C6341D_1_440      KGHAAKFYNGNH-HLLMTEQNWANQEAEQQSSARKASPPVSASASLTPPG
UniRef90_M3Z8M0_1_445             KDHPAKFYNGNH-HLLMTEQNWANQEAEQQTSARRASLPASGSASLSPTG
UniRef90_G1Q1M8_1_411             KGHPDKFYNSNH-HLIMTEQNWANQEPGQQILP-----------------
UniRef90_G1PYG2_1_394             KGHPDKFYNSNH-HLIMTEQNWANQEPGQQAAP-----------------
UniRef90_L5L5Q1_1_430             KGHPDKFYDSSQ-HLLTAEQNWATQEAEQQLSVRKATPPPLS------PS
UniRef90_UPI0002BCF55A_1_410      KGHPVGSPGGHQ-HLLATAPNWAGREAEQQTSAGKASPP-----------
UniRef90_P41987_1_407             --TPGG--GGNQ-GLRARAQNWANREAEPQTSSRKASPP-----------
UniRef90_J9JHI9_1_433             --LPEAR-ARDQ-HLLMTEQNWANQEAEQHTSARKASG------------
UniRef90_Q9Y6H8_1_435             KGQSAKLYNGHH-HLLMTEQNWANQAAERQPPALKAYPAA----------
UniRef90_G3QHB2_1_399             KGQSAKLYNGHH-HLLMTEQNWANQAAERQPPALKAYAAA----------
```

```
UniRef90_F7B1T8_1_432            RCPSAQLYHGHH-HLPLTEQNWAGRAAEQQRHELRAYPAA----------
UniRef90_L9L2D3_1_460            QGHPTKLYNG-H-HLLMTEQNWANQAAEQQSPARKDSPAA----------
UniRef90_Q64448_1_417            RNHPVKHCN--G-HHLTTEQNWTRQVAEQQTPASKPSSAA----------
UniRef90_G3H8J5_1_417            RGHPAKHCN--G-HHLITEQNWASRMAEQQTPASKASSAT----------
UniRef90_UPI0003333DA4_1_405     EGHPGKHN------LLMTEQNWASPAAEQQTLGSKAFPAA----------
UniRef90_I3NC37_1_417            KGHPAKLYNN-Q-HLLMTEQNWANQAAEHQTPARKASSAA----------
UniRef90_H0XD37_1_430            KGHPAKLYNGHH-HLLMPEQNWVNQAAEQLAPVLAPSLAS----------
UniRef90_G1U3S9_1_407            EGQP----------SSRKASPAASS-AAAPSPAG-SSASS----------
UniRef90_UPI00032AEFC1_1_413     EPNW----------ASPAAEPQAPSRKAAPSPGGSSSASS----------
UniRef90_UPI00034F30A8_1_402     EGHP----------LPMTAQNWASP--AWAPDAPIPPGRT----------
UniRef90_UPI0003340A67_1_370     QGHP----------LPVTAQTWVPPASAWVSTTPSPSGRA----------
UniRef90_UPI0002234D0E_1_449     KGHPARYYNSCH-HLLMTEQNWANQEAEKQASGRKPYPPVSTPAAPSPLS
UniRef90_G3TTN5_1_429            KGHPARYYNSCH-HLLMTEQNWANQEAEKQASGRKPYPPV----------
UniRef90_UPI0002C457ED_1_447     KGHPARYYN-CH-HLLMTEQNWANQEAEQQASGRKPCPLTPTPAAPCPLS
UniRef90_UPI000333D50E_1_427     KGHAARYCNRCH-HLLMTEQNWANQEAEQQASGRKPCPPVPTPVAPSFPS
UniRef90_UPI0003343FBC_1_423     KGHPAKVFSGSR-CLLGTEQSQAKQEVKQQASARSSSLLV----------
UniRef90_F7G5V2_1_446            KAAPAKYYGGNHHHILMTEQNWANQEAEQQTFERKSSPSNTTPGTPTTPS
UniRef90_G3VY19_1_428            KAAPAKYYGGNHHHILMTEQNWANQEAEQQTFERKSSPPDTAP-------
UniRef90_UPI000155BBAC_1_459     RAASAQAFSSNQ-HILTAEQNWANQEAEQQTLERKSLTAPGAP---SSPI
UniRef90_F7ETY8_1_425            RAASAQAFSSNQ-HILTAEQNWANQEAEQQTLERKSLTAPGAP---SSPI
UniRef90_I3KXW1_1_474            --SSHYYISNNNNHRLATQQNWANLATEQQTREMKATSPSSSCSATSSDN
UniRef90_UPI00022B3AE8_1_449     --SSHYYISNNNNHRLATQQNWANLATEQQTREMKATSPSSSCSATSSDN
UniRef90_UPI0002A48057_1_447     --SSHYYISNNNNHRLATQQNWANLATEQQTREMNGTSPSPSASSSTSNK
UniRef90_H2MCD5_1_421            --SSHYYISNNNNHRLATQQNWANLATEQQTREMNGTSPSPSASSSTSNK
UniRef90_H2UTF3_1_420            --ASHYYISNNNNHRLATQQNWANLATEQQTREMKATSSSSSSTNDEQR-
UniRef90_UPI000298F1F6_1_460     --ASHYYISNNNNHRLATQQNWANLATEQQTREMKATSSSSSSTNDEQQQ
UniRef90_H3DE08_1_376            ---PHYYISNNNNHRLAAQQNWANLATEQQTRSSSPGSASNAGS------
UniRef90_Q8JFD2_1_434            --SSPFYISNNNNHRLAAEQNWANLATEQQTREMNATSPCSSSSSCSSDN
UniRef90_H2T4Y1_1_438            --SNDKV---SHGQLTEVEQNWSNMALELRTLNGKNSSYP----------
UniRef90_H2T4Y2_1_428            --SNDKV---SHGQLTEVEQNWSNMALELRTLNGKNSSYP----------
UniRef90_H3BZL0_1_378            --SSDKV---SHGQLTEVEQNWSNMALELQNLNG----------------
UniRef90_I3K1N0_1_478            --SGEKVGVGSQGQLVAMEQNWNNMVLEVHNLNGKNSASASS--------
UniRef90_H2LFL5_1_470            --NGEKVSIGSHGQLSEMEQNWRNMALELHNPNGNSSSSSSSSYAEPLPS
UniRef90_M7BWJ7_1_486            PMQAITPCFNGSSQVLAVEQNWANLAVEQQRKPPASSSAASSAPSSIGQQ
UniRef90_K7F163_1_492            PVQAITCYFNGSSQALAAEQNWANLAVEQQRKPPAR-SATSSSPSSTRQQ
UniRef90_H0ZM32_1_512            PTPAVINYFNSSSRALTSEQDWVNMAAEHQGKVSSS-SAGSSTPSSVRHP
UniRef90_H3AH16_1_396            ------YFINSNNHRLTTDQNWANLAAEQQTQERKP-ASSSSTNSSVGN-
UniRef90_B0BM18_1_388            ------LMSHHEHLIAASEQNWTNLAVERERMSRSS-SGSSCSATTSALD
UniRef90_Q4S8R0_1_345            -------------NKVVYKQNRDNMAVER-KGKPEDEVVMERKP------
UniRef90_I3KYH7_1_341            -------------SKVAYKQNRDNMAVER-KGKAEENDMKGRKI------
UniRef90_H2M0G7_1_345            -------------SKAVHKQNRDNLAVER-KSRAEGDDSEKTKS------
UniRef90_H3A471_1_360            -------------SKAAHKQNRDNLAVER-NGRSSDSN-HSPKE------
UniRef90_Q6GLA5_1_353            -------------SQAARKQNRDNQAVER-NGIHDLNVASESSS------
UniRef90_UPI0002A4A1D5_1_346     -------------SKAAYKQNKDNLAVERGNSKQEECDLKEKKG------
UniRef90_S4RXA6_1_456            RRRVAQATSGDARHARHAPQPVSRSLAMRTPPPPHGAPPPP---------
UniRef90_P16863_1_360            NR-MGQAGS---TISNTHAQPFDFSDEHQN--TKKMAPGHE---------
UniRef90_H2LU51_1_404            -------------GDPGGKPDCHKDVKEGALSRMEQNSNSNNTSS-----
UniRef90_I3KYL3_1_401            -------------GDPGGKPDSQKEGKEGVPSPTEHNNNSNNTSS-----
UniRef90_G3NC44_1_402            -------------GDPVAKPDLHKDGKEGVPSPTEQNSNSNNTSS-----
UniRef90_H2SSL2_1_392            -------------GDPGAKSDRRKDGKEGIPSPTEQNSNSNNTSS-----
UniRef90_UPI0002C46B7E_1_433     -------------ENYSGNDEKCILDEQETVLSNEMHTINTICSQ-----
UniRef90_G3ULF1_1_432            -------------DSYSGNDEKCILDEQETVLSSKMHTISTTGSH-----
UniRef90_C9IYK1_1_407            -------------DNHSVNDEKCILDEQETVLSNEISTLSTSCSH-----
UniRef90_H0Y0G7_1_436            -------------DNHNMK-KKYSLDEQETELSNEIYTLSTTCSH-----
UniRef90_L9JD22_1_408            -------------DNHSVNNEKCIVDEQETVLSNEMCTLSTSCSP-----
UniRef90_F1N617_1_436            -------------DNHIGNDDKGILDEQETLLS-EMCTFSTACGH-----
UniRef90_F1SV25_1_385            -------------DSHRGNDERGISNEQETVLSNKMCTLSTACGH-----
UniRef90_L5JUH5_1_307            --------------------------------------------------
UniRef90_UPI00020369CA_1_441     -------------NNHTENRGNYTHHNQENKLPKKKPATNALAGQ-----
UniRef90_R4GLX1_1_405            -------------NNHTENRGNYTHHHQENKLPKKRPATNAVAGQ-----
UniRef90_UPI00035A19E6_1_466     -------------NACTESSSNYTHCNQGSKSPKKRPATNALDNQ-----
UniRef90_F6WA84_1_463            -------------ET----GKKSPASDERNKPSDETCLSSIVEGY-----
UniRef90_G1SG18_1_430            -------------DSSFSKKEWAEKDQHSGQLHVHSPCPQDASTR-----
UniRef90_UPI00032B0E64_1_413     -------------DSSFSKKEWAEKDQHSGQLHVHSPCPHEARPR-----
UniRef90_UPI0002C65D9D_1_412     -------------DPCS-KKDWAEKDQHSGQLHVHSPCPWDDDAR-----
UniRef90_F7CF28_1_383            -------------DPCS-KKDWPEKDQHSGQLHVHSPCPWDDDAR-----
UniRef90_Q969M2_1_435            -------------DPSNGKKDWSEKDQHSGQLHVHSPCPWAGSAG-----
UniRef90_L9JVZ3_1_406            -------------DPSRSKKDWSEKDQHSGQLHTHCPSPWDGGAR-----
UniRef90_I3M407_1_414            -------------DSSCSKRDCTEKDQHNGQLHIHSPCPQDGKAR-----
UniRef90_L5JMB7_1_435            -------------DPCCSRKDWTGEDQHSGQLHVHSPCPQHGCAK-----
UniRef90_L8J083_1_417            -------------DPCYSKKDWAEKNQHNGQLHVHSPCPWDDSAR-----
UniRef90_UPI0002BCCFF6_1_421     -------------DPCCSKKDWAKADQHRGQLHVHSPCPWDDGAR-----
UniRef90_F1RY20_1_421            -------------DPCFSKKDWAEKDQHSGQLHVHSPCPWEGGAR-----
UniRef90_UPI0002C59166_1_412     -------------DSCCVKEEWAEKDQHSGQLHARSPCLWEAGTQ-----
UniRef90_G3TBX7_1_436            -------------VPCYDKKDWAQKDQHSGQLLVHSPCTWNSSVR-----
UniRef90_G3GSS7_1_405            -------------DVSCSKRDWAERVTNSGQLHVHSPCPQDGNGR-----
```

```
UniRef90_UPI0003316158_1_413      -------------DACCSQEDSAEKDQHGGQLHVHEPGLWTHSAT-----
UniRef90_G3WUN4_1_443             -------------AFPLGKKNWSRRDQHHRMLQFSSVCPQDLDCC-----
UniRef90_F6W379_1_264             --------------------------------------------------
UniRef90_M7BL54_1_431             -------------EQRRCSTDRPRSEQHHGQVPRLHKHPENPRER-----
UniRef90_UPI0002B44362_1_276      ----------------------------------------GPSEE-----
UniRef90_UPI000298933C_1_429      -------------MPAQGLHQVPPVEQQCAVGTRKPSYSSEESSE-----
UniRef90_H3CDZ2_1_426             -------------VPIQGVQQVAPAEQHRLSGLRKPSCSSEESSE-----
UniRef90_I3IZH7_1_440             -------------AEIQGLHQLGAMERRYTLENRKPSCSSDDSNG-----
UniRef90_E7F3P4_1_389             -------------PEIQVLQQLGIKERRSIPDNRLQSCSSEDS-G-----


UniRef90_UPI0002C691BE_1_358      --------------------------------------------------
UniRef90_F7BKC0_1_358             --------------------------------------------------
UniRef90_Q0VCR2_1_359             --------------------------------------------------
UniRef90_G3TUX5_1_358             --------------------------------------------------
UniRef90_L9JKN2_1_358             --------------------------------------------------
UniRef90_P36382_1_358             --------------------------------------------------
UniRef90_UPI0003315E33_1_350      --------------------------------------------------
UniRef90_UPI000333FB64_1_358      --------------------------------------------------
UniRef90_UPI000334540D_1_357      --------------------------------------------------
UniRef90_L5K3R8_1_358             --------------------------------------------------
UniRef90_G1TQ37_1_358             --------------------------------------------------
UniRef90_UPI0001776F40_1_352      --------------------------------------------------
UniRef90_G3H2Q9_1_358             --------------------------------------------------
UniRef90_Q01231_1_358             --------------------------------------------------
UniRef90_G5BBL6_1_357             --------------------------------------------------
UniRef90_H0WCZ7_1_358             --------------------------------------------------
UniRef90_G3X1A3_1_358             --------------------------------------------------
UniRef90_H0ZTI1_1_370             --------------------------------------------------
UniRef90_P18860_1_369             --------------------------------------------------
UniRef90_G1KPB5_1_370             --------------------------------------------------
UniRef90_K7FWT6_1_369             --------------------------------------------------
UniRef90_F1QL21_1_400             --------------------------------------------------
UniRef90_UPI0002A497DC_1_408      --------------------------------------------------
Input_protein_seq                 ----------------GPPAEEGAEPEVGEKKEEAERL------------
UniRef90_UPI00027FC10C_1_397      ----------------GPPAEEGAEPEVGEK-EEAERA------------
UniRef90_L9JGQ0_1_441             ----------------GPPVEEAAEPEVGEKRLEAERL------------
UniRef90_P28236_1_440             ----------------EPPIEEAVEPEVGEKKQEAEKV------------
UniRef90_G5BBL7_1_440             ----------------QQPVEETTEPEVEEKKPEAEKV------------
UniRef90_M3XED1_1_440             ----------------EQPVEEGAEPEVGDKRQEAERV------------
UniRef90_L5K685_1_440             ----------------EPPAEEGAEAEVAEKRQEADRV------------
UniRef90_F6VG96_1_441             ---------------EEQPVEEAAEPEAGEKSQEAEKV------------
UniRef90_P55917_1_440             ----------------EQPIEAAAEPEVGDKSQEAERV------------
UniRef90_UPI000329268E_1_442      ----------------EQHVEAGAEPELGEKRQEAERV------------
UniRef90_G3UBH4_1_410             ----------------E---------------------------------
UniRef90_I3N7I6_1_431             ----------------EPPAEEGAEPEVGEKPREGERA------------
UniRef90_G3X210_1_429             ---------------EEEEGEVPPEEEEKRQEPEAVTP------------
UniRef90_F7BI06_1_372             --------------------------------------------------
UniRef90_UPI000333FA9E_1_424      -------------------ADQGAESE-----ERAAQV------------
UniRef90_K7EY71_1_406             -----------------EKVEAGEEEQDQEEEEEHQEP------------
UniRef90_M7BCA4_1_394             -----------------EKVEAGEEEQ---------EP------------
UniRef90_P36381_1_400             -----------------EKVKAEEEEE---QEEEQQAP------------
UniRef90_H2MUH4_1_428             ------------------GVTLHEDEVEEEQVQPPEAE------------
UniRef90_UPI00022B4A0D_1_396      ------------------GVTLHEEEPEE---------------------
UniRef90_H3D8V1_1_361             ------------------GVTLPR--------------------------
UniRef90_G3NHJ7_1_420             ------------------GVTLRQEAEEVQPAEAEAER------------
UniRef90_Q503J6_1_391             -----------------SGILRRDEDEDE---------------------
UniRef90_F6QCF2_1_438             -----------------VKVQASDAFEQASSERAASVR------------
UniRef90_UPI00034FAB61_1_453      -----------------VKVQASDAFEQASSERAASVR------------
UniRef90_UPI0001DE8510_1_439      -----------------VKVQASDAFEQASSERAASVR------------
UniRef90_H3A837_1_370             -------------------EEIPDPQAEPTEIAAADTR------------
UniRef90_G1KPB7_1_391             --------------------EKVEQPEQEVAEEVQDEQ------------
UniRef90_B0S6Z4_1_382             --------------------------------------------------
UniRef90_G1PZ72_1_426             ----------------EQPGEEGADPERVAVAERLDAE------------
UniRef90_UPI000155FB82_1_448      -----------------SPQQLPQLGGAGSKAPALLA-------------
UniRef90_F6U7L2_1_428             -----------------SPQQLPQLGGAGSKAPALLA-------------
UniRef90_UPI0002C6341D_1_440      -----------------SPQQLPQGDRAGSKVPVLLA-------------
UniRef90_M3Z8M0_1_445             -----------------STKELPQEGGVGSSAPGLLE-------------
UniRef90_G1Q1M8_1_411             -------------------LQAPQEGREGSKESVSLE-------------
UniRef90_G1PYG2_1_394             -------------------CKPLKR-----AEEAALE-------------
UniRef90_L5L5Q1_1_430             -----------------SSLTPPGSPQPPPQEPVPVE-------------
UniRef90_UPI0002BCF55A_1_410      -----------------LSAPAAPKPPAG-PQQPPPE-------------
UniRef90_P41987_1_407             -----------------APTPPAAESPGGGPQQSLPE-------------
UniRef90_J9JHI9_1_433             -----------------AAAPSASPSPAGSTGQLPPD-------------
UniRef90_Q9Y6H8_1_435             -------------------STPAAPSPVGSSSPPLAHEAEAGAA------
UniRef90_G3QHB2_1_399             -------------------STPAAPSP-----------------------
```

```
UniRef90_F7B1T8_1_432           -------------------PTPAAPSPTGSSSQPPTHEAEAGAA------
UniRef90_L9L2D3_1_460           -------------------SSPAAPSPTGSGTQPALQEGRAGATGAT---
UniRef90_Q64448_1_417           -------------------SSPDGRKGLI---------------------
UniRef90_G3H8J5_1_417           -------------------SSPEGRKGLT---------------------
UniRef90_UPI0003333DA4_1_405    -------------------FSPLGGEDTPG--------------------
UniRef90_I3NC37_1_417           -------------------SRGTAPQLLE---------------------
UniRef90_H0XD37_1_430           -------------------SSPQPLPPEGEGDGE----------------
UniRef90_G1U3S9_1_407           -------------------QPPRPEAGPGGSAPLLLG-------------
UniRef90_UPI00032AEFC1_1_413    -------------------LPPPREAGPGGSAPLLLGG------------
UniRef90_UPI00034F30A8_1_402    -------------------PQPLQEAGPG---------------------
UniRef90_UPI0003340A67_1_370    -------------------PQPVQEAGLR---------------------
UniRef90_UPI0002234D0E_1_449    -----------------STPQLPLEVRAGSQAPLLLV-------------
UniRef90_G3TTN5_1_429           ---------------------------AGSQAPLLLV-------------
UniRef90_UPI0002C457ED_1_447    -----------------STPQLPLEGMAGGQTPLLLV-------------
UniRef90_UPI000333D50E_1_427    -----------------SSPQPSLESRARSGVPLLLV-------------
UniRef90_UPI0003343FBC_1_423    -------------------SIPAGLTTPGNTQQAPRE-------------
UniRef90_F7G5V2_1_446           -----------------SVRQLLQERGDSGGGSKVPLL------------
UniRef90_G3VY19_1_428           ------------------------------GGSKVPLL------------
UniRef90_UPI000155BBAC_1_459    -----------------STQQNPPEVGGGGEGSEAPLL------------
UniRef90_F7ETY8_1_425           -----------------STQQNPPEV------------------------
UniRef90_I3KXW1_1_474           --------------EQHQSVNGALLLPTSNTTSNTNITNATANAASSGSS
UniRef90_UPI00022B3AE8_1_449    --------------EQHQSVNGALLLPTSNTTS-----------------
UniRef90_UPI0002A48057_1_447    --------------NKYQQHDAATSGGRPGSPSN----------------
UniRef90_H2MCD5_1_421           --------------NKYQQQ------------------------------
UniRef90_H2UTF3_1_420           -------------------------SSSPGSASN----------------
UniRef90_UPI000298F1F6_1_460    PVDAELLPSASSNITNTTTTVTASGSSSPGSASN----------------
UniRef90_H3DE08_1_376           --------------------------------------------------
UniRef90_Q8JFD2_1_434           ---------------VRQSKDAAQLASTPSSAG-----------------
UniRef90_H2T4Y1_1_438           -------------------------PTLPSPP------------------
UniRef90_H2T4Y2_1_428           -------------------------PTLPSPP------------------
UniRef90_H3BZL0_1_378           -------------------------ETMEEP-------------------
UniRef90_I3K1N0_1_478           --------SPQVEKTPTPEEQQSAFPTLPRNTPLSSLMPKETAMKEE---
UniRef90_H2LFL5_1_470           SPTSASSFSPEDEAALQQENQLSMFPTLPRNTP-----------------
UniRef90_M7BWJ7_1_486           ----------------SPKQEAASEQPLPSPPLLPP------------VA
UniRef90_K7F163_1_492           ----------------SPKQEEASELPLPLPPLLPP------------VA
UniRef90_H0ZM32_1_512           ----------------LPEQEEPLEQLLPPPAVLP-------------IA
UniRef90_H3AH16_1_396           --------------------EGAQEAPTP---------------------
UniRef90_B0BM18_1_388           ----------------SLRNDGGCEEMLT---------------------
UniRef90_Q4S8R0_1_345           --------------------------------------------------
UniRef90_I3KYH7_1_341           --------------------------------------------------
UniRef90_H2M0G7_1_345           --------------------------------------------------
UniRef90_H3A471_1_360           --------------------------------------------------
UniRef90_Q6GLA5_1_353           --------------------------------------------------
UniRef90_UPI0002A4A1D5_1_346    --------------------------------------------------
UniRef90_S4RXA6_1_456           ------------------ASPLLLLQPWAASSPAACPS------------
UniRef90_P16863_1_360           ------------------MQPLTILDQRP---------------------
UniRef90_H2LU51_1_404           --------------------------------------------------
UniRef90_I3KYL3_1_401           --------------------------------------------------
UniRef90_G3NC44_1_402           --------------------------------------------------
UniRef90_H2SSL2_1_392           --------------------------------------------------
UniRef90_UPI0002C46B7E_1_433    --------------------------------------------------
UniRef90_G3ULF1_1_432           --------------------------------------------------
UniRef90_C9IYK1_1_407           --------------------------------------------------
UniRef90_H0Y0G7_1_436           --------------------------------------------------
UniRef90_L9JD22_1_408           --------------------------------------------------
UniRef90_F1N617_1_436           --------------------------------------------------
UniRef90_F1SV25_1_385           --------------------------------------------------
UniRef90_L5JUH5_1_307           --------------------------------------------------
UniRef90_UPI00020369CA_1_441    --------------------------------------------------
UniRef90_R4GLX1_1_405           --------------------------------------------------
UniRef90_UPI00035A19E6_1_466    --------------------------------------------------
UniRef90_F6WA84_1_463           --------------------------------------------------
UniRef90_G1SG18_1_430           --------------------------------------------------
UniRef90_UPI00032B0E64_1_413    --------------------------------------------------
UniRef90_UPI0002C65D9D_1_412    --------------------------------------------------
UniRef90_F7CF28_1_383           --------------------------------------------------
UniRef90_Q969M2_1_435           --------------------------------------------------
UniRef90_L9JVZ3_1_406           --------------------------------------------------
UniRef90_I3M407_1_414           --------------------------------------------------
UniRef90_L5JMB7_1_435           --------------------------------------------------
UniRef90_L8J083_1_417           --------------------------------------------------
UniRef90_UPI0002BCCFF6_1_421    --------------------------------------------------
UniRef90_F1RY20_1_421           --------------------------------------------------
UniRef90_UPI0002C59166_1_412    --------------------------------------------------
UniRef90_G3TBX7_1_436           --------------------------------------------------
UniRef90_G3GSS7_1_405           --------------------------------------------------
```

```
UniRef90_UPI0003316158_1_413       --------------------------------------------------
UniRef90_G3WUN4_1_443              --------------------------------------------------
UniRef90_F6W379_1_264              --------------------------------------------------
UniRef90_M7BL54_1_431              --------------------------------------------------
UniRef90_UPI0002B44362_1_276       --------------------------------------------------
UniRef90_UPI000298933C_1_429       --------------------------------------------------
UniRef90_H3CDZ2_1_426              --------------------------------------------------
UniRef90_I3IZH7_1_440              --------------------------------------------------
UniRef90_E7F3P4_1_389              --------------------------------------------------


UniRef90_UPI0002C691BE_1_358       ------------------------------------KPEEL--NEVSPGH
UniRef90_F7BKC0_1_358              ------------------------------------KPEVP--NGVSPGH
UniRef90_Q0VCR2_1_359              ------------------------------------KPEVP--NEGSPGP
UniRef90_G3TUX5_1_358              ------------------------------------RPEVP--NGVSPGH
UniRef90_L9JKN2_1_358              ------------------------------------KPEVP--NGASPGP
UniRef90_P36382_1_358              ------------------------------------KPEVP--NGVSPGH
UniRef90_UPI0003315E33_1_350       ------------------------------------KPEVP--NEVSPGH
UniRef90_UPI000333FB64_1_358       ------------------------------------RPEVP--HEVPPGH
UniRef90_UPI000334540D_1_357       ------------------------------------KPEVP--NGGAPDH
UniRef90_L5K3R8_1_358              ------------------------------------KPEVP--SEVSSGH
UniRef90_G1TQ37_1_358              ------------------------------------KPDVP--DGVSPGH
UniRef90_UPI0001776F40_1_352       ------------------------------------KPEVP--TEVSPGH
UniRef90_G3H2Q9_1_358              ------------------------------------KPEEH--NGASPGH
UniRef90_Q01231_1_358              ------------------------------------KPEYA--SGASAGH
UniRef90_G5BBL6_1_357              ------------------------------------KPDVP--HGVSSGH
UniRef90_H0WCZ7_1_358              ------------------------------------KPDVP--SGVSSGH
UniRef90_G3X1A3_1_358              ------------------------------------GPEMA--NGVPNAH
UniRef90_H0ZTI1_1_370              ------------------------------------SPEVA--SECAAP-
UniRef90_P18860_1_369              ------------------------------------SPEVA--SECAAP-
UniRef90_G1KPB5_1_370              ------------------------------------NPEVR--NNSAPG-
UniRef90_K7FWT6_1_369              ------------------------------------SPEAP--NDSAPH-
UniRef90_F1QL21_1_400              ------------------------------------SEMTP--STPSTPS
UniRef90_UPI0002A497DC_1_408       ------------------------------------SPESRDNNCLQIQN
Input_protein_seq                  ----------TTEEQEKVAVPEGEKVETPGVDKEGEKEEPQSEKVSKQGL
UniRef90_UPI00027FC10C_1_397       ----------TTEGQEKVAVPEGEKAETPGVEKDGEKEELQS-EVAKQGL
UniRef90_L9JGQ0_1_441              ----------ATEGQEVVPVAEGEKVETAGVGQESEKDELLAEKASKQGL
UniRef90_P28236_1_440              ----------APEGQETVAVPDRERVETPGVGKEDEKEELQAEKVTKQGL
UniRef90_G5BBL7_1_440              ----------TLEVQEAITEPEGERVETPGVGKEGEKEELQIEKVTKQGL
UniRef90_M3XED1_1_440              ----------STEGQETLAVLEGEKVETPEVGKEGEKDELQAEKVAKQGL
UniRef90_L5K685_1_440              ----------STEGQETNDELEGDRVETPGAGKEGEKEELPAEKASKQGL
UniRef90_F6VG96_1_441              ----------STEGQETTAVLEGEKVETPKVGKEAEKEELQAEKVSKQEL
UniRef90_P55917_1_440              ----------STEGEETLAVLEEEKVEPPEVEKEAEKEETPPEKVSKQEL
UniRef90_UPI000329268E_1_442       ----------STEGQEMVVVPEGEKIEPPEGEKQGKEEELQAEMVSKQRV
UniRef90_G3UBH4_1_410              ------------------AVLEGEKGETPKVGKESEKEELQAEQISKQGL
UniRef90_I3N7I6_1_431              ----------TLEGQDAVAVPDGDKVETPAVGKEGDKEELQAEKMTKPGL
UniRef90_G3X210_1_429              ----------EMQEATVEQEEEKEKVGTP-ERKAGE----------KQEL
UniRef90_F7BI06_1_372              ------------------QEEEKEKAGTPVEMKAGD----------KQEL
UniRef90_UPI000333FA9E_1_424       ----------TTEGQE--AVLEGENAESFPASAQAEKEELPAEKASKPGP
UniRef90_K7EY71_1_406              ----------EEELE-----------VKKMAAEQELPGEELPAVP-----
UniRef90_M7BCA4_1_394              ----------EEEQE-----------VEKVVEEQEPPGEELPSVP-----
UniRef90_P36381_1_400              ----------QEEPG-----------VKKAEEEVVSDEVEGPSAP-----
UniRef90_H2MUH4_1_428              ----------AEKVEEMADEDEEEEVEEAENGEDAAPAEAGLEAT-----
UniRef90_UPI00022B4A0D_1_396       ---------------------------------GATPAEAGTEAA-----
UniRef90_H3D8V1_1_361              ----------------------------------------AAEAP-----
UniRef90_G3NHJ7_1_420              ----------VEKG-----ADEDLEVEEAGNGEGVNPEETRMEVT-----
UniRef90_Q503J6_1_391              -----------------------------------LAVEADMEAS-----
UniRef90_F6QCF2_1_438              ----------SPSVRAPSDRAPSERALSERAPTRAASEHAGSEHTPSVRT
UniRef90_UPI00034FAB61_1_453       ----------SPSVRAPSDRAPSERALSERAPTRAASEHAGSEHTPSVRT
UniRef90_UPI0001DE8510_1_439       ----------SPSVG--LDQSPNE-----PTPLRALPEQGNPNHTFPDPE
UniRef90_H3A837_1_370              --------------------------------------------------
UniRef90_G1KPB7_1_391              ----------GAEG-----------------FKEKAGVKVGEEKH-----
UniRef90_B0S6Z4_1_382              ---------------------------------ELPTEEPPREGD-----
UniRef90_G1PZ72_1_426              ----------RVSTEGRETPAVEGEGEASQVGKEGEEGELPAEKVAKQRL
UniRef90_UPI000155FB82_1_448       ----------NGNG------------SPLGESKLEVTPDE-EEWAVTTAV
UniRef90_F6U7L2_1_428              ----------NGNG------------SPLGESKLEVTPDE-EEWAVTTAV
UniRef90_UPI0002C6341D_1_440       ----------NGNG------------SPWGEKKLEVTPDE-EERAVATVV
UniRef90_M3Z8M0_1_445              ----------NGNG------------SSLGESKLEVTPDDGEEQPVTTAV
UniRef90_G1Q1M8_1_411              ----------NGDS------------SLLGESRLEVTPDE-QQQAVT-AV
UniRef90_G1PYG2_1_394              ----------NGDS------------SLLGESRLEVTPDE-QQQAVT-AV
UniRef90_L5L5Q1_1_430              ----------NGNG------------HSLGESSLAVTPDQ-GVPAVTSPV
UniRef90_UPI0002BCF55A_1_410       ----------GGAG-------------SSGDS-------D-GEGAVT-AV
UniRef90_P41987_1_407              ----------GAAG-------------SSGDS-------D-GEGAVT-AV
UniRef90_J9JHI9_1_433              ----------DGTGGGEPGLLLTGSRSSLGDSKLAVTPDD-AEQPVTTPA
UniRef90_Q9Y6H8_1_435              ------PLLLDGSG------------SSLEGSALAGTPEEEE-QAVTTAA
UniRef90_G3QHB2_1_399              --------LLDGSG------------SSLEGSALAGTPEEEE-QAVTTAA
```

```
UniRef90_F7B1T8_1_432               ------HLLLDGSG------------SSLEGSALAGTPEEEE-QAVTTAA
UniRef90_L9L2D3_1_460               ----APLLLVDGSG------------SSLEDSALAVTPEEEE-QAVTTAA
UniRef90_Q64448_1_417               ----------DSSG------------SSLQESALVVTPEEGE-QALATTV
UniRef90_G3H8J5_1_417               ----------DSSG------------SSLEESALVVTPEEGE-QALATTV
UniRef90_UPI0003333DA4_1_405        ----------DGSG------------SSLEESALAVTPEEEEEQALATTV
UniRef90_I3NC37_1_417               ----------DRSG------------SSLEESALAVTPEEEE-QALTTTA
UniRef90_H0XD37_1_430               ---------VEGSG------------SSLEDSALAVTPEEEEEQAVTTAA
UniRef90_G1U3S9_1_407               ----------DASG------------SGSEEVPLGATPEEEE-PAVTTAA
UniRef90_UPI00032AEFC1_1_413        ----------DASR------------SSSEEGPLGATPGEEE-PAVTAAA
UniRef90_UPI00034F30A8_1_402        ----------DGSG------------SSLEGRVRVGTPEAE--------A
UniRef90_UPI0003340A67_1_370        ----------DGSG------------SSLAGSARGGTPG-----------
UniRef90_UPI0002234D0E_1_449        ----------NGST------------SSLGENKLEVTPEE-KVQAETTRV
UniRef90_G3TTN5_1_429               ----------NGST------------SSLGENKLEVTPEE-KVQAETTRV
UniRef90_UPI0002C457ED_1_447        ----------NGST------------SNLEESKLEVTPEE-EVQAGTTRV
UniRef90_UPI000333D50E_1_427        ----------NGST------------SSLRGSKLEVCPEE-EVQAGATSV
UniRef90_UPI0003343FBC_1_423        ----------GRAG--------NRVLLVNGNGRSSADTPDAGEPAAITNI
UniRef90_F7G5V2_1_446               --------VINGSSS------------SLGATKSEVTSEG-EKQPGTTTV
UniRef90_G3VY19_1_428               --------LINGSSS------------SLGAAKSEVNSEG-EKQPGTTTV
UniRef90_UPI000155BBAC_1_459        --------LVNGGS--------------LGGGSGGEAEAG-EEWPGTTRV
UniRef90_F7ETY8_1_425               ----------NGGS--------------LGGGSGGEAEAG-EEWPGTTRV
UniRef90_I3KXW1_1_474               PGNASNAGSWDGGKS----------------EQDEGHV-------TTT-V
UniRef90_UPI00022B3AE8_1_449        --------SWDGGKS----------------EQDEGHV-------TTT-V
UniRef90_UPI0002A48057_1_447        ------ADIWGSGRS----------------MQEKGHI-------TTTTV
UniRef90_H2MCD5_1_421               ------SNIWGSGRS----------------MQEKGHI-------TTTTV
UniRef90_H2UTF3_1_420               ------TGSWGGGRK----------------ERGENGV-------STTRV
UniRef90_UPI000298F1F6_1_460        ------TGSWGGGRK----------------ERGENGV-------STTRV
UniRef90_H3DE08_1_376               ---------WGGGTN----------------EQEGRRV-------STTRV
UniRef90_Q8JFD2_1_434               ----------GGLST----------------GPEEGHV-------TTT-V
UniRef90_H2T4Y1_1_438               -------NSSSSSLQ----------------VPHDDVT-------VVTKA
UniRef90_H2T4Y2_1_428               -------NSSSSSLQ----------------EETNPPL-------PQGEQ
UniRef90_H3BZL0_1_378               ---------STASCD----------------VPPDDVT-------VVTKA
UniRef90_I3K1N0_1_478               ------TTVITAPSK----------------VLHNDFT-------VVTRA
UniRef90_H2LFL5_1_470               ------LSIIAPEVR----------------AADEESP-------AVQNE
UniRef90_M7BWJ7_1_486               AVNSSSSTSLSRGSSS-----------KWDVEGEEEATKEWPVSAACTTV
UniRef90_K7F163_1_492               AVSSSSSTSLSRGSSS-----------KWDAEGEEEATKAWPLSVACTTV
UniRef90_H0ZM32_1_512               SANSGSSTSLSGASGS-----------KWDVEGEAELSGARPVSAACTTV
UniRef90_H3AH16_1_396               ----GSSGSLGGGK-----------------QGQEDGN--------VTTV
UniRef90_B0BM18_1_388               -------TEASGSS-----------------ARAEDRP-------VTTRV
UniRef90_Q4S8R0_1_345               ---------------------------------------------TCPAF
UniRef90_I3KYH7_1_341               ---------------------------------------------SSPVL
UniRef90_H2M0G7_1_345               ---------------------------------------------SSPAS
UniRef90_H3A471_1_360               ---------------------------------------------GKAAP
UniRef90_Q6GLA5_1_353               ---------------------------------------------ELDQF
UniRef90_UPI0002A4A1D5_1_346        ---------------------------------------------AGSAP
UniRef90_S4RXA6_1_456               --------------------------SSAAVAAALAAALEDEAEVLTATV
UniRef90_P16863_1_360               --------------------------------------------------
UniRef90_H2LU51_1_404               --------------------------ETRSPAADKQGEAEEARSPHHDDM
UniRef90_I3KYL3_1_401               --------------------------ETRSPPSDKQEEREDQSPPHHEDL
UniRef90_G3NC44_1_402               --------------------------ETRSPPSDKQEEPEEQSSPPLERM
UniRef90_H2SSL2_1_392               --------------------------DTHSLPVDKHEEPEEP--------
UniRef90_UPI0002C46B7E_1_433        --------------------------IQHISSSNNEGTHKIFGKEVNDNQ
UniRef90_G3ULF1_1_432               --------------------------LQHINS-NNDGTHKISGKDVDDNQ
UniRef90_C9IYK1_1_407               --------------------------FQHISSNNNKDTHKIFGKELNGNQ
UniRef90_H0Y0G7_1_436               --------------------------LQPIGSNNNKDTHKIFGKEVNDSQ
UniRef90_L9JD22_1_408               --------------------------LGHIGSNNNKDNHKIFEKEVNDKQ
UniRef90_F1N617_1_436               --------------------------LQNISSSNKEDTHKLSGKEVNGNQ
UniRef90_F1SV25_1_385               --------------------------LQNVSSSNKEDTHKISGKEVNGNQ
UniRef90_L5JUH5_1_307               --------------------------------------------------
UniRef90_UPI00020369CA_1_441        --------------------------TQNASTNCTEGLLARPGTEMRNSQ
UniRef90_R4GLX1_1_405               --------------------------TQNAGINCSEGLLGRPGTERRSSQ
UniRef90_UPI00035A19E6_1_466        --------------------------TQNASTNNNEGFLGQLWTEMHDAQ
UniRef90_F6WA84_1_463               --------------------------PANANLSHSEGIISVVRAEIGGTH
UniRef90_G1SG18_1_430               --------------------------IQHSGQQPDH-SSFAVQNTTIQSW
UniRef90_UPI00032B0E64_1_413        --------------------------IQHLGQQPNH-SSFALQNTASQSC
UniRef90_UPI0002C65D9D_1_412        --------------------------IQHLGQQPDH-SSFGLQNTLSQSC
UniRef90_F7CF28_1_383               --------------------------IQHLGQQPDH-SSFALQKTTSQSC
UniRef90_Q969M2_1_435               --------------------------NQHLGQQSDH-SSFGLQNTMSQSW
UniRef90_L9JVZ3_1_406               --------------------------IQHRGQQQTH-SSFGPHNTVPQSW
UniRef90_I3M407_1_414               --------------------------IQHPGQQPCH-SSFGLRNTVSQSW
UniRef90_L5JMB7_1_435               --------------------------IQHSGQHPDL-SSFGLQNTMSQSW
UniRef90_L8J083_1_417               --------------------------IQHPGQQPDH-SLFGLQNIRPHSW
UniRef90_UPI0002BCCFF6_1_421        --------------------------IQHSAQQPDR-SPFGLQTTTPQSW
UniRef90_F1RY20_1_421               --------------------------IQHSGQQPDH-SSLGLQNPTSQSW
UniRef90_UPI0002C59166_1_412        --------------------------SQHPGQQPDL-SS-GLHNAKSHCC
UniRef90_G3TBX7_1_436               --------------------------MQDHGQQPDHPSSFGFQNTVSQSW
UniRef90_G3GSS7_1_405               --------------------------IQHPGQQPCH-SLFGSKNVMSQSW
```

```
UniRef90_UPI0003316158_1_413      --------------------------LQHPGQHSGK--PFGPQNAKCHSW
UniRef90_G3WUN4_1_443             --------------------------VQYFQQCKHQYQGQHPQSSFSRGG
UniRef90_F6W379_1_264             --------------------------------------------------
UniRef90_M7BL54_1_431             --------------------------EQHYRQLSNQHLGQPSRHPSSSSE
UniRef90_UPI0002B44362_1_276      --------------------------D-------------PSAYNSKKS-
UniRef90_UPI000298933C_1_429      --------------------------PH--VRPQYAG-PRATLVASHMEI
UniRef90_H3CDZ2_1_426             --------------------------PH--VKPQYAG-PRATLVASHMEI
UniRef90_I3IZH7_1_440             --------------------------PHGSGKPHYAG-PRPTLTGGHMEI
UniRef90_E7F3P4_1_389             --------------------------PKGSEPPKYSQQPRASFRASHIEI


UniRef90_UPI0002C691BE_1_358      RLPRG-----------------YQSDKRCLSK------ASSKARSDDLSV
UniRef90_F7BKC0_1_358             RLPHC-----------------YQNDKPRLSK------ASSKARSDDLSV
UniRef90_Q0VCR2_1_359             SLPHG-----------------YQSDKRRLSK------ASSKARSDDLSV
UniRef90_G3TUX5_1_358             RLPHG-----------------YHSDKRRLSK------ASSKARSDDLSV
UniRef90_L9JKN2_1_358             RLPHG-----------------YHSDKRRLSK------ASSRARSDDLSV
UniRef90_P36382_1_358             RLPHG-----------------YHSDKRRLSK------ASSKARSDDLSV
UniRef90_UPI0003315E33_1_350      RLPQG-----------------YPSDKRRLSK------ASSKARSDDLSV
UniRef90_UPI000333FB64_1_358      RLPQA-----------------YHGDKRRPSK------ASSKARSDDLSV
UniRef90_UPI000334540D_1_357      RPPQG-----------------FGGDKRRLSK------ASSKARSDDLSV
UniRef90_L5K3R8_1_358             RLPHS-----------------YQSDKRRLSK------ASSKARSDDLSV
UniRef90_G1TQ37_1_358             PNPHG-----------------YQSDKRRLSK------ASSKARSDDLSV
UniRef90_UPI0001776F40_1_352      HPTHG-----------------YHSDKRRLSK------ASSKARSDDLSV
UniRef90_G3H2Q9_1_358             RLPPG-----------------YHGDKRRLSK------ASSKARSDDLSV
UniRef90_Q01231_1_358             RLPQG-----------------YHSDKRRLSK------ASSKARSDDLSV
UniRef90_G5BBL6_1_357             CLPHG-----------------CHSEKRRLSK------TSSKARSDDLSV
UniRef90_H0WCZ7_1_358             RLPHG-----------------YYSEKRRLSK------TSSRARSDDLSV
UniRef90_G3X1A3_1_358             RLPHS-----------------YHTDKRRLSK------ASSKARSDDLSV
UniRef90_H0ZTI1_1_370             AFPEN-----------------YFNEKRRLSKAS---RASSKARSDDLSV
UniRef90_P18860_1_369             ALPES-----------------YFNEKRRFSKAS---RASSKARSDDLSV
UniRef90_G1KPB5_1_370             LLSNG-----------------YLPDKRRLSKTS---HASSKARSDDLSV
UniRef90_K7FWT6_1_369             QVSNG-----------------YFNEKRRLSKTS---HASSKARSDDLSV
UniRef90_F1QL21_1_400             SHPG------------------FFRDKRRLSKTSG--TSSNRLRPSDLAV
UniRef90_UPI0002A497DC_1_408      CSPDGL----------------LVKDKRRFSKASG--TSS-RTRADDLSV
Input_protein_seq                 PAEKTPSLCPEL----------TTDDARPLSRLS---KASSRARSDDLTV
UniRef90_UPI00027FC10C_1_397      PAEKTPSLCPEL----------TTDDARPLSRLS---KASSRARSDDLTV
UniRef90_L9JGQ0_1_441             PAEKAPSLCPEL----------TADDARPLSRLS---KASSRARSDDLTI
UniRef90_P28236_1_440             SAEKAPSLCPEL----------TTDDNRPLSRLS---KASSRARSDDLTI
UniRef90_G5BBL7_1_440             SAEKTPSLCPEL----------TADDTRPLSRLS---KASSRARSDDLTI
UniRef90_M3XED1_1_440             PVEKSPSLCPDP----------SRDDTRPLSRLS---KTSSRARSDDLTV
UniRef90_L5K685_1_440             PAEKAPSLCPEL----------TGDDTRPLSRLS---KASSRARSDDLTV
UniRef90_F6VG96_1_441             PAEKAPSLCPEL----------SGDDTRPLSRLS---KASSRARSDDLTI
UniRef90_P55917_1_440             TPEKAPSLCAEL----------PGEDTRPLSRLS---KASSRARSDDLTV
UniRef90_UPI000329268E_1_442      PAEKAPALCAES----------TSDDTRPLSRVS---KTSSRARSDDLTV
UniRef90_G3UBH4_1_410             PAEKAPSLCAEV----------TTDETRPLSRLS---KASSRARSDDLTV
UniRef90_I3N7I6_1_431             SAEKAPSLCPEL----------TAEEARPLSRLS---KASSRARSDDLTI
UniRef90_G3X210_1_429             PVEKAP-LCPEL----------AADDSRPLSRLS---KASSRARSDDLTV
UniRef90_F7BI06_1_372             PVEKIP-LCPEL----------AADDTRPLSRLS---KASSRARSDDLTV
UniRef90_UPI000333FA9E_1_424      ------LLCAEL----------AADDPRALSRLS---KASSRARSDDLTV
UniRef90_K7EY71_1_406             --------------------AET--DTRPPSRLS---KTSSRARSDDLTV
UniRef90_M7BCA4_1_394             --------------------VELTADTRPPSRLS---KASSRART-----
UniRef90_P36381_1_400             --------------------AELATDVRSLSRLS---KASSRARSDDLTV
UniRef90_H2MUH4_1_428             ---------------------DTIDDSRPLSRLS---KASSRARSDDLTI
UniRef90_UPI00022B4A0D_1_396      ---------------------ETIEDNRPLSRLS---KASSRARSDDLTV
UniRef90_H3D8V1_1_361             ---------------------DTIEDTRPLSRLS---KASSRARSD----
UniRef90_G3NHJ7_1_420             ---------------------DTIEDTRPLSRLS---KASSRARSDDLNV
UniRef90_Q503J6_1_391             ---------------------ETIEDTRPLSSLS---KASSRARSDDLTV
UniRef90_F6QCF2_1_438             PSNSATPERVPSRAAS-----EHAPYSRSLSRLS---KASSRARSDDLTV
UniRef90_UPI00034FAB61_1_453      PSNSATPERVPSRAAS-----EHAPLDCAPSERAPSRAASEHAPSDRAPS
UniRef90_UPI0001DE8510_1_439      ALEPDIVEYEPLGPEMPLLNLEFIPDSRSLSRLS---KASSRARSDDLTV
UniRef90_H3A837_1_370             ----------------------------PLSRLS---KASSRARS-----
UniRef90_G1KPB7_1_391             ----------------------MEDEQEPLGGNV---ALPSTADTRP---
UniRef90_B0S6Z4_1_382             ----------------------EVDSLKTPTVLPEVLEEHSEGESVEE--
UniRef90_G1PZ72_1_426             PAEKAPSPGAEL----------AGDDTRPLSRLS---KASSRARSDDLTV
UniRef90_UPI000155FB82_1_448      EMHAPPLPPTD------------PGRSSKASKSSG-----SRARPDDLAI
UniRef90_F6U7L2_1_428             EMHAPPLPPTD------------PGRSSKASKSSG-----SRARPDDLAI
UniRef90_UPI0002C6341D_1_440      AMHAPPLPPTD------------PGRSSKASKSSA-----GHARPDDLAV
UniRef90_M3Z8M0_1_445             EMHVPPLLPAD------------PGRSSKASKSSG-----GRARPNDLAI
UniRef90_G1Q1M8_1_411             EMHMPPLLPTD------------PRRSSKASKASN-----SQARPNDLAI
UniRef90_G1PYG2_1_394             EMHMPPLLPTD------------PRRSSKASKASN-----SQARPNDLAI
UniRef90_L5L5Q1_1_430             DMHAPPLLPAE------------PRRSSTASKASG-----GRARPSDLAI
UniRef90_UPI0002BCF55A_1_410      ELHAPPELPAD------------PGRSSKASKSSG-----GRARASDLAI
UniRef90_P41987_1_407             ELHAPPEPPAD------------PGRSSKASKSSG-----GRARAGDLAI
UniRef90_J9JHI9_1_433             ETHAPPLPPED------------AGRLSKASRASG-----GRARPNDLPI
UniRef90_Q9Y6H8_1_435             QMHQPPLPLGD------------PGRASKASRASS-----GRARPEDLAI
UniRef90_G3QHB2_1_399             QMHQPPLLLGD------------PGRASKASRASS-----GRARPEDLAI
```

```
UniRef90_F7B1T8_1_432          QMHQPPLLLGD------------PGRASKASRASG-----GRARPEDLAI
UniRef90_L9L2D3_1_460          ELHTPPLLLVE------------PGRASKASKASS-----GRARPGDLTI
UniRef90_Q64448_1_417          EMHSPPLVLLD------------PGRSSKS---SN-----GRARPGDLAI
UniRef90_G3H8J5_1_417          EMHSPPLVLLD------------PGRSSKA---SS-----GRARPGDLAI
UniRef90_UPI0003333DA4_1_405   EMHNPPLLLLD------------PGRSSKA---SN-----RRAKPGDLAI
UniRef90_I3NC37_1_417          EMHEPPLLLTD------------PGRSSKA---SS-----GRARPGDLAI
UniRef90_H0XD37_1_430          EMHVQPSEPADPADPADP---VDPGRSSKA---SS-----GRARPDDLAI
UniRef90_G1U3S9_1_407          EMHAPPLLEAD------------AGRCSKAS--G------GRARPSDLAI
UniRef90_UPI00032AEFC1_1_413   EMHAPPSLSAD------------AGRCSKAS--SS-----GRARPSDLAI
UniRef90_UPI00034F30A8_1_402   QVEAPAPLLGD------------TGRSSKAS--S------GRAKPRDLAI
UniRef90_UPI0003340A67_1_370   -VEAPALLPED------------PRRSSKAS--S------GRAKPGDLAI
UniRef90_UPI0002234D0E_1_449   DMHVPPLLLGD------------VGRASKASKSSS-----GWARADDLAI
UniRef90_G3TTN5_1_429          DMHVPPLLLGD------------VGRASKASKSSS-----GWARADDLAI
UniRef90_UPI0002C457ED_1_447   DTHMPPLLLGD------------VGQASKASKLSS-----GRPRADDLAI
UniRef90_UPI000333D50E_1_427   DMHRPPLLLGD------------PGRASKTSRASSS----GRLRADDLAI
UniRef90_UPI0003343FBC_1_423   ELHRPPLLLRD------------AGQSGKAS--SN------RARPNDLAI
UniRef90_F7G5V2_1_446          EMHAPPLLLVD------------SRRLSKASKASS-----GRARSDDLAI
UniRef90_G3VY19_1_428          EMHAPPLLLID------------TRRLSKASKSSS-----GRARSDDLAV
UniRef90_UPI000155BBAC_1_459   EIHESPLLLLD------------LRRLSKASKASS-----CRARSDDLAI
UniRef90_F7ETY8_1_425          EIHESPLLLLD------------LRRLSKASKASS-----CRARSDDLAI
UniRef90_I3KXW1_1_474          EMHEPPVMVSTD-----------PRRLSRASKSSS-----IRARPSDLAV
UniRef90_UPI00022B3AE8_1_449   EMHEPPVMVSTD-----------PRRLSRASKSSS-----IRARPSDLAV
UniRef90_UPI0002A48057_1_447   EMHEPPVTVRTD-----------PRRLSRTSKSSS-----IRTRPSDLAV
UniRef90_H2MCD5_1_421          EMHEPPVTVRTD-----------PRRLSRTSKSSS-----IRTRPSDLAV
UniRef90_H2UTF3_1_420          EMHEPPATAGVD-----------PRRLSRASKSSS-----VRARPSDLAV
UniRef90_UPI000298F1F6_1_460   EMHEPPATAGVD-----------PRRLSRASKSSS-----VRARPSDLAV
UniRef90_H3DE08_1_376          EMHDPPPAPGVD-----------PRRLSRASKSSS-----VRARPSDLAV
UniRef90_Q8JFD2_1_434          EMHEPP-VIFTD-----------ARRLSRASKASS-----VRARPNDLAV
UniRef90_H2T4Y1_1_438          EMHWPPASATT-----------DIRKPSRASKCS------VRARPDDLAV
UniRef90_H2T4Y2_1_428          HSMFPTLPRHTPL--------YALTPPSRASKCS------VRARPDDLAV
UniRef90_H3BZL0_1_378          EMHWPPASAAT-----------DIRKPSRASRSS------VRARPDDLAV
UniRef90_I3K1N0_1_478          EMHQPPAAAGT-----------DNRKPSRASKSGG-----GRARPDDLAV
UniRef90_H2LFL5_1_470          DATAEMHPPPA-----------DTRRPSRACKYSG-----ARARPDDLEV
UniRef90_M7BWJ7_1_486          EMHEPPLLID----------------------------------------
UniRef90_K7F163_1_492          EMHEPPQLID----------------------------------------
UniRef90_H0ZM32_1_512          EMHEPPLLVDTR-------------RLSRASKSSS-----CRARSDDLAV
UniRef90_H3AH16_1_396          EMHEPPVPTDTR-------------RL-----------------------
UniRef90_B0BM18_1_388          EMHQPP--------------------------------------------
UniRef90_Q4S8R0_1_345          EGSADSQRRNS--------------QSSKHS-------------------
UniRef90_I3KYH7_1_341          EMSIENQRRNS--------------QSSKHS-------------------
UniRef90_H2M0G7_1_345          EAPAESQRRNS--------------QSSKHS-------------------
UniRef90_H3A471_1_360          SSPSDNQRSSS--------------RSTKHGNKAKP----ED--------
UniRef90_Q6GLA5_1_353          EKQRLPSRNSR--------------HSSNRSRPG----------------
UniRef90_UPI0002A4A1D5_1_346   GSP--TQPRPG--------------YSTKRS-------------------
UniRef90_S4RXA6_1_456          QLHDPPLHSGGEH----------DIDLRRLSRASS------RVRSDDLQV
UniRef90_P16863_1_360          --------------------------------------------------
UniRef90_H2LU51_1_404          ECGSSEYPTLPAVDTSSCPALSGIVRKSRRVSP-----------------
UniRef90_I3KYL3_1_401          ERARSEYPTLPVSDASSSTVLSGITRKTRR--------------------
UniRef90_G3NC44_1_402          GCTSSEYPTLPV--ASSCATMSGAARKSRRVSP-----------------
UniRef90_H2SSL2_1_392          -LVTSEYPTLPVADATSCPTLSGITRKSRR--------------------
UniRef90_UPI0002C46B7E_1_433   VRERRENDGKDSERNHYSRG------VTVDLENHMRQSPPKVFSLPANCN
UniRef90_G3ULF1_1_432          VKERRENFGTDSERCHYSKG------VSVDLENHVSQSPQKVFSLPANCN
UniRef90_C9IYK1_1_407          LMEKRETEGKDSKRNYYSRGHRSIPGVAIDGENNMRQSPQT---------
UniRef90_H0Y0G7_1_436          LREKTEIDGKDSKRN--SRGHCFIPGVAIDLDGHIGQLPHTAFSMSATST
UniRef90_L9JD22_1_408          LREKREMNAKGSKRNHYSRDHCSIPGVVIDLENHMGQSPQTT--------
UniRef90_F1N617_1_436          LRGKRETDGKDSKRNHYFKGHCSSPGDAIELNNHVGQSPQTAFSLPANCT
UniRef90_F1SV25_1_385          VRGKRESDSEDSKRNHYPRG------------------------------
UniRef90_L5JUH5_1_307          --------------------------------------------------
UniRef90_UPI00020369CA_1_441   KEADQKHFLADTQNADTKTAR--NHLLLEQLRSGRERRILHKFPHQEQ-Q
UniRef90_R4GLX1_1_405          KEADQKHFLAGAQNADTAPRS--FAEMLSQPP-------LQPFP------
UniRef90_UPI00035A19E6_1_466   EEAEKKHFLLVTQNADTASTF--YLRSFAEMPSQTSLQPGTTFPSTSFRQ
UniRef90_F6WA84_1_463          KQENTTTGHQANFEAAVHLGYR--PEMPLGAALYPSLQPEMTFSPPTDCT
UniRef90_G1SG18_1_430          PGTTTTS---RHCPAYAVGTWKQSQDLEPLGEPLTDLHSDSSVRESGGWV
UniRef90_UPI00032B0E64_1_413   P-DTTAS---RHCPVYAAGTWKQSQDLEPSGEPATDLHS--YCRDSDSSV
UniRef90_UPI0002C65D9D_1_412   LGTTTAP---RHCPSYTIGTWEQPQHPRSSGEPLTDFHS--HCRDSDGSV
UniRef90_F7CF28_1_383          LGSTTAP---RHCPSYAIG-------------------------------
UniRef90_Q969M2_1_435          LGTTTAP---RNCPSFAVGTWEQSQDPEPSGEPLTDLHS--HCRDSEGSM
UniRef90_L9JVZ3_1_406          LGKMTAS---RHCPSYAIGTLEKSQDLELSGEPLTDVHS--HC-------
UniRef90_I3M407_1_414          LGATVAS---QHCPSYALRTWEQSQDLESSGEPLTDMRS--HFKGSDGSV
UniRef90_L5JMB7_1_435          LGETTAP---RHCPSYAIGTWEQSYDLQPSEEPLTDFHN--HCRDSNGSM
UniRef90_L8J083_1_417          LGTKMAP---RHCPSHTTGPWEQSQDLQPSGEPLADLHS--HCRHSDGSM
UniRef90_UPI0002BCCFF6_1_421   LGTTMAP---RHCPSRTTGPWEQSQDLQPSGEPLTDLHS--HCRHSDGSM
UniRef90_F1RY20_1_421          LGTTMAS---RHCLSHTTGPWERPQDLKPSGEPLTDLHS--HCRDSNGSR
UniRef90_UPI0002C59166_1_412   LGTEIAP---RHCPSYAIGTWGQSQDRRPSGEALTDLNS--KCRDSDGSV
UniRef90_G3TBX7_1_436          LERTTAP---EHCPSYVPGTWELPQDLKPPGEPLTDLHS--HCKDSDGSV
UniRef90_G3GSS7_1_405          LGTMTAS---PHCPPSTLGTWERSHGQDASGKPLSDLQS--H--------
```

```
UniRef90_UPI0003316158_1_413      LLTAAPP---RQGASYAPGPWEQAQVRHLAGESLTDAQS--LCRDSEGSV
UniRef90_G3WUN4_1_443             ILSRPQLCNLANCPSCTLHPGEQSQSLWNPTRTSIDLTT--HCKTSKDSM
UniRef90_F6W379_1_264             ------------------------------AETTEDEIS--SCKSKKNS-
UniRef90_M7BL54_1_431             ETHKQPQQDIESCPGNEQHIPEPPRNPVQPAKTPTRAGPLEIPQAPRNIL
UniRef90_UPI0002B44362_1_276      -----------------SVVPPPPCLAS----------------------
UniRef90_UPI000298933C_1_429      PAALRNPQRKMSRVSVYKDLSDMSDSAESEPHPTVRKCSFMSRGLSDGKL
UniRef90_H3CDZ2_1_426             PAALKNPARKQSRVSIYKELSDMSDSAESEPHLPARKCSFMSRGLSDGKL
UniRef90_I3IZH7_1_440             PAALRNPQRKQSRVSVCKELSDMSDSPESEHYPTARKCSFMSRGLSEGKL
UniRef90_E7F3P4_1_389             PAALR----KHSR-------------------------------------


UniRef90_UPI0002C691BE_1_358      ------------------------------------------------
UniRef90_F7BKC0_1_358             ------------------------------------------------
UniRef90_Q0VCR2_1_359             ------------------------------------------------
UniRef90_G3TUX5_1_358             ------------------------------------------------
UniRef90_L9JKN2_1_358             ------------------------------------------------
UniRef90_P36382_1_358             ------------------------------------------------
UniRef90_UPI0003315E33_1_350      ------------------------------------------------
UniRef90_UPI000333FB64_1_358      ------------------------------------------------
UniRef90_UPI000334540D_1_357      ------------------------------------------------
UniRef90_L5K3R8_1_358             ------------------------------------------------
UniRef90_G1TQ37_1_358             ------------------------------------------------
UniRef90_UPI0001776F40_1_352      ------------------------------------------------
UniRef90_G3H2Q9_1_358             ------------------------------------------------
UniRef90_Q01231_1_358             ------------------------------------------------
UniRef90_G5BBL6_1_357             ------------------------------------------------
UniRef90_H0WCZ7_1_358             ------------------------------------------------
UniRef90_G3X1A3_1_358             ------------------------------------------------
UniRef90_H0ZTI1_1_370             ------------------------------------------------
UniRef90_P18860_1_369             ------------------------------------------------
UniRef90_G1KPB5_1_370             ------------------------------------------------
UniRef90_K7FWT6_1_369             ------------------------------------------------
UniRef90_F1QL21_1_400             ------------------------------------------------
UniRef90_UPI0002A497DC_1_408      ------------------------------------------------
Input_protein_seq                 ------------------------------------------------
UniRef90_UPI00027FC10C_1_397      ------------------------------------------------
UniRef90_L9JGQ0_1_441             ------------------------------------------------
UniRef90_P28236_1_440             ------------------------------------------------
UniRef90_G5BBL7_1_440             ------------------------------------------------
UniRef90_M3XED1_1_440             ------------------------------------------------
UniRef90_L5K685_1_440             ------------------------------------------------
UniRef90_F6VG96_1_441             ------------------------------------------------
UniRef90_P55917_1_440             ------------------------------------------------
UniRef90_UPI000329268E_1_442      ------------------------------------------------
UniRef90_G3UBH4_1_410             ------------------------------------------------
UniRef90_I3N7I6_1_431             ------------------------------------------------
UniRef90_G3X210_1_429             ------------------------------------------------
UniRef90_F7BI06_1_372             ------------------------------------------------
UniRef90_UPI000333FA9E_1_424      ------------------------------------------------
UniRef90_K7EY71_1_406             ------------------------------------------------
UniRef90_M7BCA4_1_394             ------------------------------------------------
UniRef90_P36381_1_400             ------------------------------------------------
UniRef90_H2MUH4_1_428             ------------------------------------------------
UniRef90_UPI00022B4A0D_1_396      ------------------------------------------------
UniRef90_H3D8V1_1_361             ------------------------------------------------
UniRef90_G3NHJ7_1_420             ------------------------------------------------
UniRef90_Q503J6_1_391             ------------------------------------------------
UniRef90_F6QCF2_1_438             ------------------------------------------------
UniRef90_UPI00034FAB61_1_453      ERGSSRAAS---------------------------------------
UniRef90_UPI0001DE8510_1_439      ------------------------------------------------
UniRef90_H3A837_1_370             ------------------------------------------------
UniRef90_G1KPB7_1_391             ------------------------------------------------
UniRef90_B0S6Z4_1_382             ------------------------------------------------
UniRef90_G1PZ72_1_426             ------------------------------------------------
UniRef90_UPI000155FB82_1_448      ------------------------------------------------
UniRef90_F6U7L2_1_428             ------------------------------------------------
UniRef90_UPI0002C6341D_1_440      ------------------------------------------------
UniRef90_M3Z8M0_1_445             ------------------------------------------------
UniRef90_G1Q1M8_1_411             ------------------------------------------------
UniRef90_G1PYG2_1_394             ------------------------------------------------
UniRef90_L5L5Q1_1_430             ------------------------------------------------
UniRef90_UPI0002BCF55A_1_410      ------------------------------------------------
UniRef90_P41987_1_407             ------------------------------------------------
UniRef90_J9JHI9_1_433             ------------------------------------------------
UniRef90_Q9Y6H8_1_435             ------------------------------------------------
UniRef90_G3QHB2_1_399             ------------------------------------------------
```

```
UniRef90_F7B1T8_1_432          ------------------------------------------------
UniRef90_L9L2D3_1_460          ------------------------------------------------
UniRef90_Q64448_1_417          ------------------------------------------------
UniRef90_G3H8J5_1_417          ------------------------------------------------
UniRef90_UPI0003333DA4_1_405   ------------------------------------------------
UniRef90_I3NC37_1_417          ------------------------------------------------
UniRef90_H0XD37_1_430          ------------------------------------------------
UniRef90_G1U3S9_1_407          ------------------------------------------------
UniRef90_UPI00032AEFC1_1_413   ------------------------------------------------
UniRef90_UPI00034F30A8_1_402   ------------------------------------------------
UniRef90_UPI0003340A67_1_370   ------------------------------------------------
UniRef90_UPI0002234D0E_1_449   ------------------------------------------------
UniRef90_G3TTN5_1_429          ------------------------------------------------
UniRef90_UPI0002C457ED_1_447   ------------------------------------------------
UniRef90_UPI000333D50E_1_427   ------------------------------------------------
UniRef90_UPI0003343FBC_1_423   ------------------------------------------------
UniRef90_F7G5V2_1_446          ------------------------------------------------
UniRef90_G3VY19_1_428          ------------------------------------------------
UniRef90_UPI000155BBAC_1_459   ------------------------------------------------
UniRef90_F7ETY8_1_425          ------------------------------------------------
UniRef90_I3KXW1_1_474          ------------------------------------------------
UniRef90_UPI00022B3AE8_1_449   ------------------------------------------------
UniRef90_UPI0002A48057_1_447   ------------------------------------------------
UniRef90_H2MCD5_1_421          ------------------------------------------------
UniRef90_H2UTF3_1_420          ------------------------------------------------
UniRef90_UPI000298F1F6_1_460   ------------------------------------------------
UniRef90_H3DE08_1_376          ------------------------------------------------
UniRef90_Q8JFD2_1_434          ------------------------------------------------
UniRef90_H2T4Y1_1_438          ------------------------------------------------
UniRef90_H2T4Y2_1_428          ------------------------------------------------
UniRef90_H3BZL0_1_378          ------------------------------------------------
UniRef90_I3K1N0_1_478          ------------------------------------------------
UniRef90_H2LFL5_1_470          ------------------------------------------------
UniRef90_M7BWJ7_1_486          ------------------------------------------------
UniRef90_K7F163_1_492          ------------------------------------------------
UniRef90_H0ZM32_1_512          ------------------------------------------------
UniRef90_H3AH16_1_396          ------------------------------------------------
UniRef90_B0BM18_1_388          ------------------------------------------------
UniRef90_Q4S8R0_1_345          ------------------------------------------------
UniRef90_I3KYH7_1_341          ------------------------------------------------
UniRef90_H2M0G7_1_345          ------------------------------------------------
UniRef90_H3A471_1_360          ------------------------------------------------
UniRef90_Q6GLA5_1_353          ------------------------------------------------
UniRef90_UPI0002A4A1D5_1_346   ------------------------------------------------
UniRef90_S4RXA6_1_456          ------------------------------------------------
UniRef90_P16863_1_360          ------------------------------------------------
UniRef90_H2LU51_1_404          ------------------------------------------------
UniRef90_I3KYL3_1_401          ------------------------------------------------
UniRef90_G3NC44_1_402          ------------------------------------------------
UniRef90_H2SSL2_1_392          ------------------------------------------------
UniRef90_UPI0002C46B7E_1_433   WKPKRLNATWGPFTEDHNQRLPP-------------------------
UniRef90_G3ULF1_1_432          WKPKRLSATWRPFTEDQNQRLPP-------------------------
UniRef90_C9IYK1_1_407          ------------------------------------------------
UniRef90_H0Y0G7_1_436          WKPRWLSAAWGPSTEDENRGSPS-------------------------
UniRef90_L9JD22_1_408          ------------------------------------------------
UniRef90_F1N617_1_436          WKPKWPRATRGPSAESENQALPP-------------------------
UniRef90_F1SV25_1_385          ------------------------------------------------
UniRef90_L5JUH5_1_307          ------------------------------------------------
UniRef90_UPI00020369CA_1_441   QETEQPQREPRPTAPKADTRHPSR------------------------
UniRef90_R4GLX1_1_405          ------------------------------------------------
UniRef90_UPI00035A19E6_1_466   QALAESTGAPTNSLLKNNDRRQSSFSVNKAQIPYNADVKNSSRPDTSD
UniRef90_F6WA84_1_463          RTARSLSDPWNGSTGGLQSRGSPPRGNLRRQSRGSTGRPRALSRAD--
UniRef90_G1SG18_1_430          DKSR-------PGSRKASFLSR--------------------------
UniRef90_UPI00032B0E64_1_413   R-----------------------------------------------
UniRef90_UPI0002C65D9D_1_412   ------------------------------------------------
UniRef90_F7CF28_1_383          ------------------------------------------------
UniRef90_Q969M2_1_435          RESGVWIDRSRPGSRKASFLSR--------------------------
UniRef90_L9JVZ3_1_406          ------------------------------------------------
UniRef90_I3M407_1_414          R-----------------------------------------------
UniRef90_L5JMB7_1_435          RESGVWTDRSCSGSRKASFLSR--------------------------
UniRef90_L8J083_1_417          TDSR--------------------------------------------
UniRef90_UPI0002BCCFF6_1_421   RESRAQTD----------------------------------------
UniRef90_F1RY20_1_421          RESRVQTD----------------------------------------
UniRef90_UPI0002C59166_1_412   ------------------------------------------------
UniRef90_G3TBX7_1_436          RESGVWIDRSRPGSRKASFLSR--------------------------
UniRef90_G3GSS7_1_405          ------------------------------------------------
```

```
UniRef90_UPI0003316158_1_413      R-----------------------------------------------
UniRef90_G3WUN4_1_443             TERGSYLGSCKASFLSRASESSDSR-----------------------
UniRef90_F6W379_1_264             ------------------------------------------------
UniRef90_M7BL54_1_431             RKHSR-------------------------------------------
UniRef90_UPI0002B44362_1_276      ------------------------------------------------
UniRef90_UPI000298933C_1_429      SSPSDSTD----------------------------------------
UniRef90_H3CDZ2_1_426             SNPSDSADS---------------------------------------
UniRef90_I3IZH7_1_440             ASPSDSGDCQSGTDNAS-------------------------------
UniRef90_E7F3P4_1_389             ------------------------------------------------
```
